# Diagnostic evaluation of a deep learning model for optical diagnosis of colorectal cancer

**DOI:** 10.1038/s41467-020-16777-6

**Published:** 2020-06-11

**Authors:** Dejun Zhou, Fei Tian, Xiangdong Tian, Lin Sun, Xianghui Huang, Feng Zhao, Nan Zhou, Zuoyu Chen, Qiang Zhang, Meng Yang, Yichen Yang, Xuexi Guo, Zhibin Li, Jia Liu, Jiefu Wang, Junfeng Wang, Bangmao Wang, Guoliang Zhang, Baocun Sun, Wei Zhang, Dalu Kong, Kexin Chen, Xiangchun Li

**Affiliations:** 1 Department of Endoscopic Diagnosis and Therapy, National Clinical Research Center for Cancer, Key Laboratory of Molecular Cancer Epidemiology of Tianjin, Tianjin’s Clinical Research Center for Cancer, Key Laboratory of Cancer Prevention and Therapy, Tianjin Medical University Cancer Institute and Hospital, Tianjin Medical University, Tianjin, China; 2 Department of Colorectal Cancer, National Clinical Research Center for Cancer, Key Laboratory of Molecular Cancer Epidemiology of Tianjin, Tianjin’s Clinical Research Center for Cancer, Key Laboratory of Cancer Prevention and Therapy, Tianjin Medical University Cancer Institute and Hospital, Tianjin Medical University, Tianjin, China; 3 Department of Pathology, National Clinical Research Center for Cancer, Key Laboratory of Molecular Cancer Epidemiology of Tianjin, Tianjin’s Clinical Research Center for Cancer, Key Laboratory of Cancer Prevention and Therapy, Tianjin Medical University Cancer Institute and Hospital, Tianjin Medical University, Tianjin, China; 4 General Surgery Department, Ningbo First Hospital, Zhejiang University Ningbo Hospital, Ningbo, China; 5 Anorectal Department, Nanyang Hospital of Traditional Chinese Medicine, Nanyang, China; 6 General Surgery Department, General Hospital of Tianjin Medical University, Tianjin Medical University, Tianjin, China; 7 Department of Maxillofacial and Otorhinolaryngology Oncology, National Clinical Research Center for Cancer, Key Laboratory of Molecular Cancer Epidemiology of Tianjin, Tianjin’s Clinical Research Center for Cancer, Key Laboratory of Cancer Prevention and Therapy, Tianjin Medical University Cancer Institute and Hospital, Tianjin Medical University, Tianjin, China; 8 Tianjin Cancer Institute, National Clinical Research Center for Cancer, Key Laboratory of Molecular Cancer Epidemiology of Tianjin, Tianjin’s Clinical Research Center for Cancer, Key Laboratory of Cancer Prevention and Therapy, Tianjin Medical University Cancer Institute and Hospital, Tianjin Medical University, Tianjin, China; 9 Department of Pathology, Tianjin First Central Hospital, Tianjin, China; 10 Department of Gastroenterology and Hepatobiliary Surgery, People’s First Hospital of Shangqiu, Shangqiu, China; 11 Department of Gastroenterology, General Hospital of Tianjin Medical University, Tianjin Medical University, Tianjin, China; 12 Department of Digestion and Endoscopy, Tianjin First Central Hospital, Tianjin, China; 13 Wake Forest Baptist Comprehensive Cancer Center, Wake Forest Baptist Medical Center, Winston-Salem, NC USA; 14 Department of Cancer Biology, Wake Forest School of Medicine, Winston-Salem, NC USA; 15 Department of Epidemiology and Biostatistics, National Clinical Research Center for Cancer, Key Laboratory of Molecular Cancer Epidemiology of Tianjin, Tianjin’s Clinical Research Center for Cancer, Key Laboratory of Cancer Prevention and Therapy, Tianjin Medical University, Tianjin, China

**Keywords:** Machine learning, Colorectal cancer, Gastroenterology

## Abstract

Colonoscopy is commonly used to screen for colorectal cancer (CRC). We develop a deep learning model called CRCNet for optical diagnosis of CRC by training on 464,105 images from 12,179 patients and test its performance on 2263 patients from three independent datasets. At the patient-level, CRCNet achieves an area under the precision-recall curve (AUPRC) of 0.882 (95% CI: 0.828–0.931), 0.874 (0.820–0.926) and 0.867 (0.795–0.923). CRCNet exceeds average endoscopists performance on recall rate across two test sets (91.3% versus 83.8%; two-sided t-test, p < 0.001 and 96.5% versus 90.3%; p = 0.006) and precision for one test set (93.7% versus 83.8%; p = 0.02), while obtains comparable recall rate on one test set and precision on the other two. At the image-level, CRCNet achieves an AUPRC of 0.990 (0.987–0.993), 0.991 (0.987–0.995), and 0.997 (0.995–0.999). Our study warrants further investigation of CRCNet by prospective clinical trials.

## Abstract (web summary)

Colonoscopy is the most commonly used tool to screen for colorectal cancer (CRC). Here, the authors develop a deep learning model to perform optical diagnosis of CRC by training on a large data set of white-light colonoscopy images and achieve endoscopist-level performance on three independent datasets.

## Introduction

Colorectal cancer (CRC) ranks the second leading cause of cancer-related death and the third most common cancer types worldwide^1^. Colonoscopy is the most frequently used tool to screen for CRC^2,3^, offering direct biopsy of intestinal tumor masses for pathological diagnosis. The advantage of colonoscopic screening is its ability to detect precancerous lesions and CRC at early stage, during which surgical removal is often curative. Colonoscopic screening has contributed to reduced mortality of CRC in observational studies, where ~80% of CRC can be prevented by polypectomy^4,5^. Comparative studies have shown that prompt treatment of severe atypical hyperplasia or CRC at early stage prolonged overall survival^6–8^. In addition, incomplete biopsy often leads to misdiagnosis of early CRC as mild or moderate dysplasia, subsequently leading to inappropriate treatment^9–11^. Therefore, accurate differentiation between malignant and benign lesions under colonoscopy is medically important to select optimal treatment regimen, avoid inappropriate endoscopic resection, and improve cost-effectiveness. A key aspect of endoscopists is the differential diagnosis of colonoscopic lesions, according to NBI International Colorectal Endoscopic (NICE)^12^ and Workgroup serrated polyps and polyposis (WASP)^13^ classification systems. The NICE criterion is built upon features such as staining, vascular and surface patterns that were derived from narrow-band imaging (NBI). The WASP classification system can perform optical diagnosis of hyperplastic polyps, adenomas, and sessile serrated adenomas/polyps. However, to our knowledge, these two criteria cannot be used to perform optical diagnosis of benignity and malignancy for colonoscopic lesions using white-light imaging modality.

The 5-year overall survival rate of patients in USA diagnosed with CRC ranges considerably from 90.1 for patients at stages I and II, 69.2 for patients at stage III to 11.7% for patients at stage IV^14^. In this point of view, an artificial intelligence model that can identify CRC patients at early stage would be useful for earlier intervention.

Deep learning models have seen wide application in medical imaging data interpretation since AlexNet won 2012 ImageNet competition^15^. Recently, we reported that a deep learning model achieved similar sensitivity, and improved specificity and accuracy in detecting patients with thyroid cancer as skilled radiologists^16^. Deep learning models have also been demonstrated to improve endoscopists’ performance in the detection of polyp or adenoma^17,18^ and upper gastrointestinal cancer^19^. Ahmad et al. provided a thorough review on studies related to application of artificial intelligence in computer-aided diagnosis in colonoscopy^20^. Chen et al. used a small number of 2157 images obtained from NBI to develop deep learning model to perform optical diagnosis of diminutive hyperplastic and neoplastic polyps without independent test set^17^.

Until recently, there is still lack of study using deep learning to analyze large-scale colonoscopic images to perform optical diagnosis of CRC. Such a deep learning model could aid endoscopists to distinguish malignancy and benignity of colorectal lesions, which can improve the efficiency of colonoscopy. Herein, we develop a deep learning model termed CRCNet by training on by far the largest number of colonoscopic images (*n* = 464,105) from 12,179 patients obtained from Tianjin Cancer Hospital (TCH) and validate its performance on 2263 patients from an internal and two external test sets. We use pathological examination as gold standard to train and evaluate CRCNet. The performance of CRCNet is assessed and compared against five endoscopists at both patient level and image level. In addition, we employ weakly supervised algorithm to pinpoint neoplastic lesions on the image, which could provide visual explanation for decision made by CRCNet to justify its prediction. The purpose of this study is to investigate the performance of CRCNet for optical diagnosis of CRC. We train CRCNet with large volume of colonscopic imaging data and systematically evaluate its performance on three independent test sets. We show that CRCNet achieves endoscopist level in optical diagnosis of CRC across these three test sets.

## Results

### Baseline characteristics of training and test datasets

**Fig. 1:**
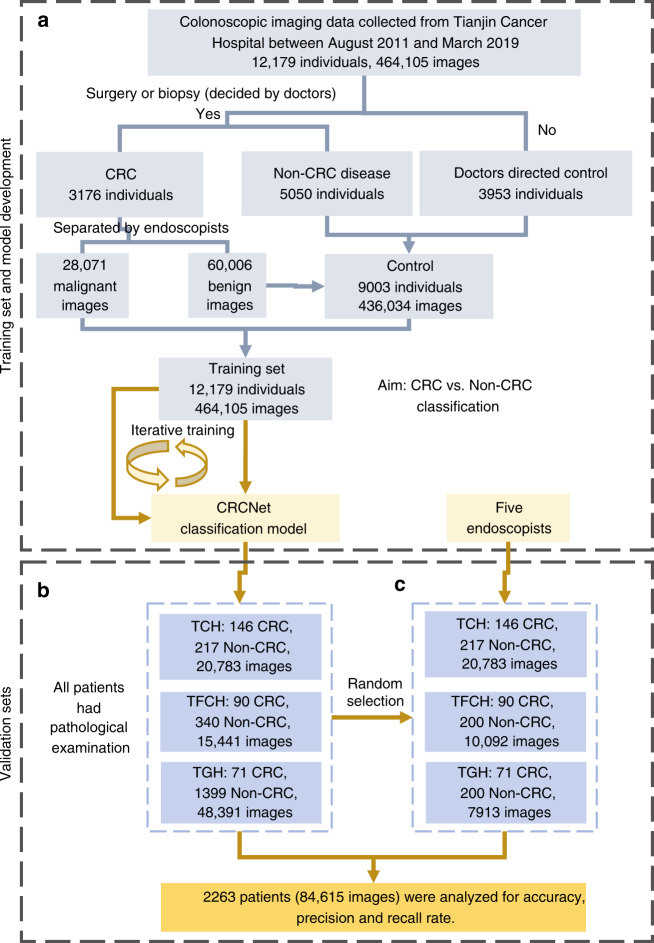
Flowchart depicting the development and evaluation of CRCNet. **a** Model development consisted data curation and CRCNet training. **b** Evaluation of CRCNet on three test sets. **c** Comparison between CRCNet and five endoscopists on a subset of randomly selected cases. All CRC patients and 5050 control patients in the training set and all patients in three test sets have surgical specimen or biopsy for pathological evaluation.

We obtained 464,105 images from 12,179 patients between August 2011 and March 2019 at TCH as training set. We subsequently assembled three test sets that consisted of 20,783 images from 363 patients between April 2019 and May 2019 at TCH, 15,441 images from 430 patients between January 2018 and February 2019 at Tianjin First Central Hospital (TFCH), and 48,391 images from 1470 patients between January 2018 and December 2018 at Tianjin General Hospital (TGH), respectively. All CRC patients (*n* = 3176) and 56.1% (5050/9003) of control patients in training set and all patients in three test sets have pathological examination results. The training set consisted of 3176 CRC patients and 9003 controls. Male sex accounted for 62.5% (1985/3176) in CRC patient group versus 54.5% (4909/9003) in the control group. Ages were 60 (53–67) in the CRC patient group versus 57 (49–64) for the control group. For CRC patients, tumors from different sites were included: ascending colon (19.9%, *n* = 631), transverse colon (4.5%, *n* = 143), descending colon (5.6%, *n* = 178), sigmoid colon (17.7%, *n* = 561), and rectum (52.4%, *n* = 1663). There were 11.4% of CRC patients at stage I, 44.3% at stage II, 7.3% at stage III, and 2% at stage IV; whereas 35.7% patients had no TNM stage information because they did not receive surgical resection but only colonoscopy-guided biopsy (Table 1). Besides normal mucosa, multiple benign diseases were encompassed in the control group, including adenoma, hyperplastic polyps, sessile serrated adenomas/polyps, inflammatory bowel disease, and chronic mucosal inflammation (Supplementary Table 1). There were 146 CRC patients and 217 controls in TCH test set (*n* = 363), 90 CRC patients and 340 controls in TFCH test set (*n* = 430), and 71 CRC patients and 1399 controls in TGH test set (*n* = 1470). The additional detailed number of patients with regards to sex, age, tumor site, TNM stage, and a flowchart depicting these processes were provided in Table 1 and Fig. 1, respectively.

**Table 1:** Baseline characteristics of training set and three test sets.

	TCH training set		TCH test set		TFCH test set		TGH test set	
	CRC	Non-CRC	CRC	Non-CRC	CRC	Non-CRC	CRC	Non-CRC
Patients	3176	9003	146	217	90	340	71	1399
Images	28,071	436,034	7485	13,298	2576	12,865	1614	46,777
Male sex	1985	4909	86	132	59	220	35	780
Images	17,697	245,936	4599	8151	1618	8351	760	26,510
Female sex	1191	4094	60	85	31	120	36	619
Images	10,734	190,071	2886	5147	958	4514	854	20,267
Age (years)	60 (53–67)	57 (49–64)	61 (53–66)	59 (52–66)	63 (53.3–72)	58.5 (50–65)	66 (59–74)	58 (47.5–65)
Age ≤ 60 years male	1037 (32.7%)	3004 (33.4%)	42 (28.8%)	71 (32.7%)	20 (22.2%)	138 (40.6%)	9 (25.7%)	481 (61.7%)
Age > 60 years male	948 (29.8%)	1905 (21.2%)	44 (30.1%)	61 (28.1%)	39 (43.3%)	82 (24.1%)	26 (74.3%)	299 (38.3%)
Age ≤ 60 years female	604 (19%)	2672 (29.7%)	28 (19.2%)	53 (24.4%)	15 (16.7%)	52 (15.3%)	11 (30.6%)	342 (55.3%)
Age > 60 years female	587 (18.5%)	1422 (15.8%)	32 (21.9%)	32 (14.7%)	16 (17.8%)	68 (20%)	25 (69.4%)	277 (44.7%)
Tumor site^a^								
Ascending colon	631 (19.9%)		15 (10.3%)		20 (22.2%)		19 (26.8%)	
Transverse colon	143 (4.5%)		17 (11.6%)		8 (8.9%)		9 (12.7%)	
Descending colon	178 (5.6%)		11 (7.5%)		0		7 (9.9%)	
Sigmoid colon	561 (17.7%)		36 (24.7%)		29 (32.2%)		19 (26.8%)	
Rectum	1663 (52.4%)		67 (45.9%)		33 (36.7%)		17 (23.9%)	
TNM staging^a^								
I	362 (11.4%)		15 (10.3%)		9 (10.0%)		5 (7%)	
II	1407 (44.3%)		24 (16.4%)		9 (10.0%)		15 (21.1%)	
III	231 (7.3%)		34 (23.3%)		12 (13.3%)		9 (12.7%)	
IV	62 (2%)		3 (2.1%)		1 (1.1%)		1 (1.4%)	
Biopsy pathology	1114 (35.7%)		70 (47.9%)		59 (65.6%)		41 (57.7%)	

^a^Tumor site and TNM staging were reported for CRC patients only.

### Performance of CRCNet on three independent test sets

We trained CRCNet iteratively and evaluated the performance of the best model on three test sets (see “Methods”). Malignancy score for each patient was calculated according to Eq. (1). We found that CRCNet achieved high performance in identifying CRC patients: for the TCH test set, area under the PR curve (AUPRC) was 0.882 (95% CI: 0.828–0.931), accuracy was 87.3% (83.5–90.6%), recall rate was 90.4% (0.844–0.947), specificity was 85.3% (79.8–89.7%), and F1 was 85.2%; for TFCH test set, AUPRC was 0.874 (0.820–0.926), accuracy was 91.6% (88.6–94.1%), recall rate was 78.9% (69.0–86.8%), specificity was 95.0% (92.1–97.1%), and F1 was 79.8%; and for TGH test set, AUPRC was 0.867 (0.795–0.923), accuracy was 98.0% (97.2–98.7%), recall rate was 74.6% (62.9–84.2%), specificity was 99.2% (98.6–99.6%), and F1 was 78.5% (Table 2 and Supplementary Fig. 1). The area under the receiver-operating characteristic (AUROC) curve was 0.930 (0.903–0.956) for TCH, 0.961 (0.943–0.979) for TFCH, and 0.989 (0.980–0.997) for TGH test sets (Supplementary Fig. 2). The other classification metrics such as precision, negative predictive value and kappa coefficient were provided in Table 2.

**Table 2:** Classification metrics of CRCNet at the patient level.

	The performance of CRCNet across three test sets		
Performance metrics	Tianjin Cancer Hospital (*n* = 363)	Tianjin First Central Hospital (*n* = 430)	Tianjin General Hospital (*n* = 1470)
Accuracy (95% CI)	0.873 (0.835–0.906)	0.916 (0.886–0.941)	0.980 (0.972–0.987)
Recall rate (95% CI)	0.904 (0.844–0.947)	0.789 (0.690–0.868)	0.746 (0.629–0.842)
Specificity (95% CI)	0.853 (0.798–0.897)	0.950 (0.921–0.971)	0.992 (0.986–0.996)
Precision (95% CI)	0.805 (0.736–0.863)	0.807 (0.709–0.883)	0.828 (0.713–0.911)
Negative predicted value (95% CI)	0.930 (0.885–0.961)	0.944 (0.915–0.966)	0.987 (0.980–0.992)
Kappa^a^	0.742	0.745	0.775
F_1_^b^	0.852	0.798	0.785

^a^Measures the agreement between predicted classification with pathological report.

^b^Harmonic average of the precision and recall rate.

Notably, the predicted malignancy scores of CRC patients at early stage (I and II) and advanced stage (III and IV) are comparable (mean 0.679 versus 0.689; two-sided *t*-test, *p* = 0.843; Supplementary Fig. 3). For 36 false negative cases in three test sets, 2 of them who had received radiotherapy or chemotherapy exhibited complete response; 4 of them underwent endoscopic submucosal dissection and en bloc removal. Colonic lesions were not discernible on the colonoscopic images from these patients. The other two false negatives were due to the absence of lesions on the clipped images. Among the remaining 30 cases, 10 of them had constrictive lesion, necrotic ooze attached to the surface, intussusception, or severe colon melanosis, which led to poor exposure of the lesion; ulcer in eight cases led to the absence of lesions on the clipped images. Preoperative management such as marking with metal titanic clips, submucosal injection of nanocarbon and biopsy contributed to false negative prediction in the other six cases. The rest six cases are putative false negatives made by the model. The gradient-weighted class activation mapping (Grad-CAM) images of these 36 cases were provided as Supplementary Data 1.

With respect to optical diagnosis of CRC by left-sided (descending and sigmoid colon), right (ascending and transverse colon), and rectal locations, the AUPRC values of CRCNet were 0.729 (0.601–0.866), 0.622 (0.483–0.79), and 0.788 (0.699–0.874) for TCH test set, 0.743 (0.609–0.866), 0.734 (0.588–0.859), and 0.748 (0.594–0.887) for TFCH test set, and 0.572 (0.401–0.815), 0.691 (0.514–0.858), and 0.728 (0.558–0.942) for TGH test set. The precision–recall (PR) curves were provided in Supplementary Fig. 4. The AUROC values were ranged from 0.927 to 0.939 for TCH test set, 0.949 to 0.975 for TFCH test set, and 0.978 to 0.996 for TGH test set. The receiver-operating characteristic (ROC) curves were provided in Supplementary Fig. 5.

### Performance of CRCNet versus five endoscopists

**Fig. 2:**
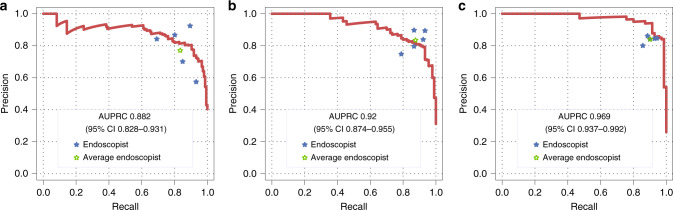
Performance of CRCNet versus endoscopists in identifying CRC. Precision–recall curves of CRCNet on **a** TCH test set, **b** TFCH test set, and **c** TGH test sets. Area under the precision–recall curve and associated 95% confidence intervals are included. Blue stars depict precision and recall rate of each individual endoscopist and green stars are average performance of these five endoscopists.

All CRC patients and a random subset of 200 controls from TFCH and TGH test sets, and all patients from TCH test set were used to evaluate the performance of CRCNet versus a group of five skilled endoscopists. The total number of images read and interpreted by each endoscopist were 38,788. CRCNet achieved an AUPRC of 0.882 (95% CI 0.828–0.931) for TCH test set, 0.920 (95% CI 0.874–0.955) for TFCH test set, and 0.969 (95% CI 0.937–0.992) for TGH test set (Fig. 2). As compared with endoscopists, CRCNet achieved an F1 metric of 0.852, 0.857, and 0.928 versus 0.768, 0.878, and 0.873 on these three subsets from test sets. The other classification metrics such as accuracy, recall rate, specificity, precision, negative predictive value, and kappa coefficient were provided in Table 3. Detailed classification metrics for each endoscopists were provided in Supplementary Table 2. The inter-rater agreement rate for this group of five experienced endoscopists was 56.7% (206/363, Fleiss’ Kappa 0.58; two-sided *z*-test, *p* < 0.001) in TCH test set, 76.6% (222/290, Fleiss’ Kappa 0.75; two-sided *z*-test, *p* < 0.001) in TFCH test set, and 88.2% (239/271, Fleiss’ Kappa 0.87; two-sided *z*-test, *p* < 0.001) in TGH test set. The average precision and recall rate of this group of five endoscopists were situated below PR curves in TCH and TGH test sets (Fig. 2a, c), while it was marginally situated above PR curve in TFCH test set (Fig. 2b). At the average precision level of this group of five endoscopists, CRCNet obtained higher recall rate in TCH (91.3% versus 83.8%; two-sided binomial test, *p* < 0.001) and TGH (96.5% versus 90.3%; two-sided binomial test, *p* = 0.006) test sets, and comparable recall rate in TFCH (82.9% versus 87.6%; two-sided binomial test, *p* = 0.29) test set. Whereas at the average recall rate level of this group of five endoscopists, CRCNet obtained higher precision in TGH (93.7% versus 83.8%; two-sided binomial test, *p* = 0.02) test set, and comparable precision in TCH (81.3% versus 77.9%; two-sided binomial test, *p* = 0.32) and TFCH (81.1% versus 83.4%; two-sided binomial test, *p* = 0.52) test sets.

**Table 3:** Classification metrics of endoscopists versus CRCNet.

	The performance of endoscopists and CRCNet					
	Tianjin Cancer Hospital (*n* = 363)		Tianjin First Central Hospital (*n* = 290)		Tianjin General Hospital (*n* = 271)	
Performance metrics	Endoscopist^a^	CRCNet	Endoscopist^a^	CRCNet	Endoscopist^a^	CRCNet
Accuracy (95% CI)	0.824 (0.781–0.861)	0.873 (0.835–0.906)	0.928 (0.891–0.955)	0.903 (0.863–0.935)	0.934 (0.897–0.960)	0.963 (0.933–0.982)
Recall rate (95% CI)	0.849 (0.781–0.903)	0.904 (0.844–0.947)	0.867 (0.779–0.929)	0.933 (0.861–0.975)	0.900 (0.805–0.959)	0.914 (0.823–0.968)
Specificity (95% CI)	0.912 (0.867–0.946)	0.853 (0.798–0.897)	0.920 (0.873–0.954)	0.890 (0.838–0.930)	0.940 (0.898–0.969)	0.980 (0.950–0.995)
Precision (95% CI)	0.842 (0.764–0.902)	0.805 (0.736–0.863)	0.838 (0.751–0.905)	0.792 (0.703–0.865)	0.844 (0.744–0.917)	0.941 (0.856–0.984)
Negative predicted value (95% CI)	0.880 (0.825–0.924)	0.930 (0.885–0.961)	0.941 (0.899–0.969)	0.967 (0.930–0.988)	0.964 (0.928–0.986)	0.970 (0.937–0.989)
Kappa^b^	0.622	0.742	0.82	0.785	0.828	0.903
F_1_^c^	0.768	0.852	0.878	0.857	0.873	0.928

^a^The median value of five endoscopists.

^b^Measures the agreement between predicted classification with pathological report.

^c^Harmonic average of the precision and recall rate.

### Performance of CRCNet at image level

**Fig. 3:**
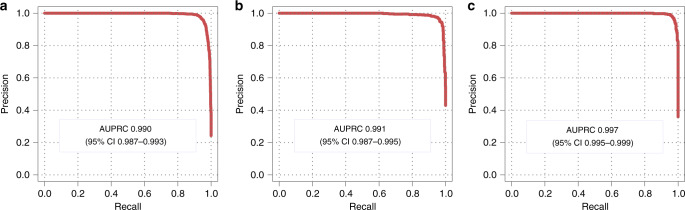
Performance of CRCNet in identifying malignant colonoscopic images. Precision–recall curves of CRCNet on **a** TCH test set, **b** TFCH test set, and **c** TGH test sets. Area under the precision–recall curve and associated 95% confidence intervals are included.

We also measured the classification performance of CRCNet at the image level on images of CRC patients from three test sets (see “Methods”). CRCNet achieved high performance in detecting consensus malignant images (Fig. 3): for the TCH test set, AUPRC was 0.990 (95% CI 0.987–0.993) and F1 was 95.6%; for TFCH test set, AUPRC was 0.991 (95% CI 0.987–0.995) and F1 was 96.3%; and for TGH, AUPRC was 0.997 (95% CI 0.995–0.999) and F1 was 97.3%. The other classification metrics such as accuracy, recall rate, specificity, precision, negative predictive value, and kappa coefficient were provided in Supplementary Table 3.

### Visual explanation of decision made by CRCNet

**Fig. 4:**
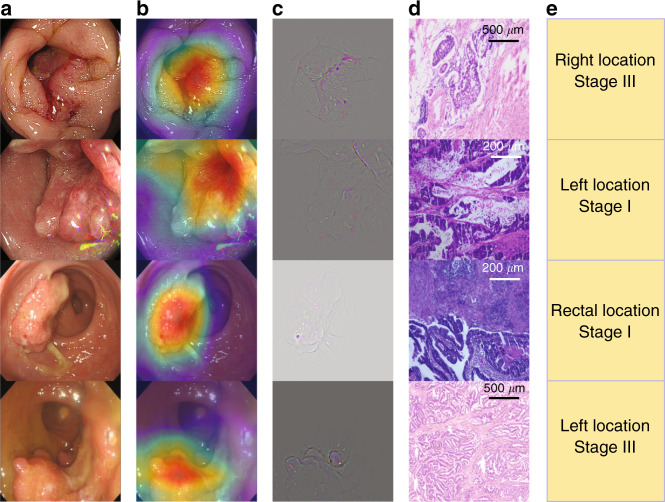
Exemplified class activation maps. **a** Raw colonoscopic images. **b** Gradient-weighted class activation map. **c** Guided gradient-weighted class activation map. **d** Haematoxylin–eosin staining images with scale bars. The length of scale bar was shown above the bar. **e** Tumor location and TNM stage.

We used Grad-CAM algorithm^21^ to identify image regions contributed the most to the prediction made by CRCNet. Representative examples of malignant colonoscopic images with accompanying saliency heatmaps highlighting features most influenced CRCNet prediction was shown in Fig. 4 and Supplementary Data 2. The Grad-CAM heatmaps of flat and sessile serrated polyps were provided as Supplementary Data 3 and 4. In addition, we asked five endoscopists to inspect 255 randomly selected colonoscopic images and their accompanying saliency heatmaps. The full list of these 255 images were provided in Supplementary Data 5. The percentage of these 255 saliency heatmaps for which these five endoscopists agreed that the heatmaps captured the regions of malignant lesions was 94.3% (95% CI: 91.4–97.1%). The accuracy assessed by each endoscopist was shown in Supplementary Table 4.

## Discussion

Results from this study showed that CRCNet model achieved high precision and recall rates in identifying CRC patients as compared with a group of five skilled endoscopists. CRCNet achieved consistent and robust performance across three test sets with significant improvement in precision on two test sets and recall rate on one test set. At the image level, CRCNet performed satisfactorily in distinguishing between malignancy and benignity of colonic lesions. In addition, endoscopists found the evidence-based visual explanation derived from CRCNet useful for routine clinical practice.

Improvement in medical imaging technique and endoscopic classification system such as WASP have facilitated the optical diagnosis of benign lesions, however, optical diagnosis of malignant lesions is also an important clinical application and remains challenging. For example, invasive tumor often needs surgical treatment, whereas precancerous disease such as adenoma requires endoscopic submucosal dissection. Therefore, it is important to devise new ways to aid endoscopists in differentiating benign lesions from malignant ones on white-light images, which is the most commonly used imaging modality in routine clinical practice. In addition, white-light imaging mode can avoid false positive caused by poor intestinal preparation under NBI to some extent.

In recent studies, deep learning has been widely applied in endoscopy. Previous studies have developed deep learning models on images from colonoscopy for detecting polyps or adenomas^17,18,22^. Our study was dedicated to optical diagnosis of CRC instead of lesion detection as conducted by aforementioned studies. Optical diagnosis of CRC has previously been investigated by Mori et al. by training on image features extracted from endocytoscopy^23^, which can provide morphological images of the nuclei and gland duct lumens that beyond the capability of colonoscopy. Takemura et al. developed HuPAS software for predicting histology of colorectal tumors from 1519 cut-out images from magnifying NBI images^24^. To the best of our knowledge, we developed CRCNet for optical diagnosis of CRC using by far the largest number of colonoscopic images from standard white-light imaging modality. CRC is initially identified by endoscopists during colonoscopic examination based on tumor surface features. It is difficult to accurately recognize all malignant lesions and there is a low interendoscopists agreement rate, which was also reflected by the varying agreement rates among endoscopists in test sets in this study (range from 56.7 to 88.2%). This is likely due to increased difficulty in identifying noncancerous patients in TCH cohort because patients came to TCH are often suspected to be cancerous. This was reflected by higher proportion of CRC patients in test set from TCH (40.2%, *n* = 146/363) as compared with those from TFCH (20.9%, *n* = 90/430) and TGH (4.8%, *n* = 71/1470). On the contrary, CRCNet can make consistent interpretation once deployed.

CRCNet has the potential to reduce the reliance on expertise of colonoscopists for optical diagnosis of CRC and improve diagnostic consistency. One advantage of our study was the inclusion of diverse noncancerous diseases (i.e., polyps, adenomas, chronic mucosal inflammation, and inflammatory bowel disease) in control group and employment of pathological examination as gold standard. Therefore, CRCNet could learn noncancerous features that often complicate CRC diagnosis, presumably increasing model performance and avoiding verification bias. Meanwhile, the performance of endoscopists in identifying CRC depends on their working experience and often varies considerably given that human-derived diagnostic systems are often established by expert consensus and subjected to human variation^20^, whereas CRCNet can provide objective optical diagnosis.

Another advantage of CRCNet is that it can report results immediately and consistently on the graphics processing unit thus facilitating real-time CRC detection and decreasing the workload, inconsistency, and misdiagnosis. In addition, CRCNet can overcome inherent limitations of endoscopists such as perceptual bias and visual fatigue^17^. CRCNet showed high degree of fidelity and uniformity in differentiating malignant lesions from benign lesions at image level, suggesting that it could serve as a second-read tool during colonoscopic examination. The prognosis of patients with CRC is closely associated with pathological stage. The comparable predicted malignancy scores of CRC patients at early stage (I and II) and advanced stage (III and IV) (mean 0.679 versus 0.689; two-sided *t*-test, *p* = 0.843) suggested that CRCNet performs equally good in detecting CRC patients at early stage as those at advanced stage. For false positives with high prediction malignancy scores and also interpreted as CRC patients by a group of five endoscopists, it is likely due to incomplete biopsy during colonoscopy examination. This highlighted that CRCNet might complement endoscopists in optical diagnosis. Additional follow-up evaluation for these patients are required. Besides, the visual explanation derived from CRCNet can further provide evidence-based classification to assist endoscopists in interpreting the images. This can justify the predictions made by CRCNet, especially when there are unreliable predictions. However, the accuracy of CRCNet to pinpoint small tumors would decrease as Grad-CAM algorithm only performs well when the class being detected covers a big portion of the image.

Integration of CRCNet into colonoscopy interpretation system can help endoscopists speed up interpretation process. A second read from CRCNet could augment the capability of endoscopists to manage patients at high risk of CRC. Marginal improvement in CRCNet to discriminate between malignant and benign lesions could reduce unnecessary biopsy. Using the white-light imaging modality and not having revert to NBI would be more clinically relevant and potentially more translational as it relies on images seen in routine colonoscopy. CRCNet can further expand the recognition of early colorectal lesions by white light, especially for community hospitals without NBI equipment. Although CRCNet was developed with white-light imaging data, it could to some extent correctly classify sessile polyps as benign, which is considered to be better recognized on NBI imaging mode. However, the aforementioned clinical diagnostic validity conferred by CRCNet requires further investigation in clinical trials.

There are several limitations of this study. Firstly, CRCNet was trained only on static images from a single medical center, it may not be able to capture all the real-world data variation in relation to different devices and skills of endoscopists. In daily clinical practice, optical diagnosis of colonoscopic lesions are evaluated in a real-time scenario and image quality would be affected by operational fluctuation. This will deteriorate the performance of CRCNet. On the other hand, it might be easier to characterize colonoscopic lesions in real-time colonoscopy as we are able to visualize lesions from multiple perspectives, thus potentially increasing the performance of optical diagnosis^13^. To overcome this limitation, we employed extensive data augmentation during model training, the impact of lacking training data from other centers could be mitigated and image variations were also increased. This was demonstrated by comparable, robust, and generalizable performance of CRCNet on external test sets. Results from Urban et al. demonstrated that deep learning model trained on static images could achieve high accuracy in real-time colonoscopy settings^25^. Secondly, the current model can only distinguish between malignancy and benignity. In this study, we did not include other rare malignant diseases observed by colonoscopy such as sarcoma, melanoma, gastrointestinal stromal tumor, lymphoma, and carcinoid tumor because of their limited number of cases. Future work will be focused on expanding our model to further classify whether the detected benign intracavity lesion is adenoma, hyperplastic polyps, or sessile serrated polyps/adenomas. Thirdly, the enrolled patients in training set and test sets were mainly northern Chinese, the performance of CRCNet on other ethnic groups remains to be tested. Fourthly, the improvement of clinical outcomes brought by CRCNet remains to be determined as this is a retrospective study. In addition, we did not consider other types of data, for example, age, sex, family history, and tumor site, beyond colonoscopic images.

Although CRCNet could achieve satisfactory performance, it does not necessarily mean improved clinical outcomes. Class imbalance is a common challenge in medical area. It is difficult to obtain positive samples in that these samples were underrepresented in real-world scenario and imaging database. The ratio of positive samples to negative samples was 6.4% in training set. We addressed this issue by using focal loss for training. The focal loss focuses training on hard-classified examples while prevents the well-classified negative examples from overwhelmingly affecting the model^26^.

Future prospective studies should be conducted to compare clinical efficacy of colonoscopy with or without assistance of CRCNet. Given the unbalanced medical resource in many countries including China, such a model may benefit community hospitals in rural areas. In the future, we intend to associate pathological findings such as the depth of tumor invasion, vascular thrombus, perineural invasion, lymph node, and distant metastasis with features of extracted colonoscopic images, in order to predict the preoperative CRC staging and aid selection of alternative treatments.

In summary, CRCNet can achieve high performance in differentiating CRC from benign diseases such as adenomas and polyps. CRCNet achieved consistent and robust performance in identifying CRC patients across three test sets. Its performance was comparable with a group of five skilled endoscopists. Prospective randomized clinical trials are required to test the performance of this model in real-world clinical settings.

## Methods

### Study design and participants

We did retrospective, multicohort, diagnostic study using colonoscopic images from three tertiary hospitals in China. We retrieved colonoscopic images from Medical Imaging Database as training set at TCH between August 2011 and March 2019. We used images from patients who received colonoscopic examination between April 2019 and May 2019 at TCH as internal test sets, January 2018 and February 2019 at TFCH as first external test set, and January 2018 and December 2018 at TGH as the second external test set. All images and pathological examination reports were deidentified before they were transferred to investigators. All CRC patients and 56.1% (5050/9003) of controls in the training set and all patients in three test sets underwent surgical resection or endoscopy-guided biopsy, therefore, they had pathological examination as gold standard to diagnose CRC. The training set and test sets were obtained from real-world colonoscopic imaging cohort. The control group included patients diagnosed with normal mucosa, adenoma, polyps, familial adenomatous polyposis syndrome, inflammatory bowel disease, and chronic mucosal inflammation. The CRC patient group consisted of patients diagnosed with solitary tumor. We excluded other rare malignant diseases such as sarcoma, melanoma, gastrointestinal stromal tumor, lymphoma, and carcinoid tumor. All patients included in this study were 18 years of age or older. Surgically resected CRC tumors were staged according to the 7th edition of TNM staging system stipulated by American Joint Committee on Cancer. Pathological examination was used as gold standard to diagnose CRC.

Pathological examination of surgical or biopsy samples was used to measure the performance of CRCNet in distinguishing between malignant and benign diseases at patient level. Pathologists B.S., L.S., and X.G. retrieved and reviewed pathologic information from the pathologic system. Consensual interpretation of images from CRC patients by five endoscopists in each test set was used to measure the performance of CRCNet in differentiating malignancy and benignity at the image level. Each endoscopist has at least 6 years of working experience. All these five endoscopists have ≥1000 colonscopic examinations. The optical diagnosis of these five endoscopists were estimated to range from 85 to 95% based on their self-reported performance. For the two external TFCH and TGH test sets, all CRC patients and a random subset of 200 controls were selected and interpreted by endoscopists. For the internal TCH test set, all CRC patients and controls were interpreted by endoscopists. These five endoscopists were asked to classify each selected patient as CRC or control based on their clinical experience.

This study was approved by the Institutional Review Board (IRB) of Tianjin Medical University Cancer Institute and Hospital and conducted in accordance with Declaration of Helsinki. Informed consent from CRC patients and noncancerous controls was exempted by the IRB because of the retrospective nature of this study.

### Curation of colonoscopic images

All images obtained from imaging databases at three hospitals were in jpeg format. All images were generated by high definition instruments (Olympus CF-HQ2, PCF-Q260JI, Tokyo, Japan) after standard bowel preparation. The white-light and NBI modalities were used. However, we included only the white-light images in this study. For patients diagnosed with CRC in the training set, five endoscopists were asked to manually review all the images and classify them into benign or malignant images based on their clinical experience. These five endoscopists read images individually and images manifested malignant lesions were included. Images featured by benign characteristics and images from normal mucosa were combined with those from non-CRC patients to serve as negative samples. Correspondingly, images from CRC patients featured by malignant characteristics were used as positive samples. Low quality images such as motion-blurring, blank, out-of-focus, or poor bowel preparation images (*n* = 13,522) were excluded during manual inspection. In the test sets, all images from each patient were used to measure the performance of CRCNet and endoscopists.

### Model development

CRCNet is a dense convolutional network^27^ of 169 layers trained on the colonoscopic images and their corresponding labels (e.g., benignity or malignancy based on pathological report). The dense convolutional network connects each layer to every other layers in a feed-forward manner^27^, which improves information flow and enables new feature exploration. For each layer, it takes all features learned all preceding layers as inputs, and its outputs are fed into all subsequent layers. Such dense connections can alleviate gradient vanishing, improve feature propagation and reuse, and reduce number of parameters^27^. CRCNet consists of densely connected blocks. Each dense block consists of densely connected layers that composed of batch normalization, activation function, and convolution. The first, second, third, and fourth dense block consists of 6, 12, 32, and 32 densely connected layers, respectively. Dense blocks were connected by transition layers that used to adjust the dimension of feature-map size via convolution and pooling. The architecture of this network was provided as Supplementary Fig. 9. We initialized the weights of CRCNet from the same network that has been trained on the ImageNet^28^ data set except the last fully connected layer. The output unit of the last fully connected layer was set to two to match the number of classes in this study and its weight was randomly initialized. We trained CRCNet in an end-to-end fashion with stochastic gradient descent using an initial learning rate of 0.001, momentum of 0.9, weight decay of 0.0001, and a minibatch of 32. The learning rate was decayed by 0.1 after every 20 epochs. CRCNet was trained for 80 iterations. We used focal loss^26^ as the objective function to train CRCNet to mitigate the sparsity of the positive examples (i.e., malignant images) as compared with negative controls (i.e., benign plus normal images). We applied on-the-fly data augmentation during training to virtually increase data diversity observed in the real world. On-the-fly data augmentation employed in this study include random resize and crop, perspective, horizontal flip, rotation, color jittering, and lighting noise^15^. We used a random subset of images, which was not included during training, to calculate the loss of model at the end of each epoch. We selected model with lowest loss as the best model and evaluated its performance on test sets. The whole procedures were provided as Supplementary Data 6. CRCNet was developed with Python (version 3.7.1), PyTorch (version 1.3.0), and torchvision (version 0.5.0).

### Visual explanation

We used Grad-CAM^21^ to derive visual explanation by localizing the image area that most influences the decision made by CRCNet. We also used Guided Grad-CAM^21^ to quantify pixel contribution to the final predicted classification output.

### Calculation of malignancy score

We calculated predicted malignancy score for each individual as the weighted mean of log_10_-transformed predicted probabilities of input images. For a given individual, we denoted *n* as the total number of images available from that individual and ${\mathbf{p}}_{{\mathrm{malignancy}}} = \left[ {{\it{p}}_1,\,{\it{p}}_2,\, \ldots ,\,{\it{p}}_n} \right]$ as the predicted probabilities of these *n* images being classified as malignancy. The predicted malignancy score *θ* for that individual was calculated as

1 $$\theta = - \left[ {{\it{w}}_{\it{1}} \times {\mathrm{log}}_{10}\left( {1 - {\it{p}}_1} \right) + {\it{w}}_{\it{2}} \times {\mathrm{log}}_{10}\left( {1 - {\it{p}}_{\it{2}}} \right) + \cdot \cdot \cdot + {\it{w}}_{\it{n}} \times {\mathrm{log}}_{10}\left( {1 - {\it{p}}_{\it{n}}} \right)} \right]/{\it{n}},$$

where *w*_*i*_ is calculated as ${\it{w}}_{\it{i}} = p_i/\left( {{\it{p}}_1 + p_2 + \ldots + {\it{p}}_{\it{n}}} \right).$

The performance of CRCNet was evaluated on three test sets. We also evaluated the performance of CRCNet in identifying CRC from left-sided (descending and sigmoid colon), right-sided (ascending and transverse colon), and rectal locations. At the patient level, we compared the performance of CRCNet versus five endoscopists on these test sets. In addition, we asked these five endoscopists to manually review the false negatives with low predicted malignancy scores and false positives with high predicted malignancy scores. At the image level, we used consensus interpretation of images from CRC patients in these three test sets by these five endoscopists to measure the classification performance of CRCNet.

### Statistical analysis

We used PR curve and ROC curve to describe the classification ability of CRCNet. The PRC was demonstrated to be more informative than receiver-operating curve on imbalanced datasets^29^. We created the PR curve by plotting recall rate (also known as sensitivity) against precision (also known as positive predictive rate) by varying the predicted probability threshold. The ROC curve was created by plotting recall rate against negative predictive rate (also known as specificity). We used kappa coefficient to measure the inter-rater agreement and agreement between prediction output and pathological examination. The F1 metric is defined as harmonic mean between precision and recall rate, which is calculated as F1 = 2 × precision × recall/(precision + recall). The 95% confidence intervals for sensitivity, specificity, positive predictive rate, and negative predicted rate were calculated by the Clopper–Pearson method^30^. We plotted PR curve and calculated the AUPRC with R package PRROC^31^ (version 1.3.1). We plotted the ROC curve and AUROC with R package pROC (version 1.10.0). We calculated the interendoscopists agreement rate and Fleiss’ kappa using R package irr (version 0.84). Statistical analysis was conducted with R software (version 3.4.3). We used AUPRC as the primary outcome to measure performance of CRCNet. We used precision, recall rate and F1 score when comparing the classification ability of CRCNet with endoscopists.

### Reporting summary

Further information on research design is available in the Nature Research Reporting Summary linked to this article.

## Supplementary information

Supplementary Information

Supplementary Data 1

Supplementary Data 2

Supplementary Data 3

Supplementary Data 4

Supplementary Data 5

Supplementary Data 6

Description of Additional Supplementary Files

Reporting Summary

## Data Availability

The authors declare that the data supporting the findings of this study are available within the paper and its supplementary information files. Restrictions apply to the availability of the training and test sets, which were used with permission for the current study, and so are not publicly available. Databases used include Colonoscopic Imaging Databases and ImageNet (http://www.image-net.org/).

## Code availability

The code used to train and evaluate the model is available on GitHub (https://github.com/lixiangchun/AIplus/tree/master/CRCNet).
