# Supplementary material for: Diagnostic evaluation of a deep learning model for optical diagnosis of colorectal cancer
Source: Nat Commun. 2020 Jun 11;11:2961. doi: 10.1038/s41467-020-16777-6 (PMC7289893; doi:10.1038/s41467-020-16777-6)
Supplement: Supplementary file 1 — Supplementary Information [file 41467_2020_16777_MOESM1_ESM.pdf]

Diagnostic evaluation of a deep learning model for optical diagnosis of colorectal cancer

Zhou *et al.*

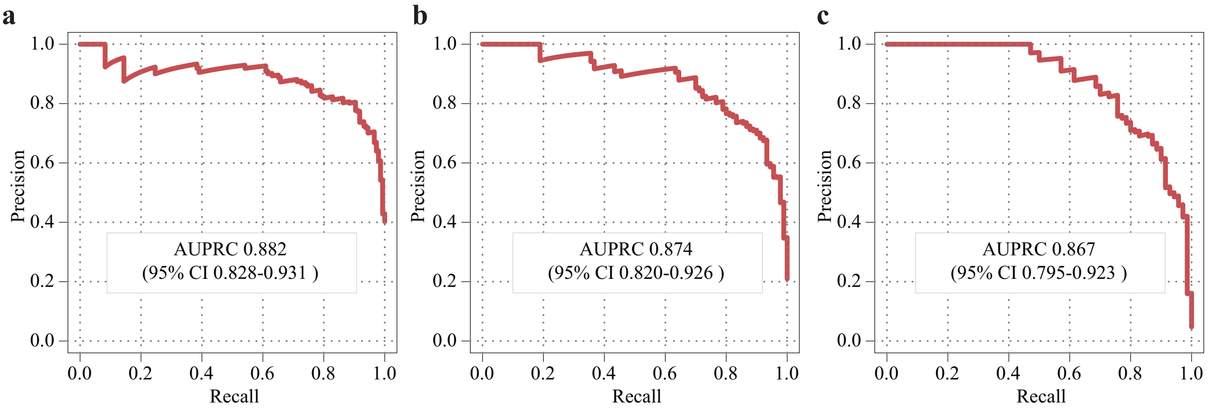

Supplementary Figure 1. The precision-recall curves of CRCNet in identifying CRC patients on TCH (a), TFCH (b) and TGH (c) test sets.

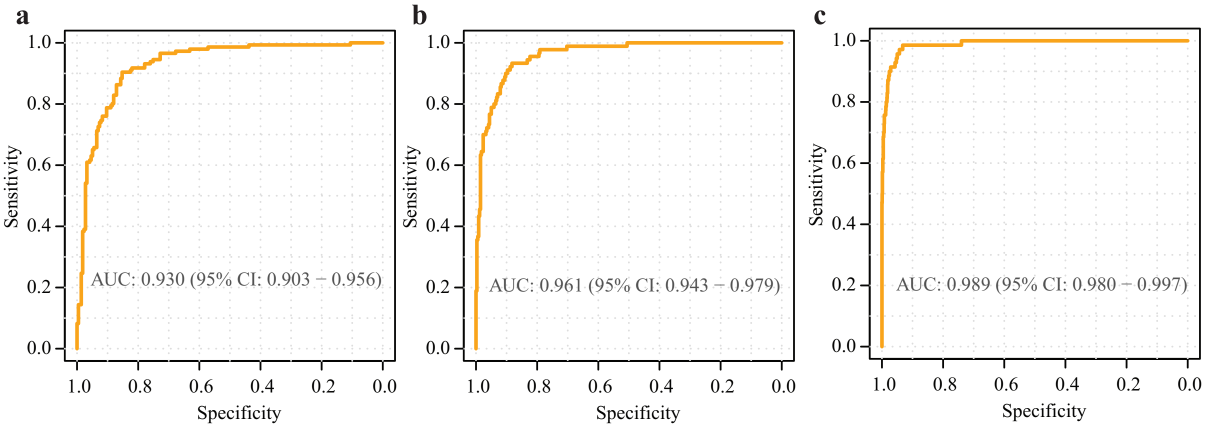

Supplementary Figure 2. The receiver operating characteristic curves of CRCNet in identifying CRC patients on TCH (a), TFCH (b) and TGH (c) test sets.

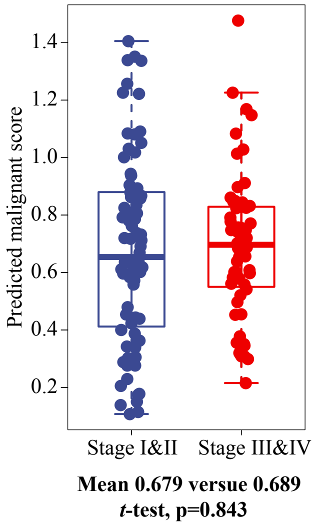

Supplementary Figure 3. Distribution of predicted malignancy scores in CRC patients stratified by TNM stage. Two-sided t-test was used to assess the statistical difference for CRC patients at stage I&II (n=76) and III&IV (n=60). Data are

represented as boxplots where the middle line, lower and upper hinges correspond to median, the first and third quantiles, respectively. The upper whisker extends from the hinge to the largest value no further than 1.5 × IQR from the hinge (where IQR is the inter-quartile range) and the lower whisker extends from the hinge to the smallest value at most 1.5 × IQR of the hinge, while data beyond the end of the whiskers are outlying points that are plotted individually.

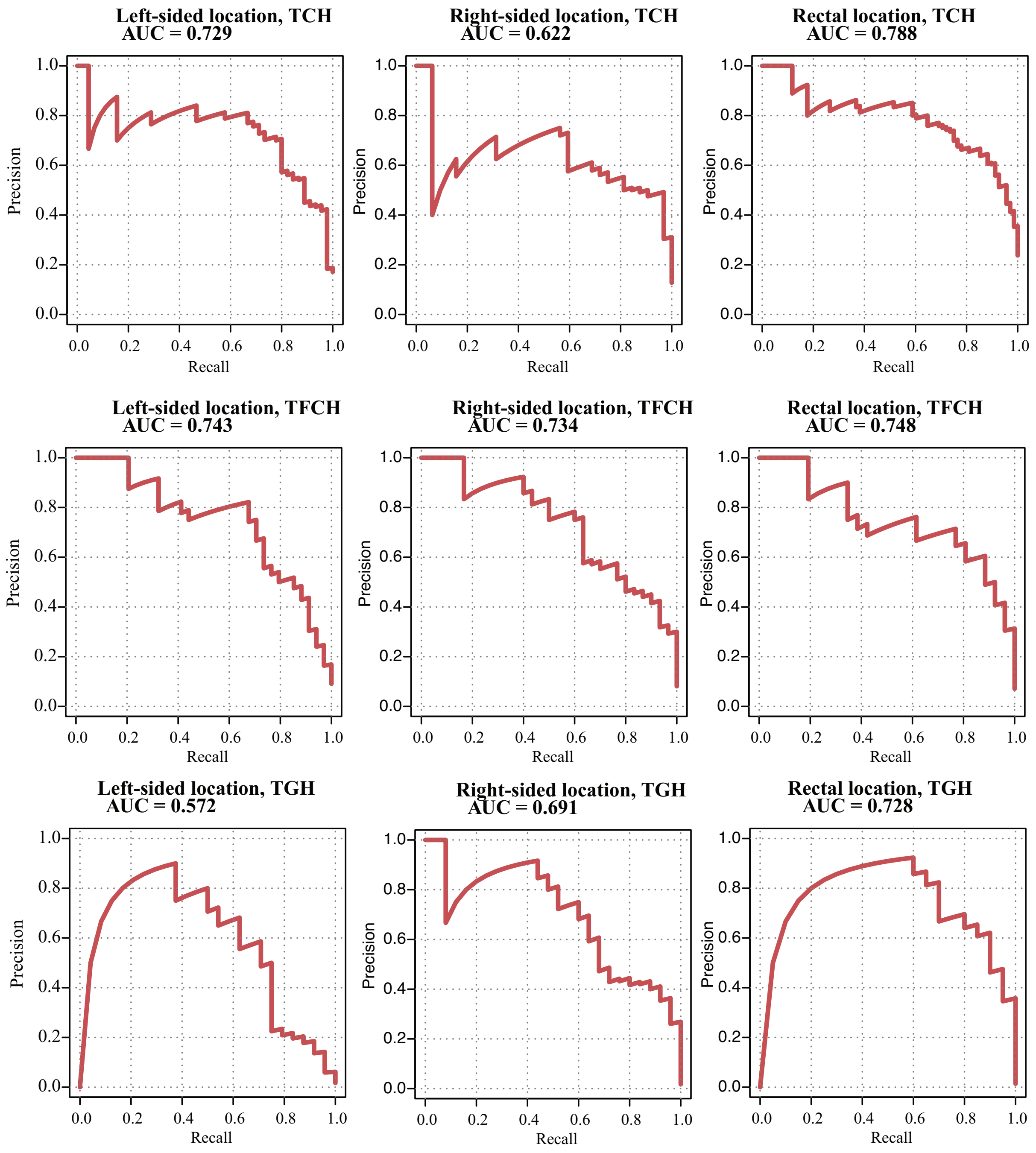

Supplementary Figure 4. The precision-recall curves of CRCNet in identifying CRC patients stratified by left-sided, right-sided and rectal tumor locations.

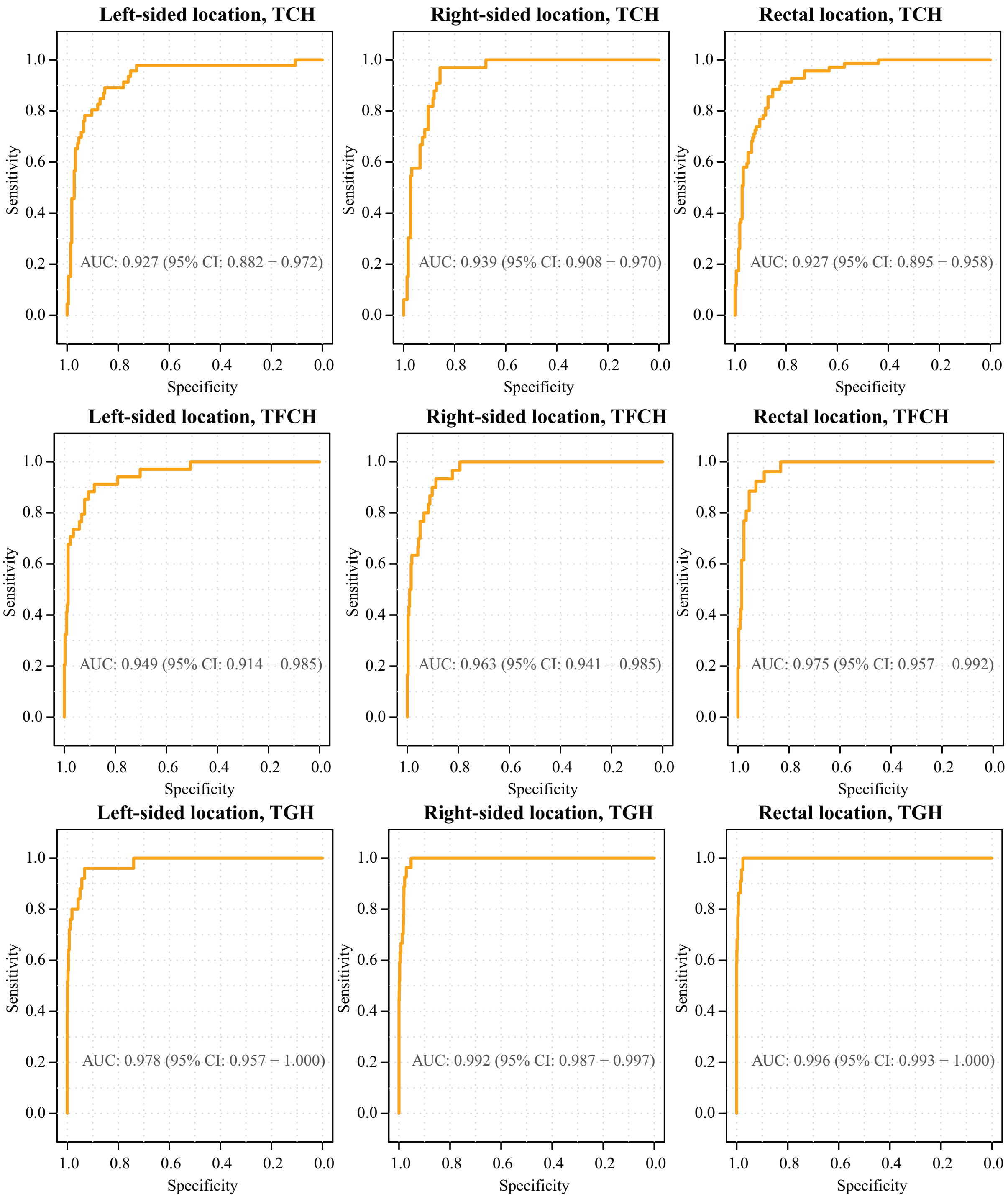

Supplementary Figure 5. The receiver operating characteristic curves of CRCNet in identifying CRC patients stratified by left-sided, right-sided and rectal tumor locations.

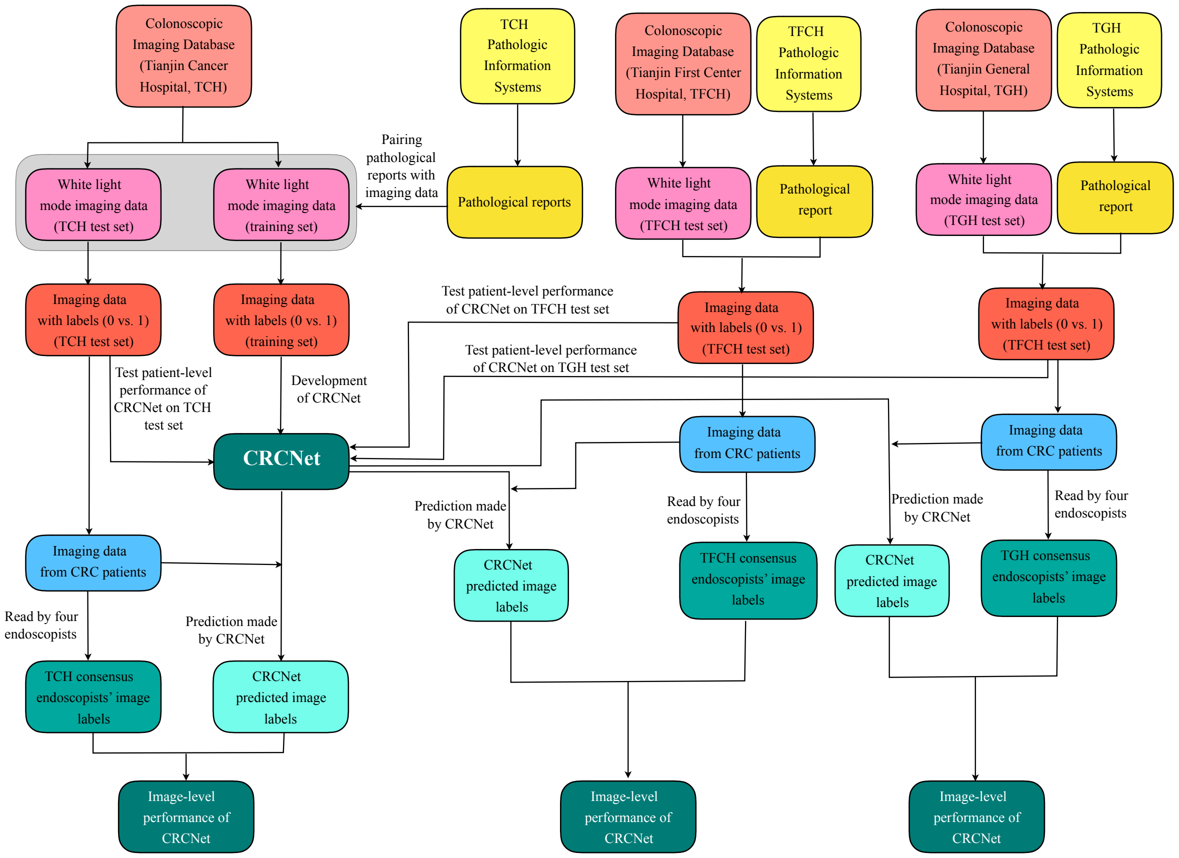

Supplementary Figure 6. A flowchart depicting the whole methods.

Supplementary Table 1. The statistics of benign diseases in training set and test sets.

	TCH training set (n = 5050)	TCH test set (n = 217)	TFCH test set (n = 340)	TGH test set (n = 1399)
Adenoma	2762 (54.7%)	98 (45.2%)	116 (34.1%)	621 (44.4%)
Hyperplastic polyps	1190 (23.6%)	41 (18.9%)	99 (29.1%)	88 (6.3%)
Sessile serrated adenomas/polyps	227 (4.5%)	5 (2.3%)	6 (1.8%)	50 (3.6%)
Inflammatory bowel disease	162 (3.2%)	8 (3.7%)	18 (5.3%)	20 (1.4%)
Chronic mucosal inflammation	709 (14%)	65 (30%)	101 (29.7%)	620 (44.3%)

The percentage of benign diseases were reported for non-CRC patients who received biopsy or surgical resection. The number of benign diseases (n) for each data set were added.

Supplementary Table 2. Detailed classification metrics for each endoscopist across three test sets.

Performance metrics	The performance of five endoscopists and CRCNet in randomly selected patients from three test sets					
	Tianjin Cancer Hospital (n=363)					
	Endoscopist 1	Endoscopist 2	Endoscopist 3	Endoscopist 4	Endoscopist 5	CRCNet
Accuracy (95% CI)	0.694 (0.644 - 0.741)	0.792 (0.747 - 0.833)	0.931 (0.900 - 0.955)	0.871 (0.832 - 0.903)	0.824 (0.781 - 0.861)	0.873 (0.835 - 0.906)
Recall rate (95% CI)	0.932 (0.878 - 0.967)	0.849 (0.781 - 0.903)	0.918 (0.861 - 0.957)	0.801 (0.727 - 0.863)	0.692 (0.610 - 0.765)	0.904 (0.844 - 0.947)
Specificity (95% CI)	0.535 (0.466 - 0.602)	0.753 (0.690 - 0.810)	0.940 (0.900 - 0.968)	0.917 (0.872 - 0.950)	0.912 (0.867 - 0.946)	0.853 (0.798 - 0.897)
Precision (95% CI)	0.574 (0.508 - 0.638)	0.701 (0.627 - 0.767)	0.912 (0.854 - 0.952)	0.867 (0.797 - 0.919)	0.842 (0.764 - 0.902)	0.805 (0.736 - 0.863)
Negative predicted value (95% CI)	0.921 (0.859 - 0.961)	0.880 (0.825 - 0.924)	0.944 (0.905 - 0.971)	0.873 (0.822 - 0.913)	0.815 (0.760 - 0.862)	0.930 (0.885 - 0.961)
Kappa[§]	0.423	0.583	0.857	0.727	0.622	0.742
F_1[†]	0.710	0.768	0.915	0.833	0.759	0.852
Performance metrics	Tianjin First Central Hospital (n=290)					
	Endoscopist 1	Endoscopist 2	Endoscopist 3	Endoscopist 4	Endoscopist 5	CRCNet
Accuracy (95% CI)	0.852 (0.806 - 0.891)	0.928 (0.891 - 0.955)	0.890 (0.848 - 0.923)	0.921 (0.883 - 0.949)	0.945 (0.912 - 0.968)	0.903 (0.863 - 0.935)
Recall rate (95% CI)	0.789 (0.690 - 0.868)	0.867 (0.779 - 0.929)	0.867 (0.779 - 0.929)	0.922 (0.846 - 0.968)	0.933 (0.861 - 0.975)	0.933 (0.861 - 0.975)
Specificity (95% CI)	0.880 (0.827 - 0.922)	0.955 (0.916 - 0.979)	0.900 (0.850 - 0.938)	0.920 (0.873 - 0.954)	0.950 (0.910 - 0.976)	0.890 (0.838 - 0.930)
Precision (95% CI)	0.747 (0.648 - 0.831)	0.897 (0.813 - 0.952)	0.796 (0.703 - 0.871)	0.838 (0.751 - 0.905)	0.894 (0.813 - 0.948)	0.792 (0.703 - 0.865)
Negative predicted value (95% CI)	0.903 (0.852 - 0.940)	0.941 (0.899 - 0.969)	0.938 (0.893 - 0.967)	0.963 (0.926 - 0.985)	0.969 (0.935 - 0.989)	0.967 (0.930 - 0.988)
Kappa[§]	0.66	0.829	0.748	0.820	0.873	0.785
F_1[†]	0.768	0.881	0.830	0.878	0.913	0.857
performance metrics	Tianjin General Hospital (n=271)					
	Endoscopist 1	Endoscopist 2	Endoscopist 3	Endoscopist 4	Endoscopist 5	CRCNet
Accuracy (95% CI)	0.908 (0.867 - 0.939)	0.937 (0.901 - 0.963)	0.940 (0.904 - 0.965)	0.930 (0.893 - 0.957)	0.934 (0.897 - 0.960)	0.963 (0.933 - 0.982)
Recall rate (95% CI)	0.857 (0.753 - 0.929)	0.929 (0.841 - 0.976)	0.943 (0.860 - 0.984)	0.900 (0.805 - 0.959)	0.886 (0.787 - 0.949)	0.914 (0.823 - 0.968)
Specificity (95% CI)	0.925 (0.880 - 0.958)	0.940 (0.898 - 0.969)	0.938 (0.895 - 0.968)	0.940 (0.898 - 0.969)	0.950 (0.910 - 0.976)	0.980 (0.950 - 0.995)
Precision (95% CI)	0.800 (0.692 - 0.884)	0.844 (0.744 - 0.917)	0.846 (0.747 - 0.918)	0.840 (0.737 - 0.914)	0.861 (0.759 - 0.931)	0.941 (0.856 - 0.984)
Negative predicted value (95% CI)	0.949 (0.908 - 0.975)	0.974 (0.941 - 0.992)	0.979 (0.946 - 0.994)	0.964 (0.928 - 0.986)	0.960 (0.922 - 0.982)	0.970 (0.937 - 0.989)
Kappa[§]	0.765	0.841	0.850	0.821	0.828	0.903
F_1[†]	0.828	0.884	0.892	0.869	0.873	0.928

§ Measures the agreement between predicted classification with pathological report.

† Harmonic average of the precision and recall rate.

Supplementary Table 3. The classification performance of CRCNet at image-level.

Performance metrics	The performance of CRCNet model at image level		
	Tianjin Cancer Hospital (k=5492)	Tianjin First Central Hospital (k=1705)	Tianjin General Hospital (k=1143)
Accuracy (95% CI)	0.979 (0.975 - 0.983)	0.969 (0.960 - 0.977)	0.981 (0.971 - 0.988)
Recall rate (95% CI)	0.939 (0.924 - 0.951)	0.954 (0.936 - 0.968)	0.966 (0.943 - 0.981)
Specificity (95% CI)	0.992 (0.989 - 0.995)	0.980 (0.970 - 0.988)	0.989 (0.979 - 0.995)
Precision (95% CI)	0.974 (0.964 - 0.982)	0.974 (0.959 - 0.984)	0.980 (0.961 - 0.991)
Negative predicted value (95% CI)	0.981 (0.976 - 0.984)	0.966 (0.952 - 0.976)	0.981 (0.968 - 0.990)
Kappa[§]	0.942	0.936	0.958
F_1[†]	0.956	0.963	0.973

[§] Measures the agreement between predicted classification with pathological report.

[†] Harmonic average of the precision and recall rate.

Supplementary Table 4. The accuracy of automatic lesion detection by CRCNet as assessed by endoscopists.

Accuracy	The accuracy of automatic lesion detection by CRCNet as assessed by endoscopists				
	Endoscopist 1	Endoscopist 2	Endoscopist 3	Endoscopist 4	Endoscopist 5
Covered ≥50%	98.80%	99.60%	98.00%	98.80%	98.40%
Covered ≥90%	93.70%	92.50%	91.80%	96.50%	96.90%
