# Supplementary figures and images for: Diagnostic evaluation of a deep learning model for optical diagnosis of colorectal cancer (part 2 of 5)
Source: Nat Commun. 2020 Jun 11;11:2961. doi: 10.1038/s41467-020-16777-6 (PMC7289893; doi:10.1038/s41467-020-16777-6)

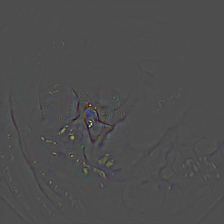

Supplement: Supplementary file 2 — Supplementary Data 1 [file 41467_2020_16777_MOESM2_ESM.gz › SupplementaryData1.36fn/20/IMG_01.0000000020799.0009.15024800202.jpg_benign_ggcam_densenet169_finetune.png]

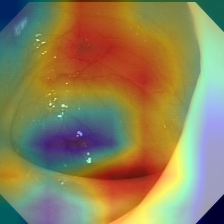

Supplement: Supplementary file 2 — Supplementary Data 1 [file 41467_2020_16777_MOESM2_ESM.gz › SupplementaryData1.36fn/20/IMG_01.0000000020799.0018.15043100932.jpg_benign_gcam_densenet169_finetune.png]

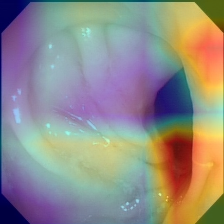

Supplement: Supplementary file 2 — Supplementary Data 1 [file 41467_2020_16777_MOESM2_ESM.gz › SupplementaryData1.36fn/20/IMG_01.0000000020799.0033.15063200866.jpg_benign_gcam_densenet169_finetune.png]

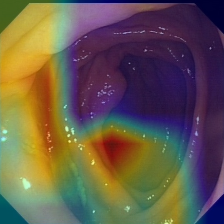

Supplement: Supplementary file 2 — Supplementary Data 1 [file 41467_2020_16777_MOESM2_ESM.gz › SupplementaryData1.36fn/20/IMG_01.0000000020799.0004.15015800915.jpg_benign_gcam_densenet169_finetune.png]

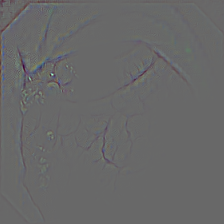

Supplement: Supplementary file 2 — Supplementary Data 1 [file 41467_2020_16777_MOESM2_ESM.gz › SupplementaryData1.36fn/20/IMG_01.0000000020799.0025.15053100324.jpg_benign_ggcam_densenet169_finetune.png]

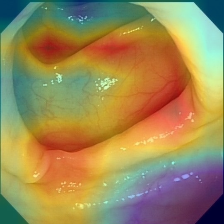

Supplement: Supplementary file 2 — Supplementary Data 1 [file 41467_2020_16777_MOESM2_ESM.gz › SupplementaryData1.36fn/20/IMG_01.0000000020799.0015.15032800336.jpg_benign_gcam_densenet169_finetune.png]

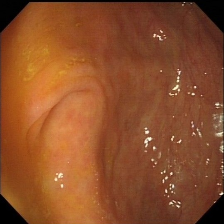

Supplement: Supplementary file 2 — Supplementary Data 1 [file 41467_2020_16777_MOESM2_ESM.gz › SupplementaryData1.36fn/20/IMG_01.0000000020799.0002.15013600959.jpg_benign_gcam_densenet169_finetune.png_raw_image.png]

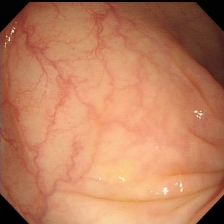

Supplement: Supplementary file 2 — Supplementary Data 1 [file 41467_2020_16777_MOESM2_ESM.gz › SupplementaryData1.36fn/20/IMG_01.0000000020799.0023.15051400636.jpg_benign_gcam_densenet169_finetune.png_raw_image.png]

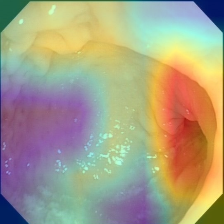

Supplement: Supplementary file 2 — Supplementary Data 1 [file 41467_2020_16777_MOESM2_ESM.gz › SupplementaryData1.36fn/20/IMG_01.0000000020799.0035.15065800059.jpg_benign_gcam_densenet169_finetune.png]

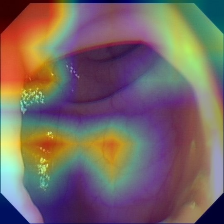

Supplement: Supplementary file 2 — Supplementary Data 1 [file 41467_2020_16777_MOESM2_ESM.gz › SupplementaryData1.36fn/20/IMG_01.0000000020799.0025.15053100324.jpg_benign_gcam_densenet169_finetune.png]

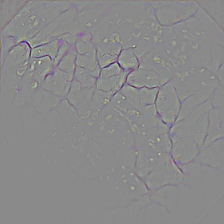

Supplement: Supplementary file 2 — Supplementary Data 1 [file 41467_2020_16777_MOESM2_ESM.gz › SupplementaryData1.36fn/20/IMG_01.0000000020799.0028.15055800619.jpg_benign_ggcam_densenet169_finetune.png]

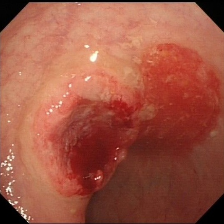

Supplement: Supplementary file 2 — Supplementary Data 1 [file 41467_2020_16777_MOESM2_ESM.gz › SupplementaryData1.36fn/20/IMG_01.0000000020799.0043.15131200457.jpg_benign_gcam_densenet169_finetune.png_raw_image.png]

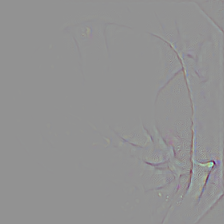

Supplement: Supplementary file 2 — Supplementary Data 1 [file 41467_2020_16777_MOESM2_ESM.gz › SupplementaryData1.36fn/20/IMG_01.0000000020799.0033.15063200866.jpg_benign_ggcam_densenet169_finetune.png]

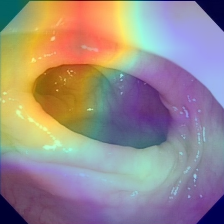

Supplement: Supplementary file 2 — Supplementary Data 1 [file 41467_2020_16777_MOESM2_ESM.gz › SupplementaryData1.36fn/20/IMG_01.0000000020799.0027.15055400244.jpg_benign_gcam_densenet169_finetune.png]

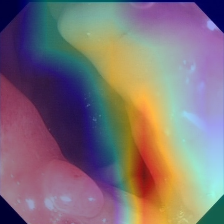

Supplement: Supplementary file 2 — Supplementary Data 1 [file 41467_2020_16777_MOESM2_ESM.gz › SupplementaryData1.36fn/20/IMG_01.0000000020799.0036.15070600289.jpg_benign_gcam_densenet169_finetune.png]

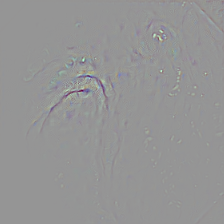

Supplement: Supplementary file 2 — Supplementary Data 1 [file 41467_2020_16777_MOESM2_ESM.gz › SupplementaryData1.36fn/20/IMG_01.0000000020799.0002.15013600959.jpg_benign_ggcam_densenet169_finetune.png]

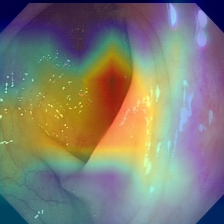

Supplement: Supplementary file 2 — Supplementary Data 1 [file 41467_2020_16777_MOESM2_ESM.gz › SupplementaryData1.36fn/20/IMG_01.0000000020799.0020.15043900934.jpg_benign_gcam_densenet169_finetune.png]

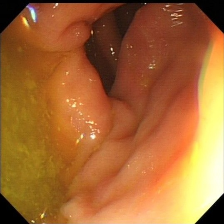

Supplement: Supplementary file 2 — Supplementary Data 1 [file 41467_2020_16777_MOESM2_ESM.gz › SupplementaryData1.36fn/20/IMG_01.0000000020799.0008.15022000660.jpg_benign_gcam_densenet169_finetune.png_raw_image.png]

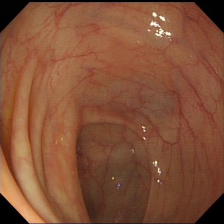

Supplement: Supplementary file 2 — Supplementary Data 1 [file 41467_2020_16777_MOESM2_ESM.gz › SupplementaryData1.36fn/20/IMG_01.0000000020799.0019.15043400491.jpg_benign_gcam_densenet169_finetune.png_raw_image.png]

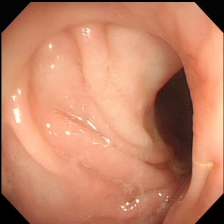

Supplement: Supplementary file 2 — Supplementary Data 1 [file 41467_2020_16777_MOESM2_ESM.gz › SupplementaryData1.36fn/20/IMG_01.0000000020799.0033.15063200866.jpg_benign_gcam_densenet169_finetune.png_raw_image.png]

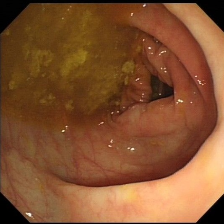

Supplement: Supplementary file 2 — Supplementary Data 1 [file 41467_2020_16777_MOESM2_ESM.gz › SupplementaryData1.36fn/20/IMG_01.0000000020799.0011.15025300077.jpg_benign_gcam_densenet169_finetune.png_raw_image.png]

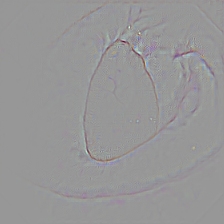

Supplement: Supplementary file 2 — Supplementary Data 1 [file 41467_2020_16777_MOESM2_ESM.gz › SupplementaryData1.36fn/20/IMG_01.0000000020799.0031.15061100696.jpg_benign_ggcam_densenet169_finetune.png]

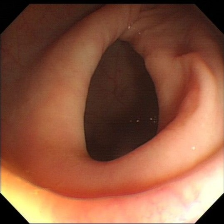

Supplement: Supplementary file 2 — Supplementary Data 1 [file 41467_2020_16777_MOESM2_ESM.gz › SupplementaryData1.36fn/20/IMG_01.0000000020799.0031.15061100696.jpg_benign_gcam_densenet169_finetune.png_raw_image.png]

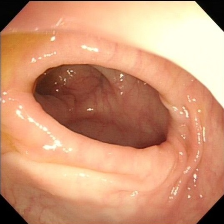

Supplement: Supplementary file 2 — Supplementary Data 1 [file 41467_2020_16777_MOESM2_ESM.gz › SupplementaryData1.36fn/20/IMG_01.0000000020799.0027.15055400244.jpg_benign_gcam_densenet169_finetune.png_raw_image.png]

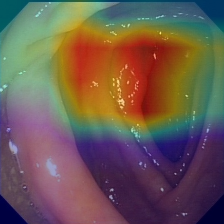

Supplement: Supplementary file 2 — Supplementary Data 1 [file 41467_2020_16777_MOESM2_ESM.gz › SupplementaryData1.36fn/20/IMG_01.0000000020799.0006.15020800927.jpg_benign_gcam_densenet169_finetune.png]

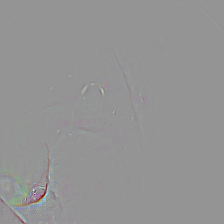

Supplement: Supplementary file 2 — Supplementary Data 1 [file 41467_2020_16777_MOESM2_ESM.gz › SupplementaryData1.36fn/20/IMG_01.0000000020799.0040.15074900554.jpg_benign_ggcam_densenet169_finetune.png]

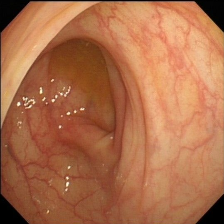

Supplement: Supplementary file 2 — Supplementary Data 1 [file 41467_2020_16777_MOESM2_ESM.gz › SupplementaryData1.36fn/20/IMG_01.0000000020799.0022.15045800311.jpg_benign_gcam_densenet169_finetune.png_raw_image.png]

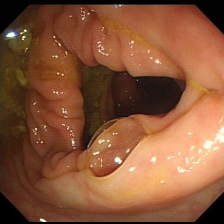

Supplement: Supplementary file 2 — Supplementary Data 1 [file 41467_2020_16777_MOESM2_ESM.gz › SupplementaryData1.36fn/20/IMG_01.0000000020799.0010.15025100186.jpg_benign_gcam_densenet169_finetune.png_raw_image.png]

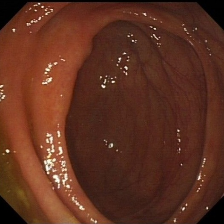

Supplement: Supplementary file 2 — Supplementary Data 1 [file 41467_2020_16777_MOESM2_ESM.gz › SupplementaryData1.36fn/20/IMG_01.0000000020799.0003.15015500083.jpg_benign_gcam_densenet169_finetune.png_raw_image.png]

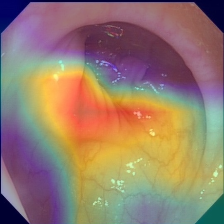

Supplement: Supplementary file 2 — Supplementary Data 1 [file 41467_2020_16777_MOESM2_ESM.gz › SupplementaryData1.36fn/20/IMG_01.0000000020799.0026.15054200726.jpg_benign_gcam_densenet169_finetune.png]

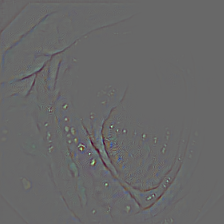

Supplement: Supplementary file 2 — Supplementary Data 1 [file 41467_2020_16777_MOESM2_ESM.gz › SupplementaryData1.36fn/20/IMG_01.0000000020799.0004.15015800915.jpg_benign_ggcam_densenet169_finetune.png]

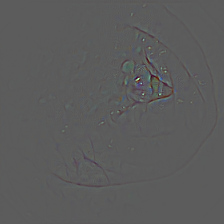

Supplement: Supplementary file 2 — Supplementary Data 1 [file 41467_2020_16777_MOESM2_ESM.gz › SupplementaryData1.36fn/20/IMG_01.0000000020799.0011.15025300077.jpg_benign_ggcam_densenet169_finetune.png]

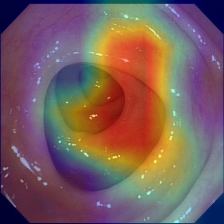

Supplement: Supplementary file 2 — Supplementary Data 1 [file 41467_2020_16777_MOESM2_ESM.gz › SupplementaryData1.36fn/17/IMG_01.0000000018312.0033.14481300833.jpg_benign_gcam_densenet169_finetune.png]

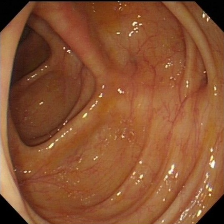

Supplement: Supplementary file 2 — Supplementary Data 1 [file 41467_2020_16777_MOESM2_ESM.gz › SupplementaryData1.36fn/17/IMG_01.0000000018312.0013.14455300565.jpg_benign_gcam_densenet169_finetune.png_raw_image.png]

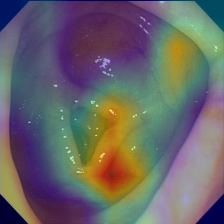

Supplement: Supplementary file 2 — Supplementary Data 1 [file 41467_2020_16777_MOESM2_ESM.gz › SupplementaryData1.36fn/17/IMG_01.0000000018312.0022.14464300942.jpg_benign_gcam_densenet169_finetune.png]

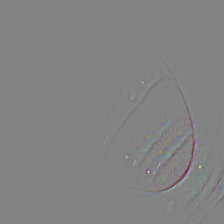

Supplement: Supplementary file 2 — Supplementary Data 1 [file 41467_2020_16777_MOESM2_ESM.gz › SupplementaryData1.36fn/17/IMG_01.0000000018312.0011.14453500600.jpg_benign_ggcam_densenet169_finetune.png]

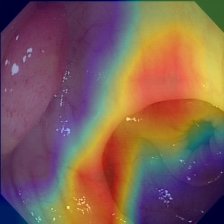

Supplement: Supplementary file 2 — Supplementary Data 1 [file 41467_2020_16777_MOESM2_ESM.gz › SupplementaryData1.36fn/17/IMG_01.0000000018312.0044.14495400097.jpg_benign_gcam_densenet169_finetune.png]

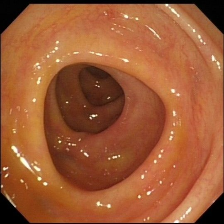

Supplement: Supplementary file 2 — Supplementary Data 1 [file 41467_2020_16777_MOESM2_ESM.gz › SupplementaryData1.36fn/17/IMG_01.0000000018312.0033.14481300833.jpg_benign_gcam_densenet169_finetune.png_raw_image.png]

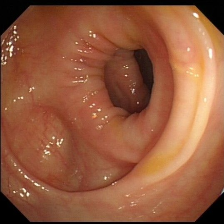

Supplement: Supplementary file 2 — Supplementary Data 1 [file 41467_2020_16777_MOESM2_ESM.gz › SupplementaryData1.36fn/17/IMG_01.0000000018312.0043.14494000156.jpg_benign_gcam_densenet169_finetune.png_raw_image.png]

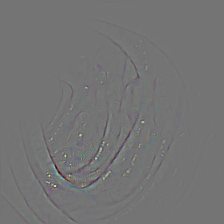

Supplement: Supplementary file 2 — Supplementary Data 1 [file 41467_2020_16777_MOESM2_ESM.gz › SupplementaryData1.36fn/17/IMG_01.0000000018312.0014.14455500674.jpg_benign_ggcam_densenet169_finetune.png]

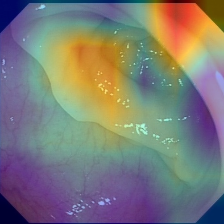

Supplement: Supplementary file 2 — Supplementary Data 1 [file 41467_2020_16777_MOESM2_ESM.gz › SupplementaryData1.36fn/17/IMG_01.0000000018312.0047.14501500333.jpg_benign_gcam_densenet169_finetune.png]

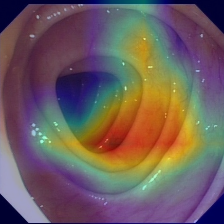

Supplement: Supplementary file 2 — Supplementary Data 1 [file 41467_2020_16777_MOESM2_ESM.gz › SupplementaryData1.36fn/17/IMG_01.0000000018312.0018.14461300069.jpg_benign_gcam_densenet169_finetune.png]

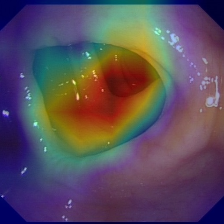

Supplement: Supplementary file 2 — Supplementary Data 1 [file 41467_2020_16777_MOESM2_ESM.gz › SupplementaryData1.36fn/17/IMG_01.0000000018312.0010.14452400172.jpg_benign_gcam_densenet169_finetune.png]

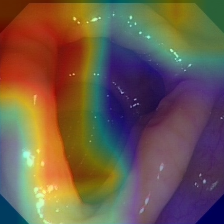

Supplement: Supplementary file 2 — Supplementary Data 1 [file 41467_2020_16777_MOESM2_ESM.gz › SupplementaryData1.36fn/17/IMG_01.0000000018312.0021.14464000753.jpg_benign_gcam_densenet169_finetune.png]

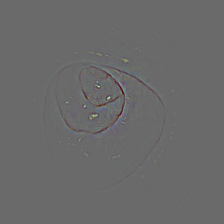

Supplement: Supplementary file 2 — Supplementary Data 1 [file 41467_2020_16777_MOESM2_ESM.gz › SupplementaryData1.36fn/17/IMG_01.0000000018312.0033.14481300833.jpg_benign_ggcam_densenet169_finetune.png]

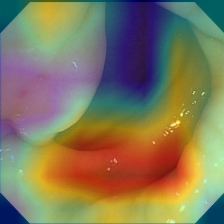

Supplement: Supplementary file 2 — Supplementary Data 1 [file 41467_2020_16777_MOESM2_ESM.gz › SupplementaryData1.36fn/17/IMG_01.0000000018312.0005.14433700914.jpg_benign_gcam_densenet169_finetune.png]

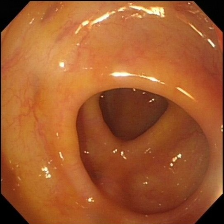

Supplement: Supplementary file 2 — Supplementary Data 1 [file 41467_2020_16777_MOESM2_ESM.gz › SupplementaryData1.36fn/17/IMG_01.0000000018312.0032.14480700479.jpg_benign_gcam_densenet169_finetune.png_raw_image.png]

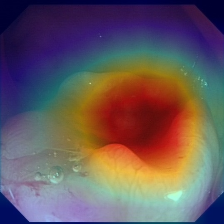

Supplement: Supplementary file 2 — Supplementary Data 1 [file 41467_2020_16777_MOESM2_ESM.gz › SupplementaryData1.36fn/17/IMG_01.0000000018312.0001.14303700029.jpg_malignant_gcam_densenet169_finetune.png]

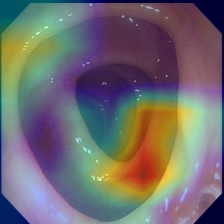

Supplement: Supplementary file 2 — Supplementary Data 1 [file 41467_2020_16777_MOESM2_ESM.gz › SupplementaryData1.36fn/17/IMG_01.0000000018312.0025.14471200495.jpg_benign_gcam_densenet169_finetune.png]

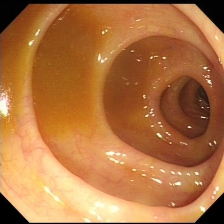

Supplement: Supplementary file 2 — Supplementary Data 1 [file 41467_2020_16777_MOESM2_ESM.gz › SupplementaryData1.36fn/17/IMG_01.0000000018312.0039.14491900224.jpg_benign_gcam_densenet169_finetune.png_raw_image.png]

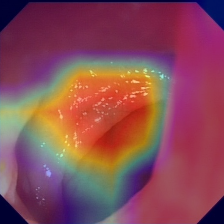

Supplement: Supplementary file 2 — Supplementary Data 1 [file 41467_2020_16777_MOESM2_ESM.gz › SupplementaryData1.36fn/17/IMG_01.0000000018312.0007.14450800802.jpg_benign_gcam_densenet169_finetune.png]

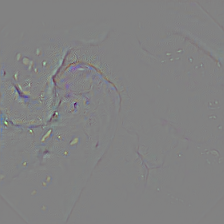

Supplement: Supplementary file 2 — Supplementary Data 1 [file 41467_2020_16777_MOESM2_ESM.gz › SupplementaryData1.36fn/17/IMG_01.0000000018312.0003.14352500393.jpg_benign_ggcam_densenet169_finetune.png]

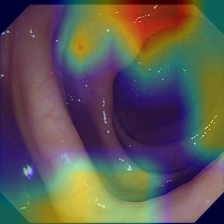

Supplement: Supplementary file 2 — Supplementary Data 1 [file 41467_2020_16777_MOESM2_ESM.gz › SupplementaryData1.36fn/17/IMG_01.0000000018312.0009.14452100017.jpg_benign_gcam_densenet169_finetune.png]

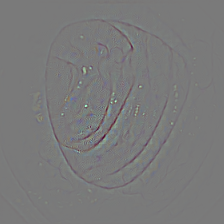

Supplement: Supplementary file 2 — Supplementary Data 1 [file 41467_2020_16777_MOESM2_ESM.gz › SupplementaryData1.36fn/17/IMG_01.0000000018312.0016.14460800556.jpg_benign_ggcam_densenet169_finetune.png]

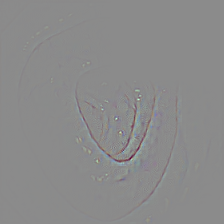

Supplement: Supplementary file 2 — Supplementary Data 1 [file 41467_2020_16777_MOESM2_ESM.gz › SupplementaryData1.36fn/17/IMG_01.0000000018312.0025.14471200495.jpg_benign_ggcam_densenet169_finetune.png]

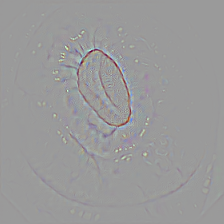

Supplement: Supplementary file 2 — Supplementary Data 1 [file 41467_2020_16777_MOESM2_ESM.gz › SupplementaryData1.36fn/17/IMG_01.0000000018312.0040.14492300214.jpg_benign_ggcam_densenet169_finetune.png]

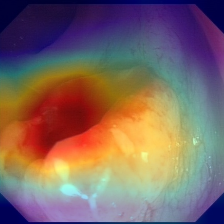

Supplement: Supplementary file 2 — Supplementary Data 1 [file 41467_2020_16777_MOESM2_ESM.gz › SupplementaryData1.36fn/17/IMG_01.0000000018312.0053.14523800628.jpg_malignant_gcam_densenet169_finetune.png]

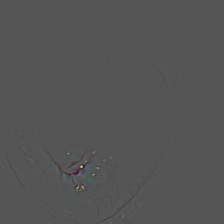

Supplement: Supplementary file 2 — Supplementary Data 1 [file 41467_2020_16777_MOESM2_ESM.gz › SupplementaryData1.36fn/17/IMG_01.0000000018312.0020.14463500785.jpg_benign_ggcam_densenet169_finetune.png]

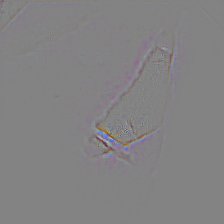

Supplement: Supplementary file 2 — Supplementary Data 1 [file 41467_2020_16777_MOESM2_ESM.gz › SupplementaryData1.36fn/17/IMG_01.0000000018312.0006.14450400955.jpg_benign_ggcam_densenet169_finetune.png]

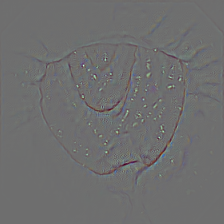

Supplement: Supplementary file 2 — Supplementary Data 1 [file 41467_2020_16777_MOESM2_ESM.gz › SupplementaryData1.36fn/17/IMG_01.0000000018312.0029.14473600602.jpg_benign_ggcam_densenet169_finetune.png]

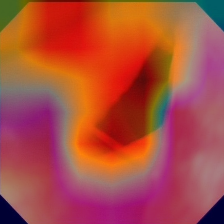

Supplement: Supplementary file 2 — Supplementary Data 1 [file 41467_2020_16777_MOESM2_ESM.gz › SupplementaryData1.36fn/17/IMG_01.0000000018312.0006.14450400955.jpg_benign_gcam_densenet169_finetune.png]

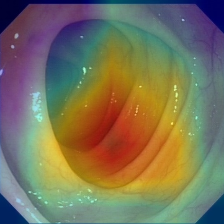

Supplement: Supplementary file 2 — Supplementary Data 1 [file 41467_2020_16777_MOESM2_ESM.gz › SupplementaryData1.36fn/17/IMG_01.0000000018312.0016.14460800556.jpg_benign_gcam_densenet169_finetune.png]

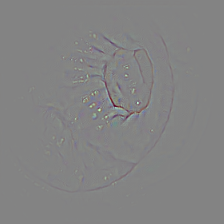

Supplement: Supplementary file 2 — Supplementary Data 1 [file 41467_2020_16777_MOESM2_ESM.gz › SupplementaryData1.36fn/17/IMG_01.0000000018312.0043.14494000156.jpg_benign_ggcam_densenet169_finetune.png]

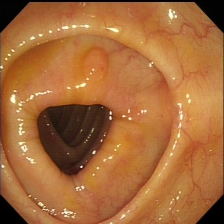

Supplement: Supplementary file 2 — Supplementary Data 1 [file 41467_2020_16777_MOESM2_ESM.gz › SupplementaryData1.36fn/17/IMG_01.0000000018312.0020.14463500785.jpg_benign_gcam_densenet169_finetune.png_raw_image.png]

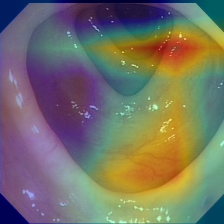

Supplement: Supplementary file 2 — Supplementary Data 1 [file 41467_2020_16777_MOESM2_ESM.gz › SupplementaryData1.36fn/17/IMG_01.0000000018312.0027.14471700180.jpg_benign_gcam_densenet169_finetune.png]

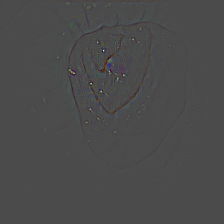

Supplement: Supplementary file 2 — Supplementary Data 1 [file 41467_2020_16777_MOESM2_ESM.gz › SupplementaryData1.36fn/17/IMG_01.0000000018312.0028.14473300541.jpg_benign_ggcam_densenet169_finetune.png]

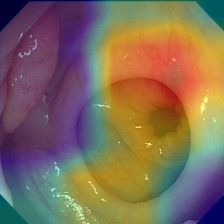

Supplement: Supplementary file 2 — Supplementary Data 1 [file 41467_2020_16777_MOESM2_ESM.gz › SupplementaryData1.36fn/17/IMG_01.0000000018312.0045.14500200386.jpg_benign_gcam_densenet169_finetune.png]

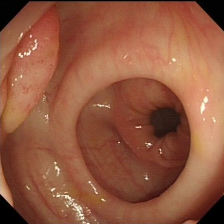

Supplement: Supplementary file 2 — Supplementary Data 1 [file 41467_2020_16777_MOESM2_ESM.gz › SupplementaryData1.36fn/17/IMG_01.0000000018312.0045.14500200386.jpg_benign_gcam_densenet169_finetune.png_raw_image.png]

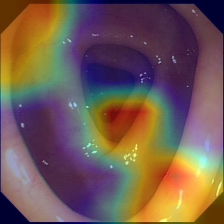

Supplement: Supplementary file 2 — Supplementary Data 1 [file 41467_2020_16777_MOESM2_ESM.gz › SupplementaryData1.36fn/17/IMG_01.0000000018312.0023.14465000733.jpg_benign_gcam_densenet169_finetune.png]

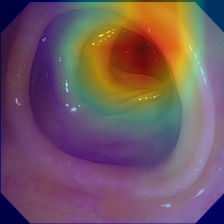

Supplement: Supplementary file 2 — Supplementary Data 1 [file 41467_2020_16777_MOESM2_ESM.gz › SupplementaryData1.36fn/17/IMG_01.0000000018312.0037.14491100994.jpg_benign_gcam_densenet169_finetune.png]

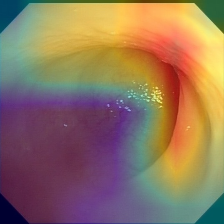

Supplement: Supplementary file 2 — Supplementary Data 1 [file 41467_2020_16777_MOESM2_ESM.gz › SupplementaryData1.36fn/17/IMG_01.0000000018312.0049.14504300269.jpg_benign_gcam_densenet169_finetune.png]

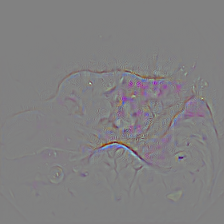

Supplement: Supplementary file 2 — Supplementary Data 1 [file 41467_2020_16777_MOESM2_ESM.gz › SupplementaryData1.36fn/17/IMG_01.0000000018312.0001.14303700029.jpg_malignant_ggcam_densenet169_finetune.png]

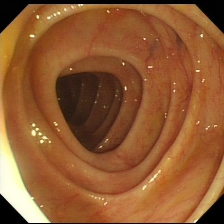

Supplement: Supplementary file 2 — Supplementary Data 1 [file 41467_2020_16777_MOESM2_ESM.gz › SupplementaryData1.36fn/17/IMG_01.0000000018312.0018.14461300069.jpg_benign_gcam_densenet169_finetune.png_raw_image.png]

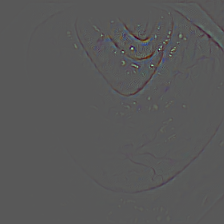

Supplement: Supplementary file 2 — Supplementary Data 1 [file 41467_2020_16777_MOESM2_ESM.gz › SupplementaryData1.36fn/17/IMG_01.0000000018312.0027.14471700180.jpg_benign_ggcam_densenet169_finetune.png]

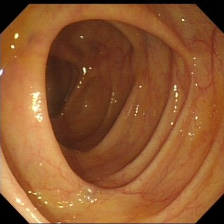

Supplement: Supplementary file 2 — Supplementary Data 1 [file 41467_2020_16777_MOESM2_ESM.gz › SupplementaryData1.36fn/17/IMG_01.0000000018312.0016.14460800556.jpg_benign_gcam_densenet169_finetune.png_raw_image.png]

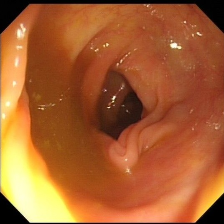

Supplement: Supplementary file 2 — Supplementary Data 1 [file 41467_2020_16777_MOESM2_ESM.gz › SupplementaryData1.36fn/17/IMG_01.0000000018312.0035.14485200873.jpg_benign_gcam_densenet169_finetune.png_raw_image.png]

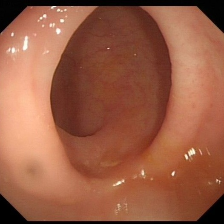

Supplement: Supplementary file 2 — Supplementary Data 1 [file 41467_2020_16777_MOESM2_ESM.gz › SupplementaryData1.36fn/17/IMG_01.0000000018312.0048.14503700001.jpg_benign_gcam_densenet169_finetune.png_raw_image.png]

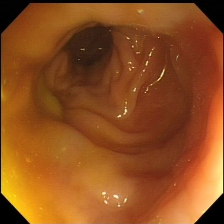

Supplement: Supplementary file 2 — Supplementary Data 1 [file 41467_2020_16777_MOESM2_ESM.gz › SupplementaryData1.36fn/17/IMG_01.0000000018312.0031.14474500373.jpg_benign_gcam_densenet169_finetune.png_raw_image.png]

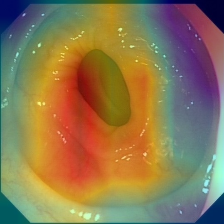

Supplement: Supplementary file 2 — Supplementary Data 1 [file 41467_2020_16777_MOESM2_ESM.gz › SupplementaryData1.36fn/17/IMG_01.0000000018312.0040.14492300214.jpg_benign_gcam_densenet169_finetune.png]

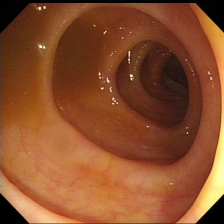

Supplement: Supplementary file 2 — Supplementary Data 1 [file 41467_2020_16777_MOESM2_ESM.gz › SupplementaryData1.36fn/17/IMG_01.0000000018312.0038.14491400687.jpg_benign_gcam_densenet169_finetune.png_raw_image.png]

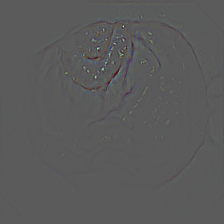

Supplement: Supplementary file 2 — Supplementary Data 1 [file 41467_2020_16777_MOESM2_ESM.gz › SupplementaryData1.36fn/17/IMG_01.0000000018312.0030.14473900380.jpg_benign_ggcam_densenet169_finetune.png]

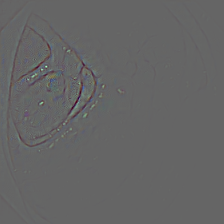

Supplement: Supplementary file 2 — Supplementary Data 1 [file 41467_2020_16777_MOESM2_ESM.gz › SupplementaryData1.36fn/17/IMG_01.0000000018312.0013.14455300565.jpg_benign_ggcam_densenet169_finetune.png]

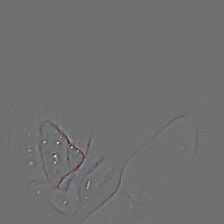

Supplement: Supplementary file 2 — Supplementary Data 1 [file 41467_2020_16777_MOESM2_ESM.gz › SupplementaryData1.36fn/17/IMG_01.0000000018312.0036.14490400383.jpg_benign_ggcam_densenet169_finetune.png]

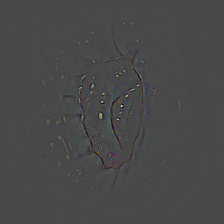

Supplement: Supplementary file 2 — Supplementary Data 1 [file 41467_2020_16777_MOESM2_ESM.gz › SupplementaryData1.36fn/17/IMG_01.0000000018312.0041.14493200832.jpg_benign_ggcam_densenet169_finetune.png]

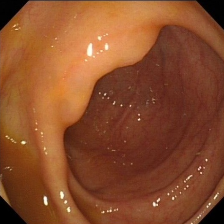

Supplement: Supplementary file 2 — Supplementary Data 1 [file 41467_2020_16777_MOESM2_ESM.gz › SupplementaryData1.36fn/17/IMG_01.0000000018312.0008.14451600927.jpg_benign_gcam_densenet169_finetune.png_raw_image.png]

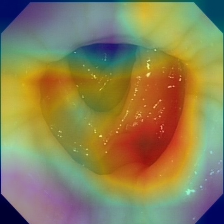

Supplement: Supplementary file 2 — Supplementary Data 1 [file 41467_2020_16777_MOESM2_ESM.gz › SupplementaryData1.36fn/17/IMG_01.0000000018312.0029.14473600602.jpg_benign_gcam_densenet169_finetune.png]

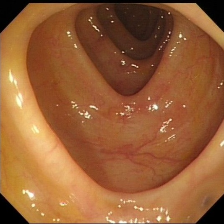

Supplement: Supplementary file 2 — Supplementary Data 1 [file 41467_2020_16777_MOESM2_ESM.gz › SupplementaryData1.36fn/17/IMG_01.0000000018312.0027.14471700180.jpg_benign_gcam_densenet169_finetune.png_raw_image.png]

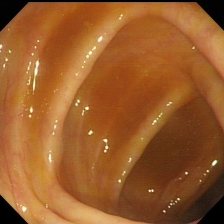

Supplement: Supplementary file 2 — Supplementary Data 1 [file 41467_2020_16777_MOESM2_ESM.gz › SupplementaryData1.36fn/17/IMG_01.0000000018312.0012.14454900852.jpg_benign_gcam_densenet169_finetune.png_raw_image.png]

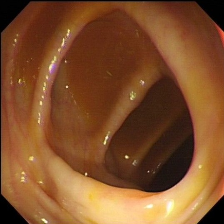

Supplement: Supplementary file 2 — Supplementary Data 1 [file 41467_2020_16777_MOESM2_ESM.gz › SupplementaryData1.36fn/17/IMG_01.0000000018312.0011.14453500600.jpg_benign_gcam_densenet169_finetune.png_raw_image.png]

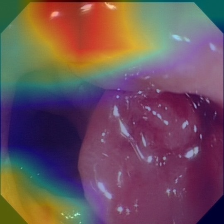

Supplement: Supplementary file 2 — Supplementary Data 1 [file 41467_2020_16777_MOESM2_ESM.gz › SupplementaryData1.36fn/17/IMG_01.0000000018312.0050.14505300660.jpg_benign_gcam_densenet169_finetune.png]

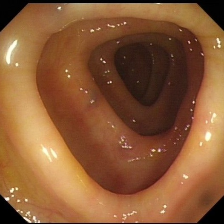

Supplement: Supplementary file 2 — Supplementary Data 1 [file 41467_2020_16777_MOESM2_ESM.gz › SupplementaryData1.36fn/17/IMG_01.0000000018312.0026.14471400972.jpg_benign_gcam_densenet169_finetune.png_raw_image.png]

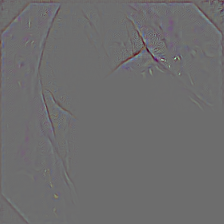

Supplement: Supplementary file 2 — Supplementary Data 1 [file 41467_2020_16777_MOESM2_ESM.gz › SupplementaryData1.36fn/17/IMG_01.0000000018312.0051.14510700606.jpg_benign_ggcam_densenet169_finetune.png]

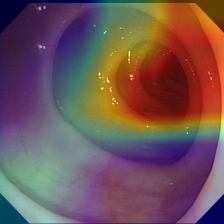

Supplement: Supplementary file 2 — Supplementary Data 1 [file 41467_2020_16777_MOESM2_ESM.gz › SupplementaryData1.36fn/17/IMG_01.0000000018312.0038.14491400687.jpg_benign_gcam_densenet169_finetune.png]

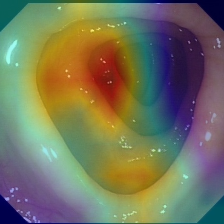

Supplement: Supplementary file 2 — Supplementary Data 1 [file 41467_2020_16777_MOESM2_ESM.gz › SupplementaryData1.36fn/17/IMG_01.0000000018312.0026.14471400972.jpg_benign_gcam_densenet169_finetune.png]

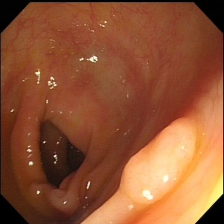

Supplement: Supplementary file 2 — Supplementary Data 1 [file 41467_2020_16777_MOESM2_ESM.gz › SupplementaryData1.36fn/17/IMG_01.0000000018312.0036.14490400383.jpg_benign_gcam_densenet169_finetune.png_raw_image.png]

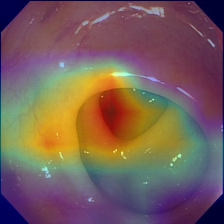

Supplement: Supplementary file 2 — Supplementary Data 1 [file 41467_2020_16777_MOESM2_ESM.gz › SupplementaryData1.36fn/17/IMG_01.0000000018312.0032.14480700479.jpg_benign_gcam_densenet169_finetune.png]

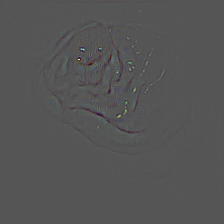

Supplement: Supplementary file 2 — Supplementary Data 1 [file 41467_2020_16777_MOESM2_ESM.gz › SupplementaryData1.36fn/17/IMG_01.0000000018312.0031.14474500373.jpg_benign_ggcam_densenet169_finetune.png]

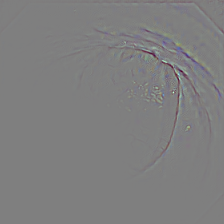

Supplement: Supplementary file 2 — Supplementary Data 1 [file 41467_2020_16777_MOESM2_ESM.gz › SupplementaryData1.36fn/17/IMG_01.0000000018312.0049.14504300269.jpg_benign_ggcam_densenet169_finetune.png]

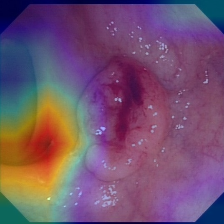

Supplement: Supplementary file 2 — Supplementary Data 1 [file 41467_2020_16777_MOESM2_ESM.gz › SupplementaryData1.36fn/17/IMG_01.0000000018312.0052.14511100508.jpg_benign_gcam_densenet169_finetune.png]

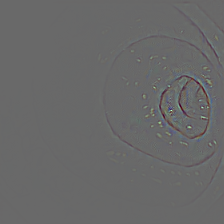

Supplement: Supplementary file 2 — Supplementary Data 1 [file 41467_2020_16777_MOESM2_ESM.gz › SupplementaryData1.36fn/17/IMG_01.0000000018312.0039.14491900224.jpg_benign_ggcam_densenet169_finetune.png]
