# Supplementary figures and images for: Diagnostic evaluation of a deep learning model for optical diagnosis of colorectal cancer (part 1 of 5)
Source: Nat Commun. 2020 Jun 11;11:2961. doi: 10.1038/s41467-020-16777-6 (PMC7289893; doi:10.1038/s41467-020-16777-6)

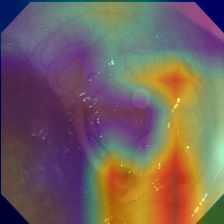

Supplement: Supplementary file 2 — Supplementary Data 1 [file 41467_2020_16777_MOESM2_ESM.gz › SupplementaryData1.36fn/20/IMG_01.0000000020799.0016.15035500293.jpg_benign_gcam_densenet169_finetune.png]

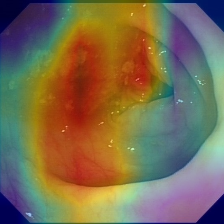

Supplement: Supplementary file 2 — Supplementary Data 1 [file 41467_2020_16777_MOESM2_ESM.gz › SupplementaryData1.36fn/20/IMG_01.0000000020799.0011.15025300077.jpg_benign_gcam_densenet169_finetune.png]

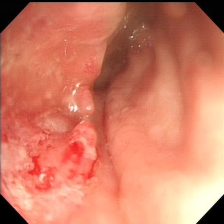

Supplement: Supplementary file 2 — Supplementary Data 1 [file 41467_2020_16777_MOESM2_ESM.gz › SupplementaryData1.36fn/20/IMG_01.0000000020799.0037.15072000058.jpg_malignant_gcam_densenet169_finetune.png_raw_image.png]

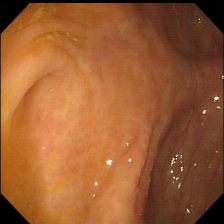

Supplement: Supplementary file 2 — Supplementary Data 1 [file 41467_2020_16777_MOESM2_ESM.gz › SupplementaryData1.36fn/20/IMG_01.0000000020799.0001.15004600802.jpg_benign_gcam_densenet169_finetune.png_raw_image.png]

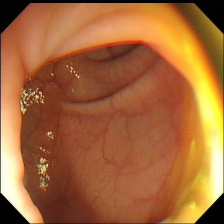

Supplement: Supplementary file 2 — Supplementary Data 1 [file 41467_2020_16777_MOESM2_ESM.gz › SupplementaryData1.36fn/20/IMG_01.0000000020799.0025.15053100324.jpg_benign_gcam_densenet169_finetune.png_raw_image.png]

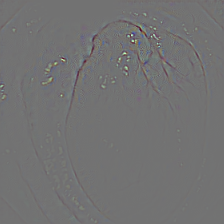

Supplement: Supplementary file 2 — Supplementary Data 1 [file 41467_2020_16777_MOESM2_ESM.gz › SupplementaryData1.36fn/20/IMG_01.0000000020799.0003.15015500083.jpg_benign_ggcam_densenet169_finetune.png]

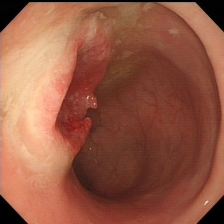

Supplement: Supplementary file 2 — Supplementary Data 1 [file 41467_2020_16777_MOESM2_ESM.gz › SupplementaryData1.36fn/20/IMG_01.0000000020799.0042.15092900931.jpg_malignant_gcam_densenet169_finetune.png_raw_image.png]

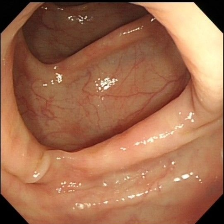

Supplement: Supplementary file 2 — Supplementary Data 1 [file 41467_2020_16777_MOESM2_ESM.gz › SupplementaryData1.36fn/20/IMG_01.0000000020799.0015.15032800336.jpg_benign_gcam_densenet169_finetune.png_raw_image.png]

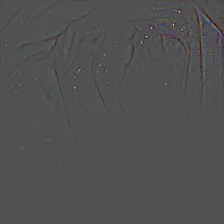

Supplement: Supplementary file 2 — Supplementary Data 1 [file 41467_2020_16777_MOESM2_ESM.gz › SupplementaryData1.36fn/20/IMG_01.0000000020799.0005.15020100399.jpg_benign_ggcam_densenet169_finetune.png]

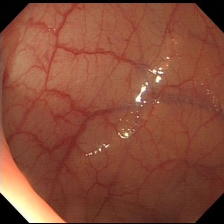

Supplement: Supplementary file 2 — Supplementary Data 1 [file 41467_2020_16777_MOESM2_ESM.gz › SupplementaryData1.36fn/20/IMG_01.0000000020799.0029.15060400231.jpg_benign_gcam_densenet169_finetune.png_raw_image.png]

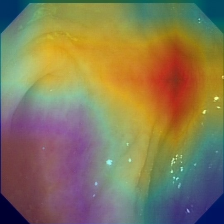

Supplement: Supplementary file 2 — Supplementary Data 1 [file 41467_2020_16777_MOESM2_ESM.gz › SupplementaryData1.36fn/20/IMG_01.0000000020799.0001.15004600802.jpg_benign_gcam_densenet169_finetune.png]

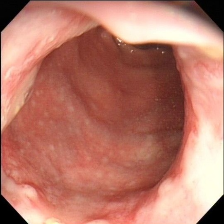

Supplement: Supplementary file 2 — Supplementary Data 1 [file 41467_2020_16777_MOESM2_ESM.gz › SupplementaryData1.36fn/20/IMG_01.0000000020799.0045.15165000324.jpg_benign_gcam_densenet169_finetune.png_raw_image.png]

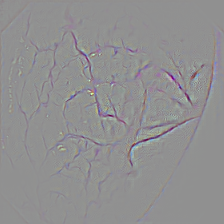

Supplement: Supplementary file 2 — Supplementary Data 1 [file 41467_2020_16777_MOESM2_ESM.gz › SupplementaryData1.36fn/20/IMG_01.0000000020799.0024.15052800248.jpg_benign_ggcam_densenet169_finetune.png]

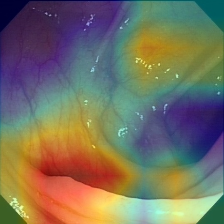

Supplement: Supplementary file 2 — Supplementary Data 1 [file 41467_2020_16777_MOESM2_ESM.gz › SupplementaryData1.36fn/20/IMG_01.0000000020799.0012.15030600043.jpg_benign_gcam_densenet169_finetune.png]

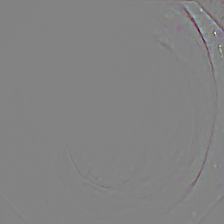

Supplement: Supplementary file 2 — Supplementary Data 1 [file 41467_2020_16777_MOESM2_ESM.gz › SupplementaryData1.36fn/20/IMG_01.0000000020799.0041.15092800013.jpg_benign_ggcam_densenet169_finetune.png]

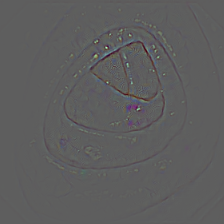

Supplement: Supplementary file 2 — Supplementary Data 1 [file 41467_2020_16777_MOESM2_ESM.gz › SupplementaryData1.36fn/20/IMG_01.0000000020799.0014.15032400101.jpg_benign_ggcam_densenet169_finetune.png]

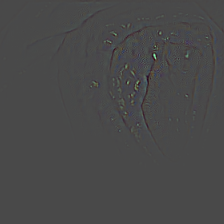

Supplement: Supplementary file 2 — Supplementary Data 1 [file 41467_2020_16777_MOESM2_ESM.gz › SupplementaryData1.36fn/20/IMG_01.0000000020799.0006.15020800927.jpg_benign_ggcam_densenet169_finetune.png]

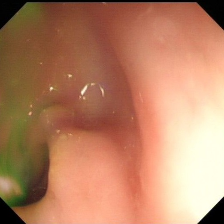

Supplement: Supplementary file 2 — Supplementary Data 1 [file 41467_2020_16777_MOESM2_ESM.gz › SupplementaryData1.36fn/20/IMG_01.0000000020799.0040.15074900554.jpg_benign_gcam_densenet169_finetune.png_raw_image.png]

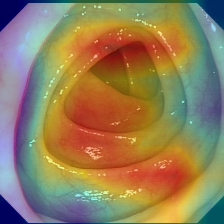

Supplement: Supplementary file 2 — Supplementary Data 1 [file 41467_2020_16777_MOESM2_ESM.gz › SupplementaryData1.36fn/20/IMG_01.0000000020799.0014.15032400101.jpg_benign_gcam_densenet169_finetune.png]

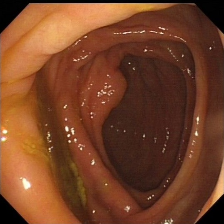

Supplement: Supplementary file 2 — Supplementary Data 1 [file 41467_2020_16777_MOESM2_ESM.gz › SupplementaryData1.36fn/20/IMG_01.0000000020799.0004.15015800915.jpg_benign_gcam_densenet169_finetune.png_raw_image.png]

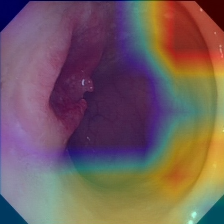

Supplement: Supplementary file 2 — Supplementary Data 1 [file 41467_2020_16777_MOESM2_ESM.gz › SupplementaryData1.36fn/20/IMG_01.0000000020799.0041.15092800013.jpg_benign_gcam_densenet169_finetune.png]

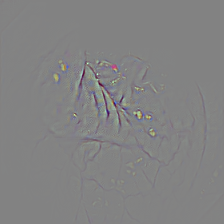

Supplement: Supplementary file 2 — Supplementary Data 1 [file 41467_2020_16777_MOESM2_ESM.gz › SupplementaryData1.36fn/20/IMG_01.0000000020799.0026.15054200726.jpg_benign_ggcam_densenet169_finetune.png]

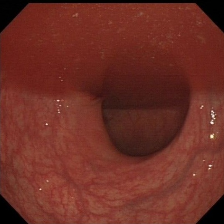

Supplement: Supplementary file 2 — Supplementary Data 1 [file 41467_2020_16777_MOESM2_ESM.gz › SupplementaryData1.36fn/20/IMG_01.0000000020799.0044.15164100708.jpg_benign_gcam_densenet169_finetune.png_raw_image.png]

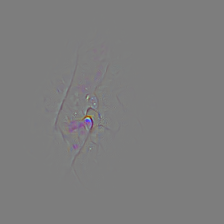

Supplement: Supplementary file 2 — Supplementary Data 1 [file 41467_2020_16777_MOESM2_ESM.gz › SupplementaryData1.36fn/20/IMG_01.0000000020799.0042.15092900931.jpg_malignant_ggcam_densenet169_finetune.png]

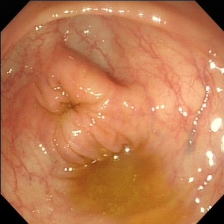

Supplement: Supplementary file 2 — Supplementary Data 1 [file 41467_2020_16777_MOESM2_ESM.gz › SupplementaryData1.36fn/20/IMG_01.0000000020799.0017.15042800841.jpg_benign_gcam_densenet169_finetune.png_raw_image.png]

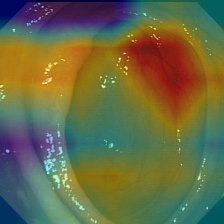

Supplement: Supplementary file 2 — Supplementary Data 1 [file 41467_2020_16777_MOESM2_ESM.gz › SupplementaryData1.36fn/20/IMG_01.0000000020799.0003.15015500083.jpg_benign_gcam_densenet169_finetune.png]

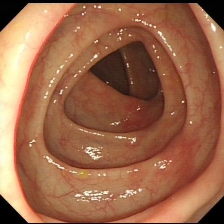

Supplement: Supplementary file 2 — Supplementary Data 1 [file 41467_2020_16777_MOESM2_ESM.gz › SupplementaryData1.36fn/20/IMG_01.0000000020799.0014.15032400101.jpg_benign_gcam_densenet169_finetune.png_raw_image.png]

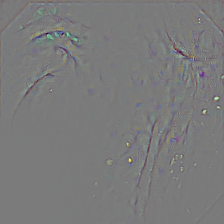

Supplement: Supplementary file 2 — Supplementary Data 1 [file 41467_2020_16777_MOESM2_ESM.gz › SupplementaryData1.36fn/20/IMG_01.0000000020799.0001.15004600802.jpg_benign_ggcam_densenet169_finetune.png]

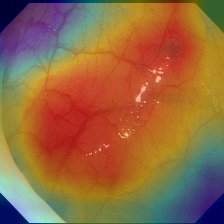

Supplement: Supplementary file 2 — Supplementary Data 1 [file 41467_2020_16777_MOESM2_ESM.gz › SupplementaryData1.36fn/20/IMG_01.0000000020799.0029.15060400231.jpg_benign_gcam_densenet169_finetune.png]

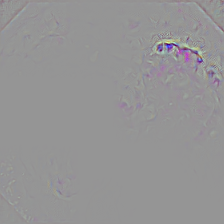

Supplement: Supplementary file 2 — Supplementary Data 1 [file 41467_2020_16777_MOESM2_ESM.gz › SupplementaryData1.36fn/20/IMG_01.0000000020799.0043.15131200457.jpg_benign_ggcam_densenet169_finetune.png]

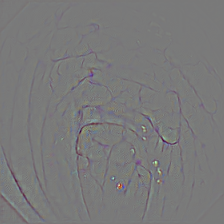

Supplement: Supplementary file 2 — Supplementary Data 1 [file 41467_2020_16777_MOESM2_ESM.gz › SupplementaryData1.36fn/20/IMG_01.0000000020799.0019.15043400491.jpg_benign_ggcam_densenet169_finetune.png]

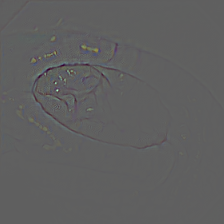

Supplement: Supplementary file 2 — Supplementary Data 1 [file 41467_2020_16777_MOESM2_ESM.gz › SupplementaryData1.36fn/20/IMG_01.0000000020799.0027.15055400244.jpg_benign_ggcam_densenet169_finetune.png]

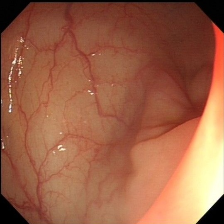

Supplement: Supplementary file 2 — Supplementary Data 1 [file 41467_2020_16777_MOESM2_ESM.gz › SupplementaryData1.36fn/20/IMG_01.0000000020799.0024.15052800248.jpg_benign_gcam_densenet169_finetune.png_raw_image.png]

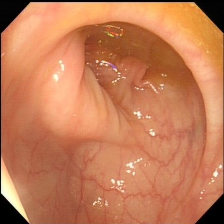

Supplement: Supplementary file 2 — Supplementary Data 1 [file 41467_2020_16777_MOESM2_ESM.gz › SupplementaryData1.36fn/20/IMG_01.0000000020799.0026.15054200726.jpg_benign_gcam_densenet169_finetune.png_raw_image.png]

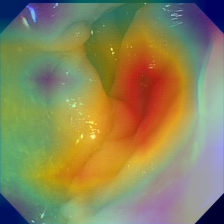

Supplement: Supplementary file 2 — Supplementary Data 1 [file 41467_2020_16777_MOESM2_ESM.gz › SupplementaryData1.36fn/20/IMG_01.0000000020799.0008.15022000660.jpg_benign_gcam_densenet169_finetune.png]

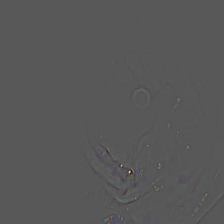

Supplement: Supplementary file 2 — Supplementary Data 1 [file 41467_2020_16777_MOESM2_ESM.gz › SupplementaryData1.36fn/20/IMG_01.0000000020799.0016.15035500293.jpg_benign_ggcam_densenet169_finetune.png]

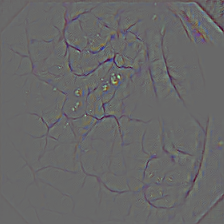

Supplement: Supplementary file 2 — Supplementary Data 1 [file 41467_2020_16777_MOESM2_ESM.gz › SupplementaryData1.36fn/20/IMG_01.0000000020799.0030.15060700893.jpg_benign_ggcam_densenet169_finetune.png]

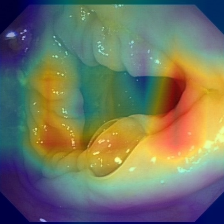

Supplement: Supplementary file 2 — Supplementary Data 1 [file 41467_2020_16777_MOESM2_ESM.gz › SupplementaryData1.36fn/20/IMG_01.0000000020799.0010.15025100186.jpg_benign_gcam_densenet169_finetune.png]

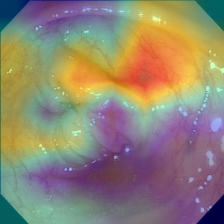

Supplement: Supplementary file 2 — Supplementary Data 1 [file 41467_2020_16777_MOESM2_ESM.gz › SupplementaryData1.36fn/20/IMG_01.0000000020799.0017.15042800841.jpg_benign_gcam_densenet169_finetune.png]

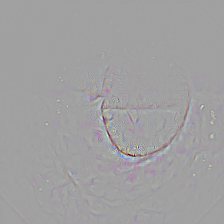

Supplement: Supplementary file 2 — Supplementary Data 1 [file 41467_2020_16777_MOESM2_ESM.gz › SupplementaryData1.36fn/20/IMG_01.0000000020799.0044.15164100708.jpg_benign_ggcam_densenet169_finetune.png]

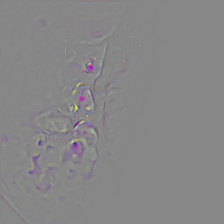

Supplement: Supplementary file 2 — Supplementary Data 1 [file 41467_2020_16777_MOESM2_ESM.gz › SupplementaryData1.36fn/20/IMG_01.0000000020799.0037.15072000058.jpg_malignant_ggcam_densenet169_finetune.png]

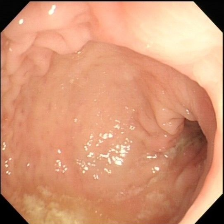

Supplement: Supplementary file 2 — Supplementary Data 1 [file 41467_2020_16777_MOESM2_ESM.gz › SupplementaryData1.36fn/20/IMG_01.0000000020799.0035.15065800059.jpg_benign_gcam_densenet169_finetune.png_raw_image.png]

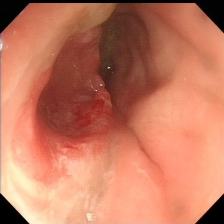

Supplement: Supplementary file 2 — Supplementary Data 1 [file 41467_2020_16777_MOESM2_ESM.gz › SupplementaryData1.36fn/20/IMG_01.0000000020799.0038.15072500015.jpg_benign_gcam_densenet169_finetune.png_raw_image.png]

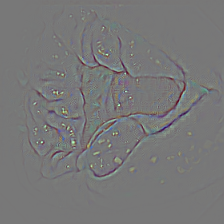

Supplement: Supplementary file 2 — Supplementary Data 1 [file 41467_2020_16777_MOESM2_ESM.gz › SupplementaryData1.36fn/20/IMG_01.0000000020799.0010.15025100186.jpg_benign_ggcam_densenet169_finetune.png]

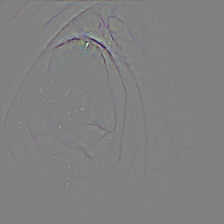

Supplement: Supplementary file 2 — Supplementary Data 1 [file 41467_2020_16777_MOESM2_ESM.gz › SupplementaryData1.36fn/20/IMG_01.0000000020799.0022.15045800311.jpg_benign_ggcam_densenet169_finetune.png]

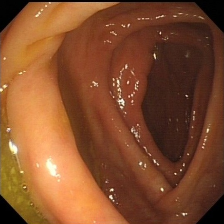

Supplement: Supplementary file 2 — Supplementary Data 1 [file 41467_2020_16777_MOESM2_ESM.gz › SupplementaryData1.36fn/20/IMG_01.0000000020799.0006.15020800927.jpg_benign_gcam_densenet169_finetune.png_raw_image.png]

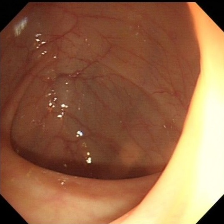

Supplement: Supplementary file 2 — Supplementary Data 1 [file 41467_2020_16777_MOESM2_ESM.gz › SupplementaryData1.36fn/20/IMG_01.0000000020799.0018.15043100932.jpg_benign_gcam_densenet169_finetune.png_raw_image.png]

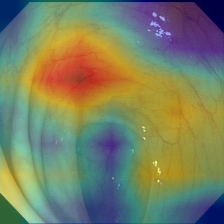

Supplement: Supplementary file 2 — Supplementary Data 1 [file 41467_2020_16777_MOESM2_ESM.gz › SupplementaryData1.36fn/20/IMG_01.0000000020799.0019.15043400491.jpg_benign_gcam_densenet169_finetune.png]

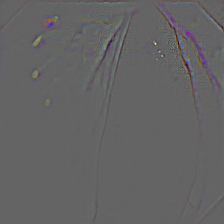

Supplement: Supplementary file 2 — Supplementary Data 1 [file 41467_2020_16777_MOESM2_ESM.gz › SupplementaryData1.36fn/20/IMG_01.0000000020799.0032.15062900518.jpg_benign_ggcam_densenet169_finetune.png]

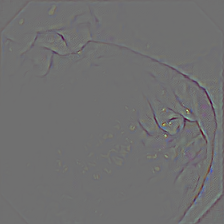

Supplement: Supplementary file 2 — Supplementary Data 1 [file 41467_2020_16777_MOESM2_ESM.gz › SupplementaryData1.36fn/20/IMG_01.0000000020799.0035.15065800059.jpg_benign_ggcam_densenet169_finetune.png]

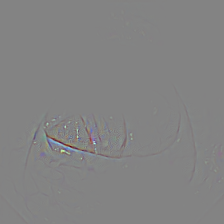

Supplement: Supplementary file 2 — Supplementary Data 1 [file 41467_2020_16777_MOESM2_ESM.gz › SupplementaryData1.36fn/20/IMG_01.0000000020799.0013.15031500134.jpg_benign_ggcam_densenet169_finetune.png]

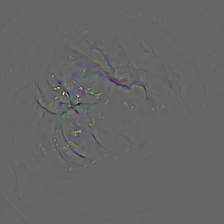

Supplement: Supplementary file 2 — Supplementary Data 1 [file 41467_2020_16777_MOESM2_ESM.gz › SupplementaryData1.36fn/20/IMG_01.0000000020799.0017.15042800841.jpg_benign_ggcam_densenet169_finetune.png]

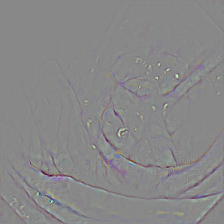

Supplement: Supplementary file 2 — Supplementary Data 1 [file 41467_2020_16777_MOESM2_ESM.gz › SupplementaryData1.36fn/20/IMG_01.0000000020799.0012.15030600043.jpg_benign_ggcam_densenet169_finetune.png]

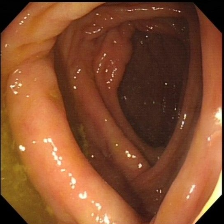

Supplement: Supplementary file 2 — Supplementary Data 1 [file 41467_2020_16777_MOESM2_ESM.gz › SupplementaryData1.36fn/20/IMG_01.0000000020799.0005.15020100399.jpg_benign_gcam_densenet169_finetune.png_raw_image.png]

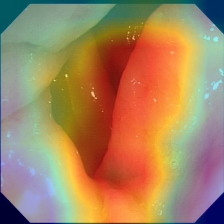

Supplement: Supplementary file 2 — Supplementary Data 1 [file 41467_2020_16777_MOESM2_ESM.gz › SupplementaryData1.36fn/20/IMG_01.0000000020799.0007.15021800493.jpg_benign_gcam_densenet169_finetune.png]

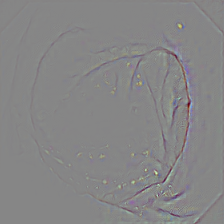

Supplement: Supplementary file 2 — Supplementary Data 1 [file 41467_2020_16777_MOESM2_ESM.gz › SupplementaryData1.36fn/20/IMG_01.0000000020799.0034.15063500919.jpg_benign_ggcam_densenet169_finetune.png]

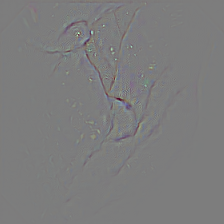

Supplement: Supplementary file 2 — Supplementary Data 1 [file 41467_2020_16777_MOESM2_ESM.gz › SupplementaryData1.36fn/20/IMG_01.0000000020799.0008.15022000660.jpg_benign_ggcam_densenet169_finetune.png]

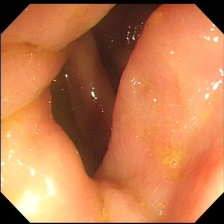

Supplement: Supplementary file 2 — Supplementary Data 1 [file 41467_2020_16777_MOESM2_ESM.gz › SupplementaryData1.36fn/20/IMG_01.0000000020799.0007.15021800493.jpg_benign_gcam_densenet169_finetune.png_raw_image.png]

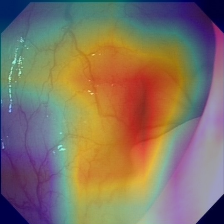

Supplement: Supplementary file 2 — Supplementary Data 1 [file 41467_2020_16777_MOESM2_ESM.gz › SupplementaryData1.36fn/20/IMG_01.0000000020799.0024.15052800248.jpg_benign_gcam_densenet169_finetune.png]

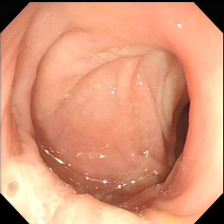

Supplement: Supplementary file 2 — Supplementary Data 1 [file 41467_2020_16777_MOESM2_ESM.gz › SupplementaryData1.36fn/20/IMG_01.0000000020799.0034.15063500919.jpg_benign_gcam_densenet169_finetune.png_raw_image.png]

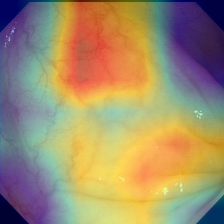

Supplement: Supplementary file 2 — Supplementary Data 1 [file 41467_2020_16777_MOESM2_ESM.gz › SupplementaryData1.36fn/20/IMG_01.0000000020799.0023.15051400636.jpg_benign_gcam_densenet169_finetune.png]

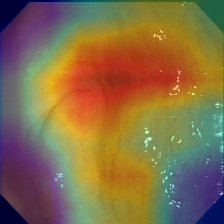

Supplement: Supplementary file 2 — Supplementary Data 1 [file 41467_2020_16777_MOESM2_ESM.gz › SupplementaryData1.36fn/20/IMG_01.0000000020799.0002.15013600959.jpg_benign_gcam_densenet169_finetune.png]

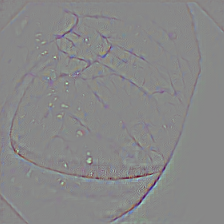

Supplement: Supplementary file 2 — Supplementary Data 1 [file 41467_2020_16777_MOESM2_ESM.gz › SupplementaryData1.36fn/20/IMG_01.0000000020799.0018.15043100932.jpg_benign_ggcam_densenet169_finetune.png]

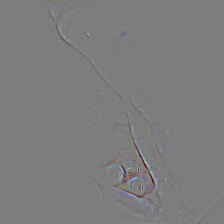

Supplement: Supplementary file 2 — Supplementary Data 1 [file 41467_2020_16777_MOESM2_ESM.gz › SupplementaryData1.36fn/20/IMG_01.0000000020799.0036.15070600289.jpg_benign_ggcam_densenet169_finetune.png]

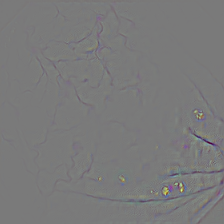

Supplement: Supplementary file 2 — Supplementary Data 1 [file 41467_2020_16777_MOESM2_ESM.gz › SupplementaryData1.36fn/20/IMG_01.0000000020799.0023.15051400636.jpg_benign_ggcam_densenet169_finetune.png]

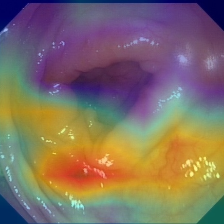

Supplement: Supplementary file 2 — Supplementary Data 1 [file 41467_2020_16777_MOESM2_ESM.gz › SupplementaryData1.36fn/20/IMG_01.0000000020799.0009.15024800202.jpg_benign_gcam_densenet169_finetune.png]

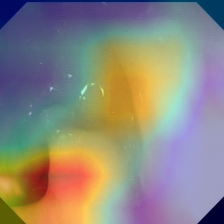

Supplement: Supplementary file 2 — Supplementary Data 1 [file 41467_2020_16777_MOESM2_ESM.gz › SupplementaryData1.36fn/20/IMG_01.0000000020799.0040.15074900554.jpg_benign_gcam_densenet169_finetune.png]

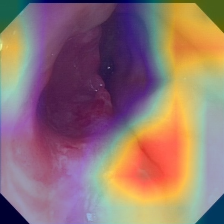

Supplement: Supplementary file 2 — Supplementary Data 1 [file 41467_2020_16777_MOESM2_ESM.gz › SupplementaryData1.36fn/20/IMG_01.0000000020799.0038.15072500015.jpg_benign_gcam_densenet169_finetune.png]

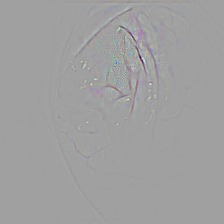

Supplement: Supplementary file 2 — Supplementary Data 1 [file 41467_2020_16777_MOESM2_ESM.gz › SupplementaryData1.36fn/20/IMG_01.0000000020799.0021.15045400201.jpg_benign_ggcam_densenet169_finetune.png]

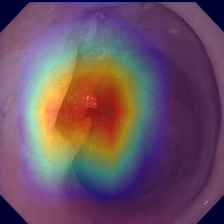

Supplement: Supplementary file 2 — Supplementary Data 1 [file 41467_2020_16777_MOESM2_ESM.gz › SupplementaryData1.36fn/20/IMG_01.0000000020799.0042.15092900931.jpg_malignant_gcam_densenet169_finetune.png]

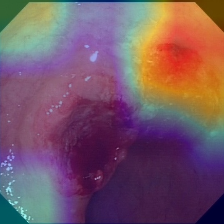

Supplement: Supplementary file 2 — Supplementary Data 1 [file 41467_2020_16777_MOESM2_ESM.gz › SupplementaryData1.36fn/20/IMG_01.0000000020799.0043.15131200457.jpg_benign_gcam_densenet169_finetune.png]

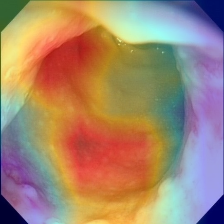

Supplement: Supplementary file 2 — Supplementary Data 1 [file 41467_2020_16777_MOESM2_ESM.gz › SupplementaryData1.36fn/20/IMG_01.0000000020799.0045.15165000324.jpg_benign_gcam_densenet169_finetune.png]

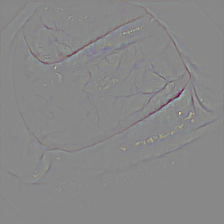

Supplement: Supplementary file 2 — Supplementary Data 1 [file 41467_2020_16777_MOESM2_ESM.gz › SupplementaryData1.36fn/20/IMG_01.0000000020799.0015.15032800336.jpg_benign_ggcam_densenet169_finetune.png]

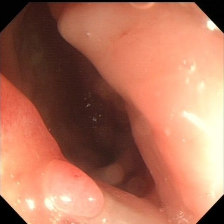

Supplement: Supplementary file 2 — Supplementary Data 1 [file 41467_2020_16777_MOESM2_ESM.gz › SupplementaryData1.36fn/20/IMG_01.0000000020799.0036.15070600289.jpg_benign_gcam_densenet169_finetune.png_raw_image.png]

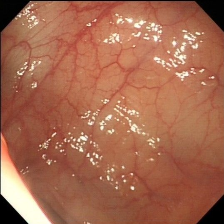

Supplement: Supplementary file 2 — Supplementary Data 1 [file 41467_2020_16777_MOESM2_ESM.gz › SupplementaryData1.36fn/20/IMG_01.0000000020799.0028.15055800619.jpg_benign_gcam_densenet169_finetune.png_raw_image.png]

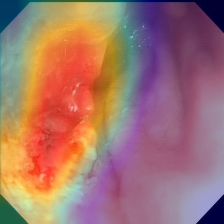

Supplement: Supplementary file 2 — Supplementary Data 1 [file 41467_2020_16777_MOESM2_ESM.gz › SupplementaryData1.36fn/20/IMG_01.0000000020799.0037.15072000058.jpg_malignant_gcam_densenet169_finetune.png]

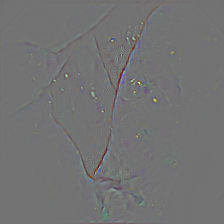

Supplement: Supplementary file 2 — Supplementary Data 1 [file 41467_2020_16777_MOESM2_ESM.gz › SupplementaryData1.36fn/20/IMG_01.0000000020799.0007.15021800493.jpg_benign_ggcam_densenet169_finetune.png]

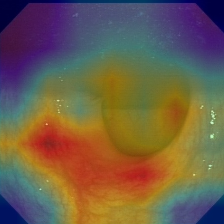

Supplement: Supplementary file 2 — Supplementary Data 1 [file 41467_2020_16777_MOESM2_ESM.gz › SupplementaryData1.36fn/20/IMG_01.0000000020799.0044.15164100708.jpg_benign_gcam_densenet169_finetune.png]

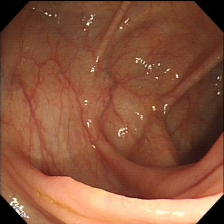

Supplement: Supplementary file 2 — Supplementary Data 1 [file 41467_2020_16777_MOESM2_ESM.gz › SupplementaryData1.36fn/20/IMG_01.0000000020799.0012.15030600043.jpg_benign_gcam_densenet169_finetune.png_raw_image.png]

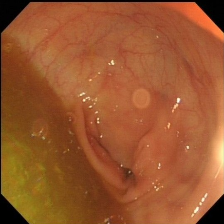

Supplement: Supplementary file 2 — Supplementary Data 1 [file 41467_2020_16777_MOESM2_ESM.gz › SupplementaryData1.36fn/20/IMG_01.0000000020799.0016.15035500293.jpg_benign_gcam_densenet169_finetune.png_raw_image.png]

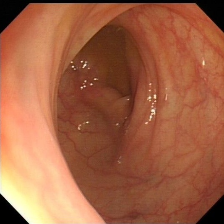

Supplement: Supplementary file 2 — Supplementary Data 1 [file 41467_2020_16777_MOESM2_ESM.gz › SupplementaryData1.36fn/20/IMG_01.0000000020799.0021.15045400201.jpg_benign_gcam_densenet169_finetune.png_raw_image.png]

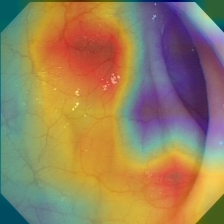

Supplement: Supplementary file 2 — Supplementary Data 1 [file 41467_2020_16777_MOESM2_ESM.gz › SupplementaryData1.36fn/20/IMG_01.0000000020799.0030.15060700893.jpg_benign_gcam_densenet169_finetune.png]

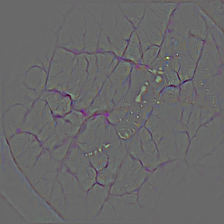

Supplement: Supplementary file 2 — Supplementary Data 1 [file 41467_2020_16777_MOESM2_ESM.gz › SupplementaryData1.36fn/20/IMG_01.0000000020799.0029.15060400231.jpg_benign_ggcam_densenet169_finetune.png]

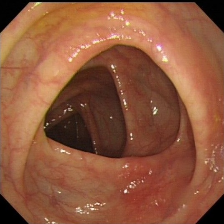

Supplement: Supplementary file 2 — Supplementary Data 1 [file 41467_2020_16777_MOESM2_ESM.gz › SupplementaryData1.36fn/20/IMG_01.0000000020799.0013.15031500134.jpg_benign_gcam_densenet169_finetune.png_raw_image.png]

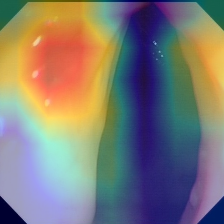

Supplement: Supplementary file 2 — Supplementary Data 1 [file 41467_2020_16777_MOESM2_ESM.gz › SupplementaryData1.36fn/20/IMG_01.0000000020799.0032.15062900518.jpg_benign_gcam_densenet169_finetune.png]

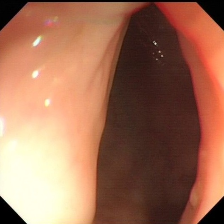

Supplement: Supplementary file 2 — Supplementary Data 1 [file 41467_2020_16777_MOESM2_ESM.gz › SupplementaryData1.36fn/20/IMG_01.0000000020799.0032.15062900518.jpg_benign_gcam_densenet169_finetune.png_raw_image.png]

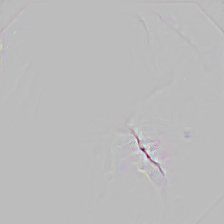

Supplement: Supplementary file 2 — Supplementary Data 1 [file 41467_2020_16777_MOESM2_ESM.gz › SupplementaryData1.36fn/20/IMG_01.0000000020799.0038.15072500015.jpg_benign_ggcam_densenet169_finetune.png]

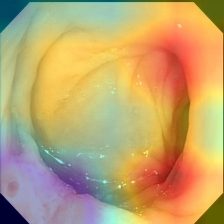

Supplement: Supplementary file 2 — Supplementary Data 1 [file 41467_2020_16777_MOESM2_ESM.gz › SupplementaryData1.36fn/20/IMG_01.0000000020799.0034.15063500919.jpg_benign_gcam_densenet169_finetune.png]

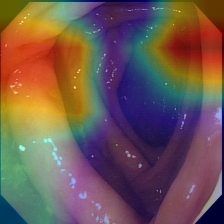

Supplement: Supplementary file 2 — Supplementary Data 1 [file 41467_2020_16777_MOESM2_ESM.gz › SupplementaryData1.36fn/20/IMG_01.0000000020799.0005.15020100399.jpg_benign_gcam_densenet169_finetune.png]

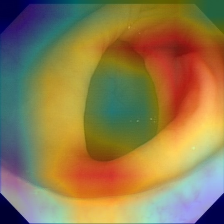

Supplement: Supplementary file 2 — Supplementary Data 1 [file 41467_2020_16777_MOESM2_ESM.gz › SupplementaryData1.36fn/20/IMG_01.0000000020799.0031.15061100696.jpg_benign_gcam_densenet169_finetune.png]

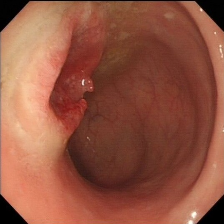

Supplement: Supplementary file 2 — Supplementary Data 1 [file 41467_2020_16777_MOESM2_ESM.gz › SupplementaryData1.36fn/20/IMG_01.0000000020799.0041.15092800013.jpg_benign_gcam_densenet169_finetune.png_raw_image.png]

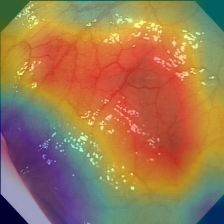

Supplement: Supplementary file 2 — Supplementary Data 1 [file 41467_2020_16777_MOESM2_ESM.gz › SupplementaryData1.36fn/20/IMG_01.0000000020799.0028.15055800619.jpg_benign_gcam_densenet169_finetune.png]

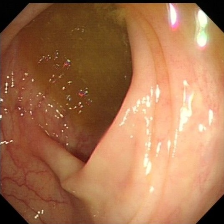

Supplement: Supplementary file 2 — Supplementary Data 1 [file 41467_2020_16777_MOESM2_ESM.gz › SupplementaryData1.36fn/20/IMG_01.0000000020799.0020.15043900934.jpg_benign_gcam_densenet169_finetune.png_raw_image.png]

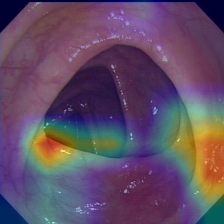

Supplement: Supplementary file 2 — Supplementary Data 1 [file 41467_2020_16777_MOESM2_ESM.gz › SupplementaryData1.36fn/20/IMG_01.0000000020799.0013.15031500134.jpg_benign_gcam_densenet169_finetune.png]

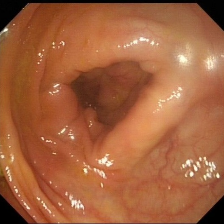

Supplement: Supplementary file 2 — Supplementary Data 1 [file 41467_2020_16777_MOESM2_ESM.gz › SupplementaryData1.36fn/20/IMG_01.0000000020799.0009.15024800202.jpg_benign_gcam_densenet169_finetune.png_raw_image.png]

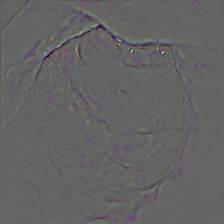

Supplement: Supplementary file 2 — Supplementary Data 1 [file 41467_2020_16777_MOESM2_ESM.gz › SupplementaryData1.36fn/20/IMG_01.0000000020799.0045.15165000324.jpg_benign_ggcam_densenet169_finetune.png]

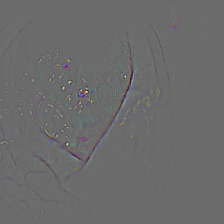

Supplement: Supplementary file 2 — Supplementary Data 1 [file 41467_2020_16777_MOESM2_ESM.gz › SupplementaryData1.36fn/20/IMG_01.0000000020799.0020.15043900934.jpg_benign_ggcam_densenet169_finetune.png]

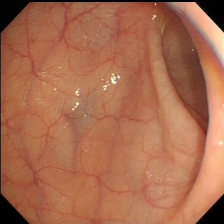

Supplement: Supplementary file 2 — Supplementary Data 1 [file 41467_2020_16777_MOESM2_ESM.gz › SupplementaryData1.36fn/20/IMG_01.0000000020799.0030.15060700893.jpg_benign_gcam_densenet169_finetune.png_raw_image.png]

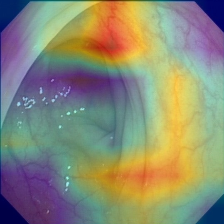

Supplement: Supplementary file 2 — Supplementary Data 1 [file 41467_2020_16777_MOESM2_ESM.gz › SupplementaryData1.36fn/20/IMG_01.0000000020799.0022.15045800311.jpg_benign_gcam_densenet169_finetune.png]

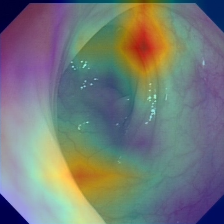

Supplement: Supplementary file 2 — Supplementary Data 1 [file 41467_2020_16777_MOESM2_ESM.gz › SupplementaryData1.36fn/20/IMG_01.0000000020799.0021.15045400201.jpg_benign_gcam_densenet169_finetune.png]
