# Supplementary material for: Diagnostic evaluation of a deep learning model for optical diagnosis of colorectal cancer
Source: Nat Commun. 2020 Jun 11;11:2961. doi: 10.1038/s41467-020-16777-6 (PMC7289893; doi:10.1038/s41467-020-16777-6)
Supplement: Supplementary file 3 — Supplementary Data 2 [file 41467_2020_16777_MOESM3_ESM.pdf]

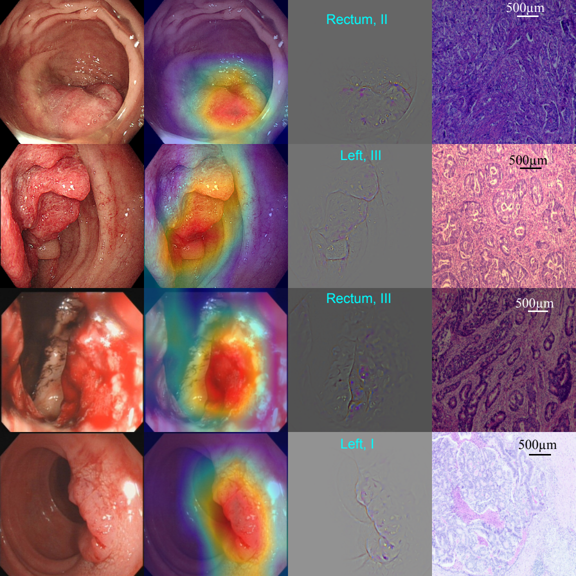
Rectum, II
500µm
Left, III
500µm
Rectum, III
500µm
Left, I
500µm

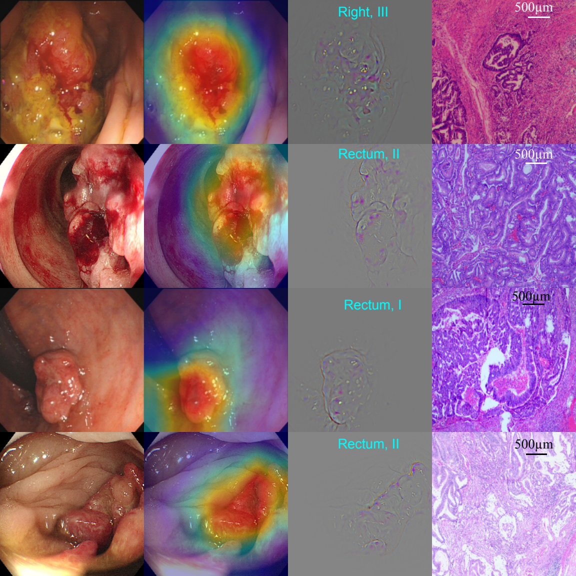
Right, III
500μm
Rectum, II
500μm
Rectum, I
500μm
Rectum, II
500μm

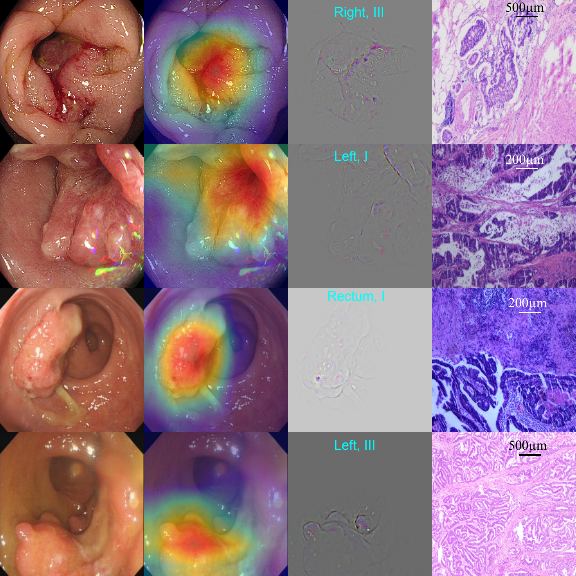
Right, III
500μm
Left, I
200μm
Rectum, I
200μm
Left, III
500μm

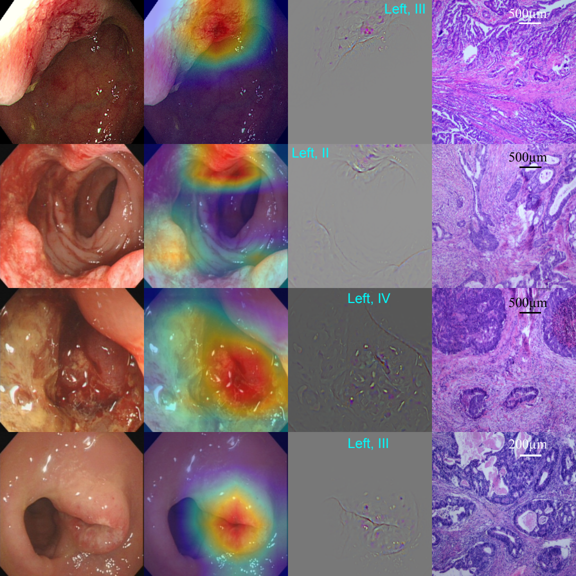
Left, III
500μm
Left, II
500μm
Left, IV
500μm
Left, III
200μm

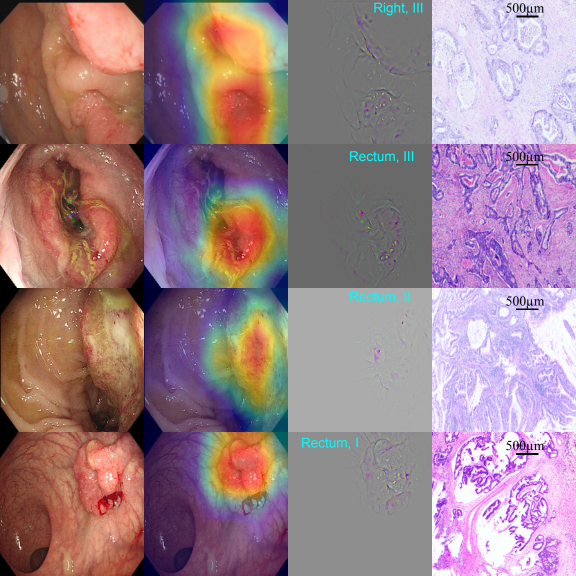
Right, III
500μm
Rectum, III
500μm
Rectum, II
500μm
Rectum, I
500μm

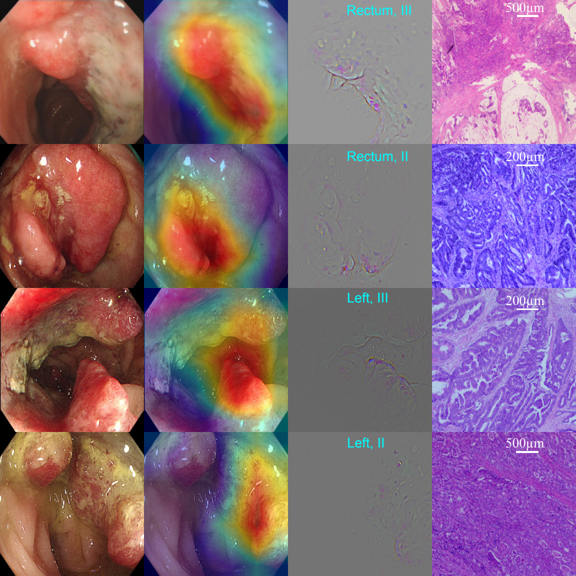

Rectum, III
500μm
Rectum, II
200μm
Left, III
200μm
Left, II
500μm

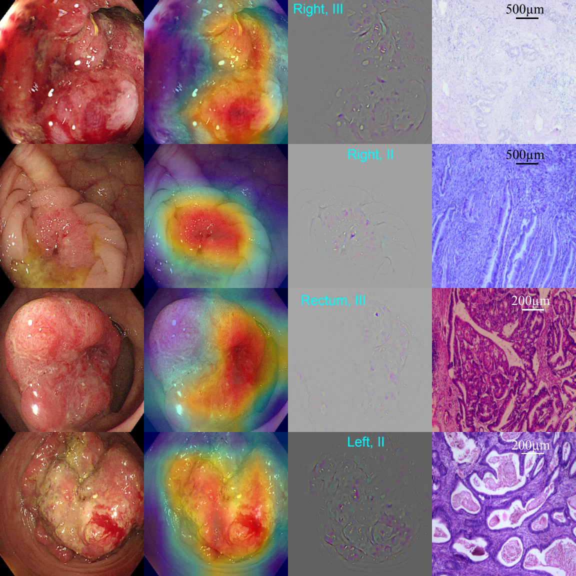
Right, III
500μm
Right, II
500μm
Rectum, III
200μm
Left, II
200μm

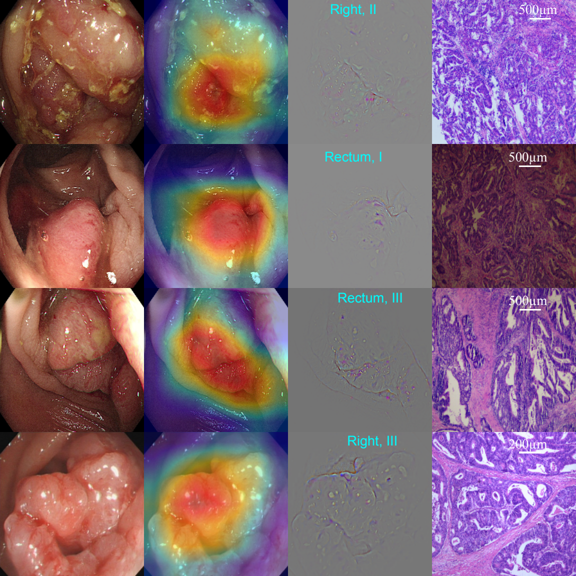
Right, II
500μm
Rectum, I
500μm
Rectum, III
500μm
Right, III
200μm

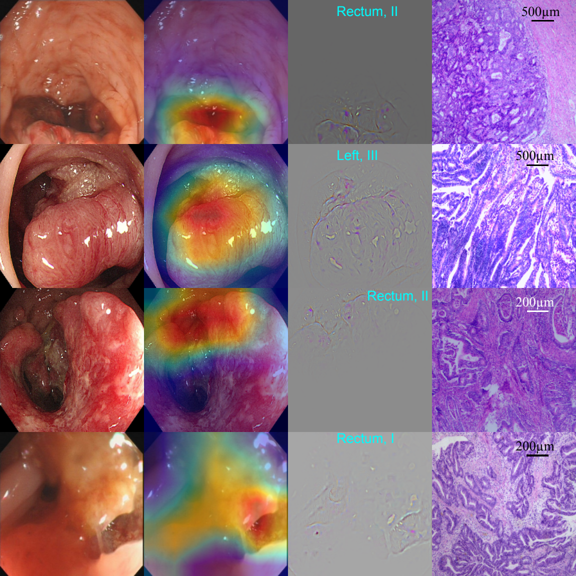
Rectum, II
500μm
Left, III
500μm
Rectum, II
200μm
Rectum, I
200μm

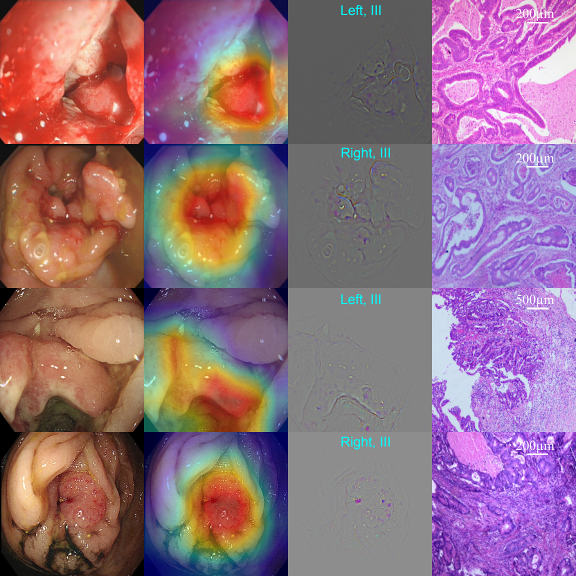

Left, III
200µm
Right, III
200µm
Left, III
500µm
Right, III
200µm

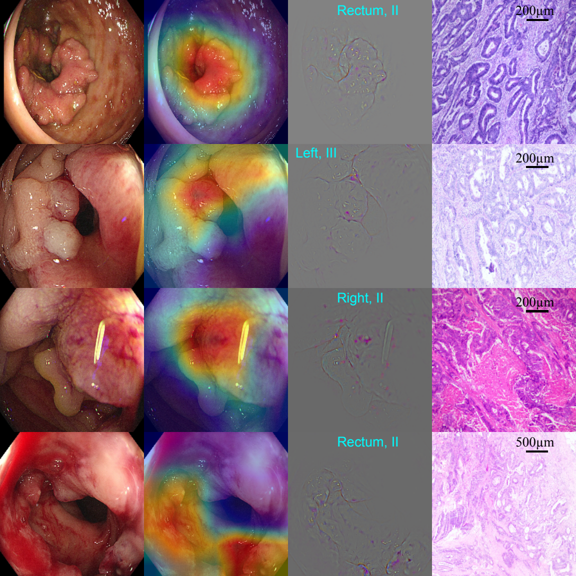
Rectum, II
200μm
Left, III
200μm
Right, II
200μm
Rectum, II
500μm
