# Supplementary figures and images for: Diagnostic evaluation of a deep learning model for optical diagnosis of colorectal cancer (part 3 of 5)
Source: Nat Commun. 2020 Jun 11;11:2961. doi: 10.1038/s41467-020-16777-6 (PMC7289893; doi:10.1038/s41467-020-16777-6)

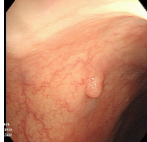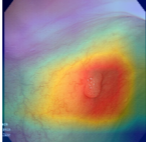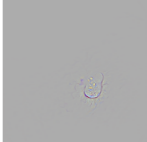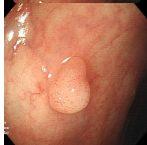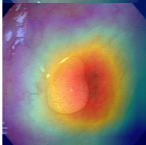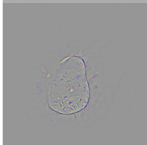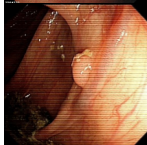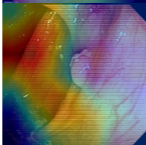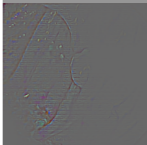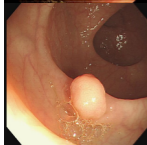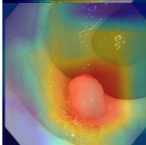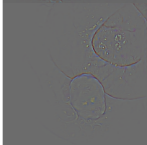

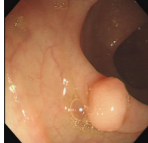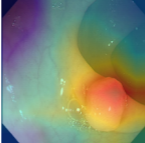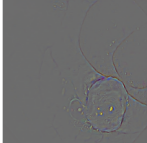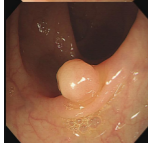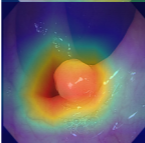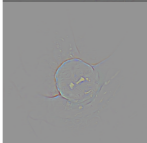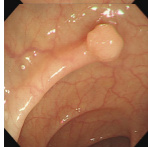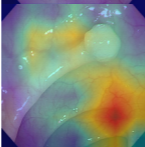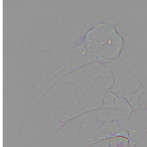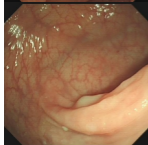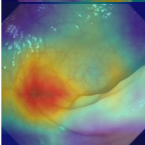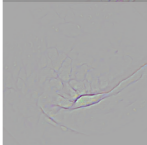

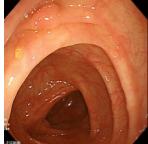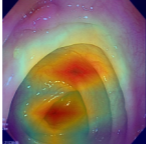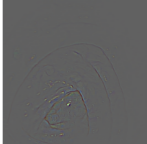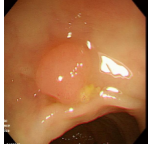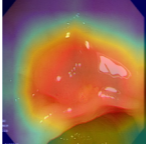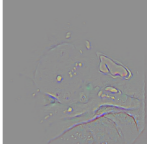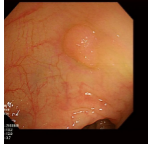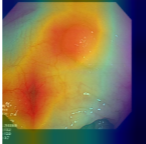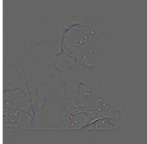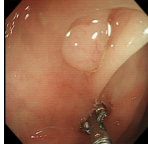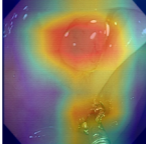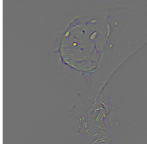

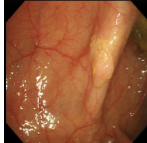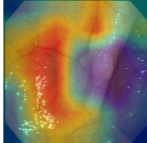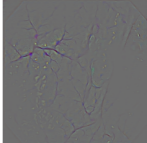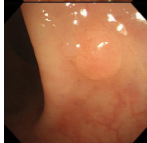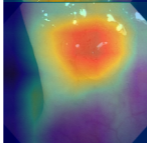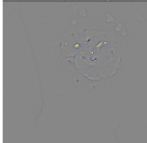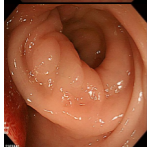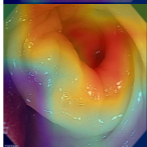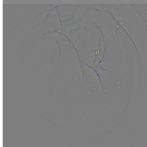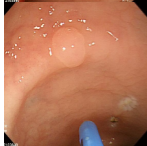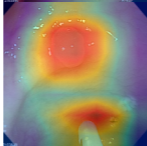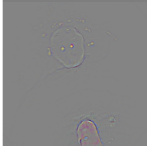

Supplement: Supplementary file 4 — Supplementary Data 3 [file 41467_2020_16777_MOESM4_ESM.pdf]

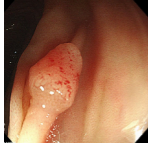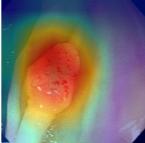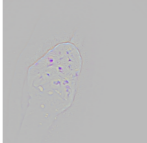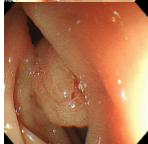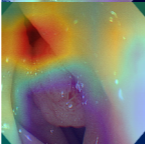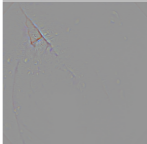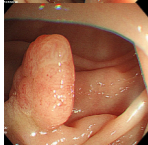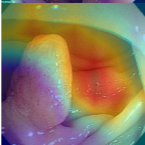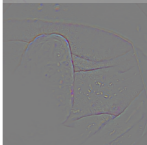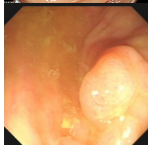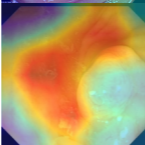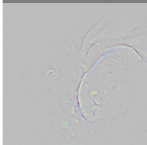

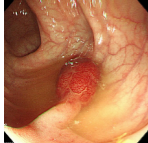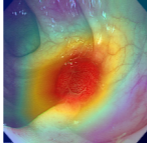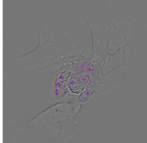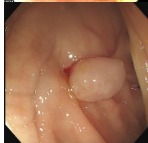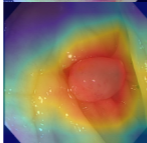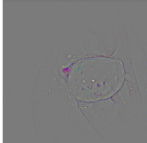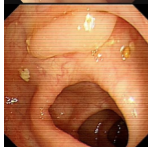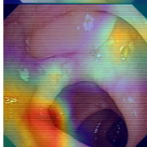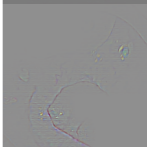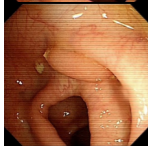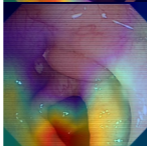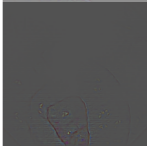

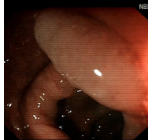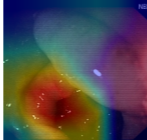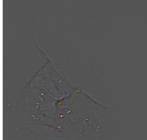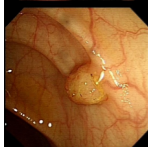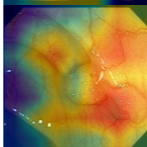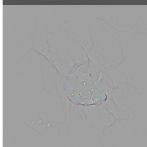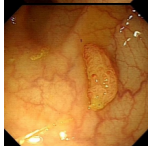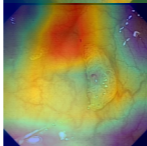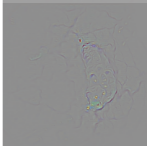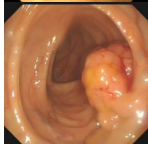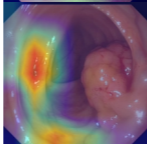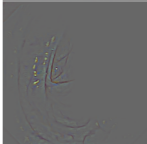

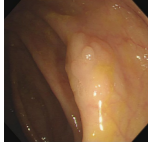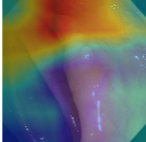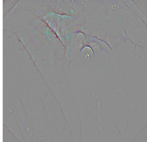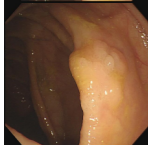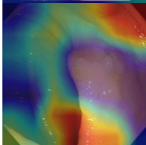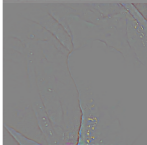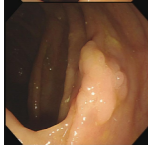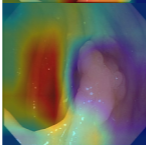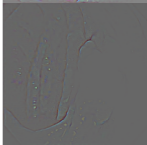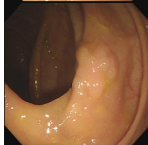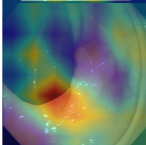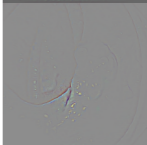

Supplement: Supplementary file 5 — Supplementary Data 4 [file 41467_2020_16777_MOESM5_ESM.pdf]

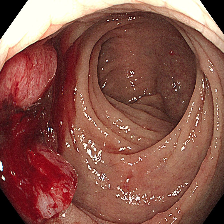

Supplement: Supplementary file 6 — Supplementary Data 5 [file 41467_2020_16777_MOESM6_ESM.gz › SupplementaryData5.255gradcam_heatmaps/IMG_01.201904250032.01.0015.1556159078.jpg_malignant_gcam_densenet169_finetune.png_raw_image.png]

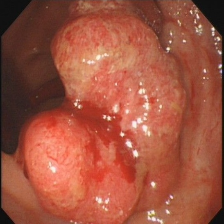

Supplement: Supplementary file 6 — Supplementary Data 5 [file 41467_2020_16777_MOESM6_ESM.gz › SupplementaryData5.255gradcam_heatmaps/IMG_01.201905200027.01.0062.1558316516.jpg_malignant_gcam_densenet169_finetune.png_raw_image.png]

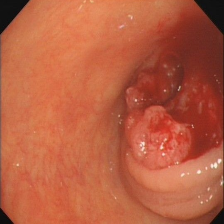

Supplement: Supplementary file 6 — Supplementary Data 5 [file 41467_2020_16777_MOESM6_ESM.gz › SupplementaryData5.255gradcam_heatmaps/IMG_01.201904160035.01.0031.1555381286.jpg_malignant_gcam_densenet169_finetune.png_raw_image.png]

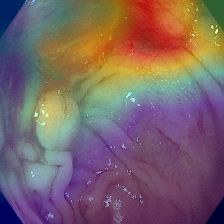

Supplement: Supplementary file 6 — Supplementary Data 5 [file 41467_2020_16777_MOESM6_ESM.gz › SupplementaryData5.255gradcam_heatmaps/IMG_01.201904300043.02.0074.1557122240.jpg_malignant_gcam_densenet169_finetune.png]

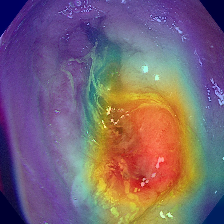

Supplement: Supplementary file 6 — Supplementary Data 5 [file 41467_2020_16777_MOESM6_ESM.gz › SupplementaryData5.255gradcam_heatmaps/IMG_01.201904170017.01.0001.1555464449.jpg_malignant_gcam_densenet169_finetune.png]

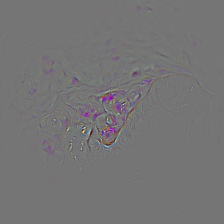

Supplement: Supplementary file 6 — Supplementary Data 5 [file 41467_2020_16777_MOESM6_ESM.gz › SupplementaryData5.255gradcam_heatmaps/IMG_01.201904220033.01.0031.1555898644.jpg_malignant_ggcam_densenet169_finetune.png]

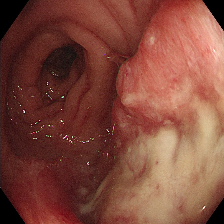

Supplement: Supplementary file 6 — Supplementary Data 5 [file 41467_2020_16777_MOESM6_ESM.gz › SupplementaryData5.255gradcam_heatmaps/IMG_01.201905090079.01.0131.1557387129.jpg_malignant_gcam_densenet169_finetune.png_raw_image.png]

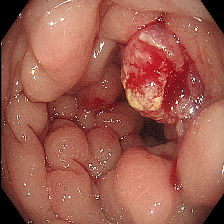

Supplement: Supplementary file 6 — Supplementary Data 5 [file 41467_2020_16777_MOESM6_ESM.gz › SupplementaryData5.255gradcam_heatmaps/IMG_01.201904250027.01.0021.1556158359.jpg_malignant_gcam_densenet169_finetune.png_raw_image.png]

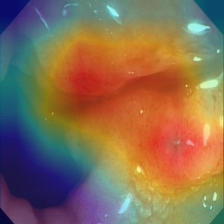

Supplement: Supplementary file 6 — Supplementary Data 5 [file 41467_2020_16777_MOESM6_ESM.gz › SupplementaryData5.255gradcam_heatmaps/IMG_01.201904020042.01.0069.1554171177.jpg_malignant_gcam_densenet169_finetune.png]

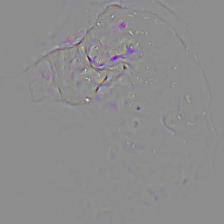

Supplement: Supplementary file 6 — Supplementary Data 5 [file 41467_2020_16777_MOESM6_ESM.gz › SupplementaryData5.255gradcam_heatmaps/IMG_01.201905150002.01.0012.1557878732.jpg_malignant_ggcam_densenet169_finetune.png]

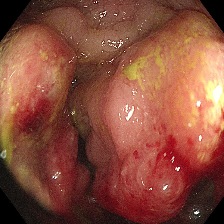

Supplement: Supplementary file 6 — Supplementary Data 5 [file 41467_2020_16777_MOESM6_ESM.gz › SupplementaryData5.255gradcam_heatmaps/IMG_01.201904170017.01.0038.1555465003.jpg_malignant_gcam_densenet169_finetune.png_raw_image.png]

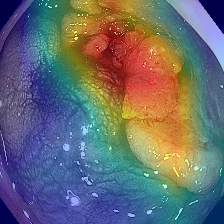

Supplement: Supplementary file 6 — Supplementary Data 5 [file 41467_2020_16777_MOESM6_ESM.gz › SupplementaryData5.255gradcam_heatmaps/IMG_01.201904010042.01.0013.1554087498.jpg_malignant_gcam_densenet169_finetune.png]

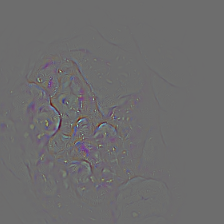

Supplement: Supplementary file 6 — Supplementary Data 5 [file 41467_2020_16777_MOESM6_ESM.gz › SupplementaryData5.255gradcam_heatmaps/IMG_01.201905230001.01.0011.1558570295.jpg_malignant_ggcam_densenet169_finetune.png]

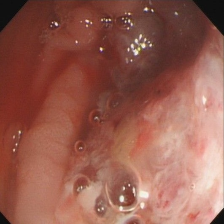

Supplement: Supplementary file 6 — Supplementary Data 5 [file 41467_2020_16777_MOESM6_ESM.gz › SupplementaryData5.255gradcam_heatmaps/IMG_01.201904160035.01.0005.1555381149.jpg_malignant_gcam_densenet169_finetune.png_raw_image.png]

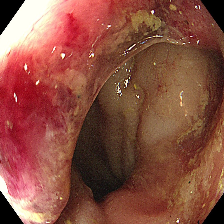

Supplement: Supplementary file 6 — Supplementary Data 5 [file 41467_2020_16777_MOESM6_ESM.gz › SupplementaryData5.255gradcam_heatmaps/IMG_01.201904090018.01.0081.1554772863.jpg_malignant_gcam_densenet169_finetune.png_raw_image.png]

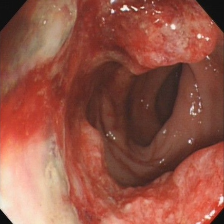

Supplement: Supplementary file 6 — Supplementary Data 5 [file 41467_2020_16777_MOESM6_ESM.gz › SupplementaryData5.255gradcam_heatmaps/IMG_01.201904110003.01.0093.1554941581.jpg_malignant_gcam_densenet169_finetune.png_raw_image.png]

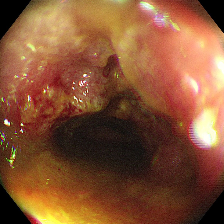

Supplement: Supplementary file 6 — Supplementary Data 5 [file 41467_2020_16777_MOESM6_ESM.gz › SupplementaryData5.255gradcam_heatmaps/IMG_01.201904240001.01.0008.1556064328.jpg_malignant_gcam_densenet169_finetune.png_raw_image.png]

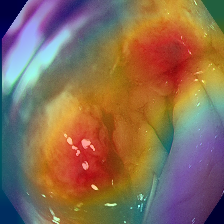

Supplement: Supplementary file 6 — Supplementary Data 5 [file 41467_2020_16777_MOESM6_ESM.gz › SupplementaryData5.255gradcam_heatmaps/IMG_01.201905160002.01.0036.1557964883.jpg_malignant_gcam_densenet169_finetune.png]

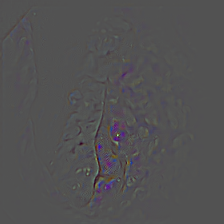

Supplement: Supplementary file 6 — Supplementary Data 5 [file 41467_2020_16777_MOESM6_ESM.gz › SupplementaryData5.255gradcam_heatmaps/IMG_01.201904010022.01.0096.1554081615.jpg_malignant_ggcam_densenet169_finetune.png]

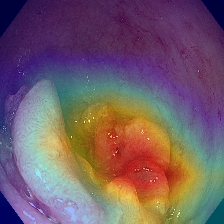

Supplement: Supplementary file 6 — Supplementary Data 5 [file 41467_2020_16777_MOESM6_ESM.gz › SupplementaryData5.255gradcam_heatmaps/IMG_01.201905230035.01.0004.1558577967.jpg_malignant_gcam_densenet169_finetune.png]

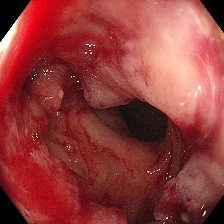

Supplement: Supplementary file 6 — Supplementary Data 5 [file 41467_2020_16777_MOESM6_ESM.gz › SupplementaryData5.255gradcam_heatmaps/IMG_01.201905090045.01.0055.1557369528.jpg_malignant_gcam_densenet169_finetune.png_raw_image.png]

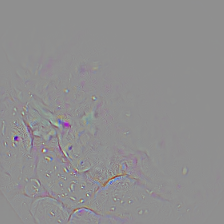

Supplement: Supplementary file 6 — Supplementary Data 5 [file 41467_2020_16777_MOESM6_ESM.gz › SupplementaryData5.255gradcam_heatmaps/IMG_01.201904250027.01.0018.1556158198.jpg_malignant_ggcam_densenet169_finetune.png]

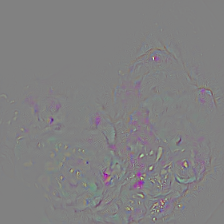

Supplement: Supplementary file 6 — Supplementary Data 5 [file 41467_2020_16777_MOESM6_ESM.gz › SupplementaryData5.255gradcam_heatmaps/IMG_01.201904240001.01.0014.1556064409.jpg_malignant_ggcam_densenet169_finetune.png]

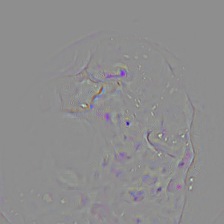

Supplement: Supplementary file 6 — Supplementary Data 5 [file 41467_2020_16777_MOESM6_ESM.gz › SupplementaryData5.255gradcam_heatmaps/IMG_01.201905150002.01.0011.1557878731.jpg_malignant_ggcam_densenet169_finetune.png]

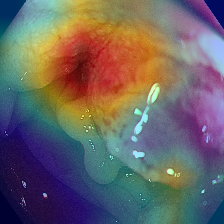

Supplement: Supplementary file 6 — Supplementary Data 5 [file 41467_2020_16777_MOESM6_ESM.gz › SupplementaryData5.255gradcam_heatmaps/IMG_01.201904220036.01.0018.1555899478.jpg_malignant_gcam_densenet169_finetune.png]

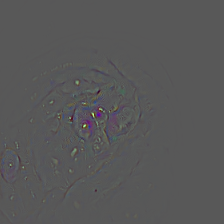

Supplement: Supplementary file 6 — Supplementary Data 5 [file 41467_2020_16777_MOESM6_ESM.gz › SupplementaryData5.255gradcam_heatmaps/IMG_01.201905210026.01.0021.1558402124.jpg_malignant_ggcam_densenet169_finetune.png]

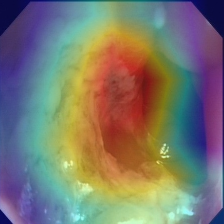

Supplement: Supplementary file 6 — Supplementary Data 5 [file 41467_2020_16777_MOESM6_ESM.gz › SupplementaryData5.255gradcam_heatmaps/IMG_01.201904290034.03.0006.1557811573.jpg_malignant_gcam_densenet169_finetune.png]

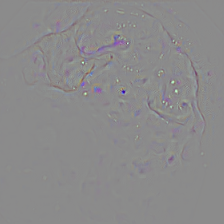

Supplement: Supplementary file 6 — Supplementary Data 5 [file 41467_2020_16777_MOESM6_ESM.gz › SupplementaryData5.255gradcam_heatmaps/IMG_01.201905150002.01.0010.1557878730.jpg_malignant_ggcam_densenet169_finetune.png]

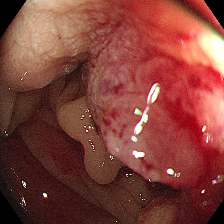

Supplement: Supplementary file 6 — Supplementary Data 5 [file 41467_2020_16777_MOESM6_ESM.gz › SupplementaryData5.255gradcam_heatmaps/IMG_01.201904220036.01.0018.1555899478.jpg_malignant_gcam_densenet169_finetune.png_raw_image.png]

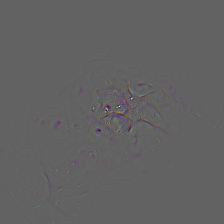

Supplement: Supplementary file 6 — Supplementary Data 5 [file 41467_2020_16777_MOESM6_ESM.gz › SupplementaryData5.255gradcam_heatmaps/IMG_01.201905160080.01.0005.1557987273.jpg_malignant_ggcam_densenet169_finetune.png]

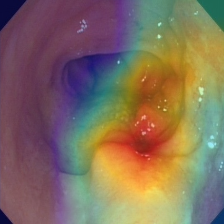

Supplement: Supplementary file 6 — Supplementary Data 5 [file 41467_2020_16777_MOESM6_ESM.gz › SupplementaryData5.255gradcam_heatmaps/IMG_01.201905130039.01.0092.1557714852.jpg_malignant_gcam_densenet169_finetune.png]

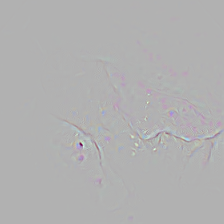

Supplement: Supplementary file 6 — Supplementary Data 5 [file 41467_2020_16777_MOESM6_ESM.gz › SupplementaryData5.255gradcam_heatmaps/IMG_01.201904150008.01.0004.1555287676.jpg_malignant_ggcam_densenet169_finetune.png]

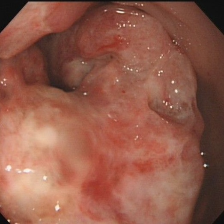

Supplement: Supplementary file 6 — Supplementary Data 5 [file 41467_2020_16777_MOESM6_ESM.gz › SupplementaryData5.255gradcam_heatmaps/IMG_01.201905150002.01.0010.1557878730.jpg_malignant_gcam_densenet169_finetune.png_raw_image.png]

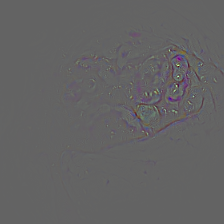

Supplement: Supplementary file 6 — Supplementary Data 5 [file 41467_2020_16777_MOESM6_ESM.gz › SupplementaryData5.255gradcam_heatmaps/IMG_01.201905210001.01.0022.1558396246.jpg_malignant_ggcam_densenet169_finetune.png]

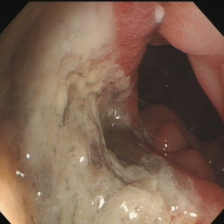

Supplement: Supplementary file 6 — Supplementary Data 5 [file 41467_2020_16777_MOESM6_ESM.gz › SupplementaryData5.255gradcam_heatmaps/IMG_01.201904290034.03.0007.1557811579.jpg_malignant_gcam_densenet169_finetune.png_raw_image.png]

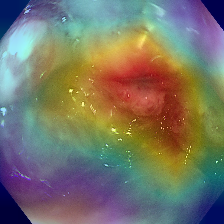

Supplement: Supplementary file 6 — Supplementary Data 5 [file 41467_2020_16777_MOESM6_ESM.gz › SupplementaryData5.255gradcam_heatmaps/IMG_01.201904220033.01.0005.1555898400.jpg_malignant_gcam_densenet169_finetune.png]

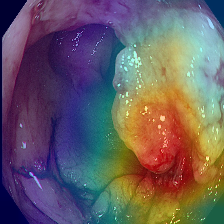

Supplement: Supplementary file 6 — Supplementary Data 5 [file 41467_2020_16777_MOESM6_ESM.gz › SupplementaryData5.255gradcam_heatmaps/IMG_01.201905100016.01.0049.1557451248.jpg_malignant_gcam_densenet169_finetune.png]

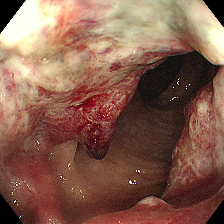

Supplement: Supplementary file 6 — Supplementary Data 5 [file 41467_2020_16777_MOESM6_ESM.gz › SupplementaryData5.255gradcam_heatmaps/IMG_01.201904220033.01.0030.1555898643.jpg_malignant_gcam_densenet169_finetune.png_raw_image.png]

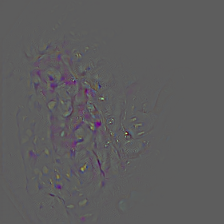

Supplement: Supplementary file 6 — Supplementary Data 5 [file 41467_2020_16777_MOESM6_ESM.gz › SupplementaryData5.255gradcam_heatmaps/IMG_01.201807160022.05.0056.1557360767.jpg_malignant_ggcam_densenet169_finetune.png]

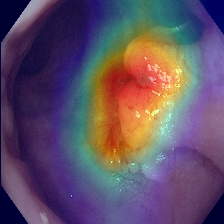

Supplement: Supplementary file 6 — Supplementary Data 5 [file 41467_2020_16777_MOESM6_ESM.gz › SupplementaryData5.255gradcam_heatmaps/IMG_01.201905090005.01.0003.1557361469.jpg_malignant_gcam_densenet169_finetune.png]

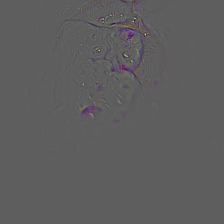

Supplement: Supplementary file 6 — Supplementary Data 5 [file 41467_2020_16777_MOESM6_ESM.gz › SupplementaryData5.255gradcam_heatmaps/IMG_01.201904230018.01.0005.1555982160.jpg_malignant_ggcam_densenet169_finetune.png]

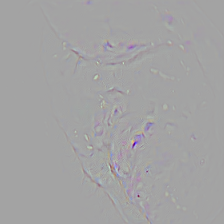

Supplement: Supplementary file 6 — Supplementary Data 5 [file 41467_2020_16777_MOESM6_ESM.gz › SupplementaryData5.255gradcam_heatmaps/IMG_01.201904240011.01.0005.1556069920.jpg_malignant_ggcam_densenet169_finetune.png]

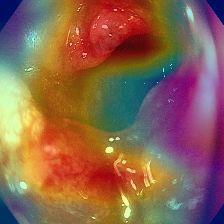

Supplement: Supplementary file 6 — Supplementary Data 5 [file 41467_2020_16777_MOESM6_ESM.gz › SupplementaryData5.255gradcam_heatmaps/IMG_01.201904120017.01.0111.1555034535.jpg_malignant_gcam_densenet169_finetune.png]

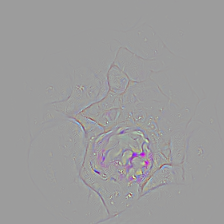

Supplement: Supplementary file 6 — Supplementary Data 5 [file 41467_2020_16777_MOESM6_ESM.gz › SupplementaryData5.255gradcam_heatmaps/IMG_01.201904250027.01.0001.1556158085.jpg_malignant_ggcam_densenet169_finetune.png]

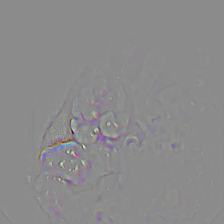

Supplement: Supplementary file 6 — Supplementary Data 5 [file 41467_2020_16777_MOESM6_ESM.gz › SupplementaryData5.255gradcam_heatmaps/IMG_01.201904160035.01.0017.1555381233.jpg_malignant_ggcam_densenet169_finetune.png]

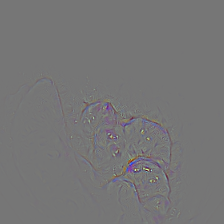

Supplement: Supplementary file 6 — Supplementary Data 5 [file 41467_2020_16777_MOESM6_ESM.gz › SupplementaryData5.255gradcam_heatmaps/IMG_01.201905230035.01.0004.1558577967.jpg_malignant_ggcam_densenet169_finetune.png]

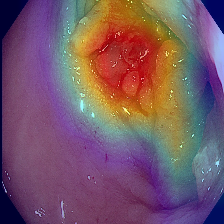

Supplement: Supplementary file 6 — Supplementary Data 5 [file 41467_2020_16777_MOESM6_ESM.gz › SupplementaryData5.255gradcam_heatmaps/IMG_01.201904010042.01.0008.1554087253.jpg_malignant_gcam_densenet169_finetune.png]

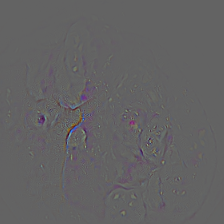

Supplement: Supplementary file 6 — Supplementary Data 5 [file 41467_2020_16777_MOESM6_ESM.gz › SupplementaryData5.255gradcam_heatmaps/IMG_01.201904150049.01.0090.1555299926.jpg_malignant_ggcam_densenet169_finetune.png]

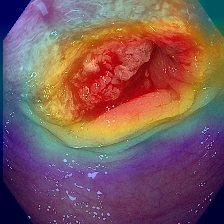

Supplement: Supplementary file 6 — Supplementary Data 5 [file 41467_2020_16777_MOESM6_ESM.gz › SupplementaryData5.255gradcam_heatmaps/IMG_01.201905230059.01.0008.1558581822.jpg_malignant_gcam_densenet169_finetune.png]

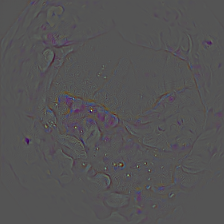

Supplement: Supplementary file 6 — Supplementary Data 5 [file 41467_2020_16777_MOESM6_ESM.gz › SupplementaryData5.255gradcam_heatmaps/IMG_01.201905080001.01.0027.1557272444.jpg_malignant_ggcam_densenet169_finetune.png]

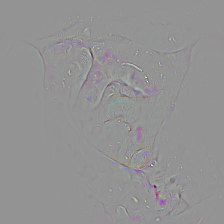

Supplement: Supplementary file 6 — Supplementary Data 5 [file 41467_2020_16777_MOESM6_ESM.gz › SupplementaryData5.255gradcam_heatmaps/IMG_01.201905100016.01.0011.1557450485.jpg_malignant_ggcam_densenet169_finetune.png]

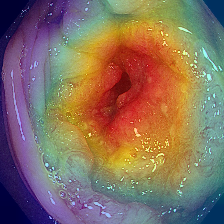

Supplement: Supplementary file 6 — Supplementary Data 5 [file 41467_2020_16777_MOESM6_ESM.gz › SupplementaryData5.255gradcam_heatmaps/IMG_01.201904230076.01.0020.1556001800.jpg_malignant_gcam_densenet169_finetune.png]

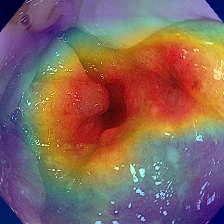

Supplement: Supplementary file 6 — Supplementary Data 5 [file 41467_2020_16777_MOESM6_ESM.gz › SupplementaryData5.255gradcam_heatmaps/IMG_01.201904230076.01.0016.1556001769.jpg_malignant_gcam_densenet169_finetune.png]

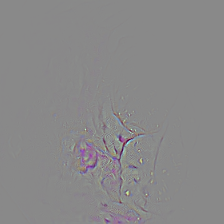

Supplement: Supplementary file 6 — Supplementary Data 5 [file 41467_2020_16777_MOESM6_ESM.gz › SupplementaryData5.255gradcam_heatmaps/IMG_01.201904250032.01.0012.1556159024.jpg_malignant_ggcam_densenet169_finetune.png]

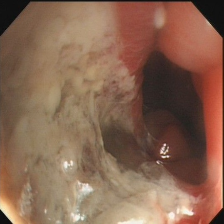

Supplement: Supplementary file 6 — Supplementary Data 5 [file 41467_2020_16777_MOESM6_ESM.gz › SupplementaryData5.255gradcam_heatmaps/IMG_01.201904290034.03.0006.1557811573.jpg_malignant_gcam_densenet169_finetune.png_raw_image.png]

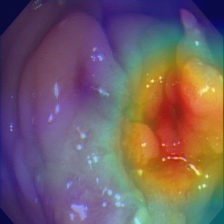

Supplement: Supplementary file 6 — Supplementary Data 5 [file 41467_2020_16777_MOESM6_ESM.gz › SupplementaryData5.255gradcam_heatmaps/IMG_01.201904150012.01.0029.1555292208.jpg_malignant_gcam_densenet169_finetune.png]

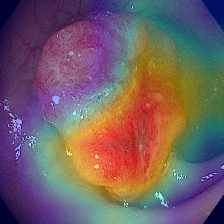

Supplement: Supplementary file 6 — Supplementary Data 5 [file 41467_2020_16777_MOESM6_ESM.gz › SupplementaryData5.255gradcam_heatmaps/IMG_01.201904020001.01.0011.1554162280.jpg_malignant_gcam_densenet169_finetune.png]

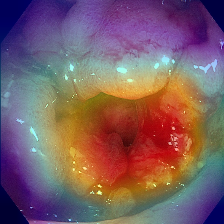

Supplement: Supplementary file 6 — Supplementary Data 5 [file 41467_2020_16777_MOESM6_ESM.gz › SupplementaryData5.255gradcam_heatmaps/IMG_01.201905210001.01.0011.1558396142.jpg_malignant_gcam_densenet169_finetune.png]

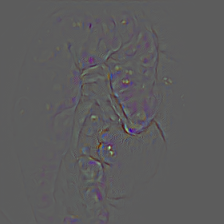

Supplement: Supplementary file 6 — Supplementary Data 5 [file 41467_2020_16777_MOESM6_ESM.gz › SupplementaryData5.255gradcam_heatmaps/IMG_01.201905100001.01.0004.1557444867.jpg_malignant_ggcam_densenet169_finetune.png]

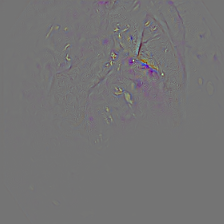

Supplement: Supplementary file 6 — Supplementary Data 5 [file 41467_2020_16777_MOESM6_ESM.gz › SupplementaryData5.255gradcam_heatmaps/IMG_01.201904010060.01.0013.1554100531.jpg_malignant_ggcam_densenet169_finetune.png]

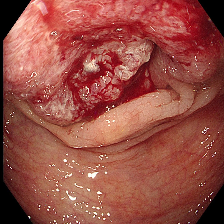

Supplement: Supplementary file 6 — Supplementary Data 5 [file 41467_2020_16777_MOESM6_ESM.gz › SupplementaryData5.255gradcam_heatmaps/IMG_01.201905230059.01.0008.1558581822.jpg_malignant_gcam_densenet169_finetune.png_raw_image.png]

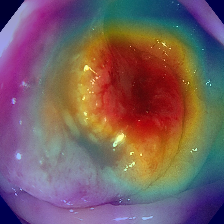

Supplement: Supplementary file 6 — Supplementary Data 5 [file 41467_2020_16777_MOESM6_ESM.gz › SupplementaryData5.255gradcam_heatmaps/IMG_01.201905150021.01.0018.1557885217.jpg_malignant_gcam_densenet169_finetune.png]

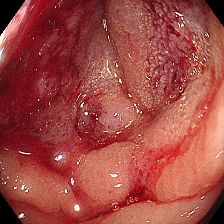

Supplement: Supplementary file 6 — Supplementary Data 5 [file 41467_2020_16777_MOESM6_ESM.gz › SupplementaryData5.255gradcam_heatmaps/IMG_01.201904280003.01.0021.1556409670.jpg_malignant_gcam_densenet169_finetune.png_raw_image.png]

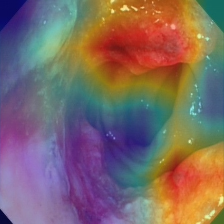

Supplement: Supplementary file 6 — Supplementary Data 5 [file 41467_2020_16777_MOESM6_ESM.gz › SupplementaryData5.255gradcam_heatmaps/IMG_01.201904110003.01.0093.1554941581.jpg_malignant_gcam_densenet169_finetune.png]

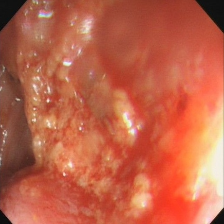

Supplement: Supplementary file 6 — Supplementary Data 5 [file 41467_2020_16777_MOESM6_ESM.gz › SupplementaryData5.255gradcam_heatmaps/IMG_01.201904230014.01.0017.1555980674.jpg_malignant_gcam_densenet169_finetune.png_raw_image.png]

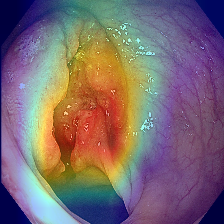

Supplement: Supplementary file 6 — Supplementary Data 5 [file 41467_2020_16777_MOESM6_ESM.gz › SupplementaryData5.255gradcam_heatmaps/IMG_01.201905090069.01.0061.1557383119.jpg_malignant_gcam_densenet169_finetune.png]

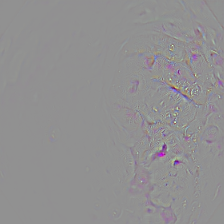

Supplement: Supplementary file 6 — Supplementary Data 5 [file 41467_2020_16777_MOESM6_ESM.gz › SupplementaryData5.255gradcam_heatmaps/IMG_01.201905150021.01.0005.1557885117.jpg_malignant_ggcam_densenet169_finetune.png]

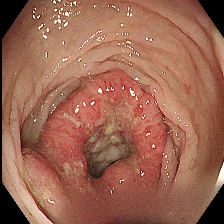

Supplement: Supplementary file 6 — Supplementary Data 5 [file 41467_2020_16777_MOESM6_ESM.gz › SupplementaryData5.255gradcam_heatmaps/IMG_01.201903210020.03.0002.1554875270.jpg_malignant_gcam_densenet169_finetune.png_raw_image.png]

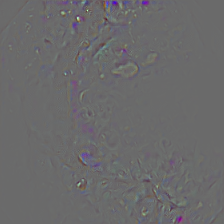

Supplement: Supplementary file 6 — Supplementary Data 5 [file 41467_2020_16777_MOESM6_ESM.gz › SupplementaryData5.255gradcam_heatmaps/IMG_01.201905160036.02.0012.1558315015.jpg_malignant_ggcam_densenet169_finetune.png]

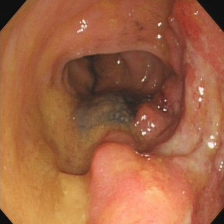

Supplement: Supplementary file 6 — Supplementary Data 5 [file 41467_2020_16777_MOESM6_ESM.gz › SupplementaryData5.255gradcam_heatmaps/IMG_01.201905130039.01.0092.1557714852.jpg_malignant_gcam_densenet169_finetune.png_raw_image.png]

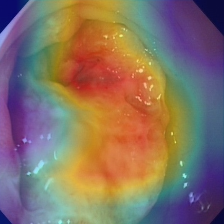

Supplement: Supplementary file 6 — Supplementary Data 5 [file 41467_2020_16777_MOESM6_ESM.gz › SupplementaryData5.255gradcam_heatmaps/IMG_01.201905150002.01.0009.1557878727.jpg_malignant_gcam_densenet169_finetune.png]

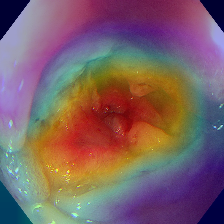

Supplement: Supplementary file 6 — Supplementary Data 5 [file 41467_2020_16777_MOESM6_ESM.gz › SupplementaryData5.255gradcam_heatmaps/IMG_01.201905160080.01.0005.1557987273.jpg_malignant_gcam_densenet169_finetune.png]

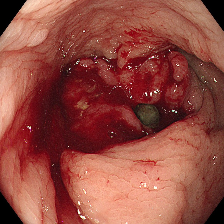

Supplement: Supplementary file 6 — Supplementary Data 5 [file 41467_2020_16777_MOESM6_ESM.gz › SupplementaryData5.255gradcam_heatmaps/IMG_01.201905210001.01.0022.1558396246.jpg_malignant_gcam_densenet169_finetune.png_raw_image.png]

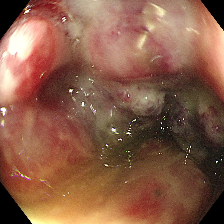

Supplement: Supplementary file 6 — Supplementary Data 5 [file 41467_2020_16777_MOESM6_ESM.gz › SupplementaryData5.255gradcam_heatmaps/IMG_01.201904220033.01.0005.1555898400.jpg_malignant_gcam_densenet169_finetune.png_raw_image.png]

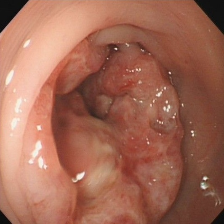

Supplement: Supplementary file 6 — Supplementary Data 5 [file 41467_2020_16777_MOESM6_ESM.gz › SupplementaryData5.255gradcam_heatmaps/IMG_01.201905150002.01.0017.1557878757.jpg_malignant_gcam_densenet169_finetune.png_raw_image.png]

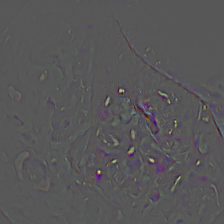

Supplement: Supplementary file 6 — Supplementary Data 5 [file 41467_2020_16777_MOESM6_ESM.gz › SupplementaryData5.255gradcam_heatmaps/IMG_01.201904080028.01.0010.1554688411.jpg_malignant_ggcam_densenet169_finetune.png]

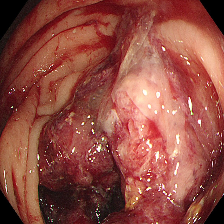

Supplement: Supplementary file 6 — Supplementary Data 5 [file 41467_2020_16777_MOESM6_ESM.gz › SupplementaryData5.255gradcam_heatmaps/IMG_01.201905070002.01.0016.1557187720.jpg_malignant_gcam_densenet169_finetune.png_raw_image.png]

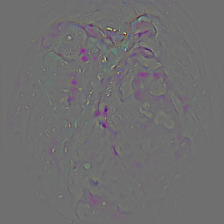

Supplement: Supplementary file 6 — Supplementary Data 5 [file 41467_2020_16777_MOESM6_ESM.gz › SupplementaryData5.255gradcam_heatmaps/IMG_01.201905100027.01.0010.1557453154.jpg_malignant_ggcam_densenet169_finetune.png]

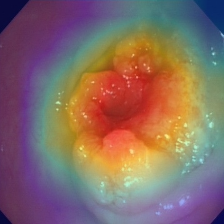

Supplement: Supplementary file 6 — Supplementary Data 5 [file 41467_2020_16777_MOESM6_ESM.gz › SupplementaryData5.255gradcam_heatmaps/IMG_01.201904160035.01.0027.1555381246.jpg_malignant_gcam_densenet169_finetune.png]

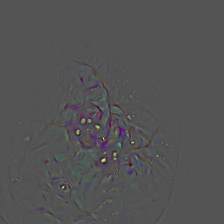

Supplement: Supplementary file 6 — Supplementary Data 5 [file 41467_2020_16777_MOESM6_ESM.gz › SupplementaryData5.255gradcam_heatmaps/IMG_01.201904030029.01.0024.1554255594.jpg_malignant_ggcam_densenet169_finetune.png]

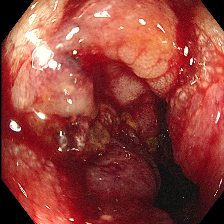

Supplement: Supplementary file 6 — Supplementary Data 5 [file 41467_2020_16777_MOESM6_ESM.gz › SupplementaryData5.255gradcam_heatmaps/IMG_01.201904120017.01.0114.1555034767.jpg_malignant_gcam_densenet169_finetune.png_raw_image.png]

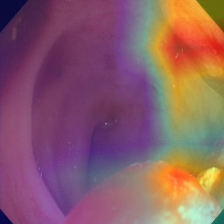

Supplement: Supplementary file 6 — Supplementary Data 5 [file 41467_2020_16777_MOESM6_ESM.gz › SupplementaryData5.255gradcam_heatmaps/IMG_01.201905200027.01.0056.1558316335.jpg_malignant_gcam_densenet169_finetune.png]

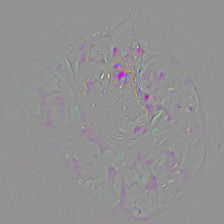

Supplement: Supplementary file 6 — Supplementary Data 5 [file 41467_2020_16777_MOESM6_ESM.gz › SupplementaryData5.255gradcam_heatmaps/IMG_01.201904170017.01.0042.1555465038.jpg_malignant_ggcam_densenet169_finetune.png]

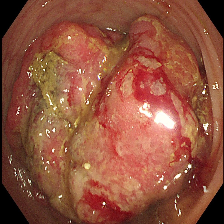

Supplement: Supplementary file 6 — Supplementary Data 5 [file 41467_2020_16777_MOESM6_ESM.gz › SupplementaryData5.255gradcam_heatmaps/IMG_01.201905100027.01.0010.1557453154.jpg_malignant_gcam_densenet169_finetune.png_raw_image.png]

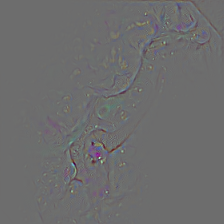

Supplement: Supplementary file 6 — Supplementary Data 5 [file 41467_2020_16777_MOESM6_ESM.gz › SupplementaryData5.255gradcam_heatmaps/IMG_01.201905150037.01.0007.1557898305.jpg_malignant_ggcam_densenet169_finetune.png]

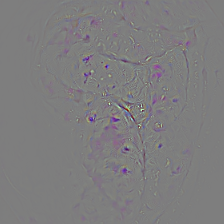

Supplement: Supplementary file 6 — Supplementary Data 5 [file 41467_2020_16777_MOESM6_ESM.gz › SupplementaryData5.255gradcam_heatmaps/IMG_01.201905150021.01.0013.1557885167.jpg_malignant_ggcam_densenet169_finetune.png]

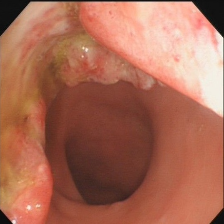

Supplement: Supplementary file 6 — Supplementary Data 5 [file 41467_2020_16777_MOESM6_ESM.gz › SupplementaryData5.255gradcam_heatmaps/IMG_01.201905100001.01.0040.1557445581.jpg_malignant_gcam_densenet169_finetune.png_raw_image.png]

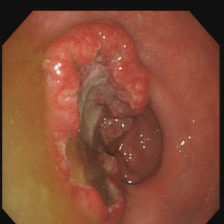

Supplement: Supplementary file 6 — Supplementary Data 5 [file 41467_2020_16777_MOESM6_ESM.gz › SupplementaryData5.255gradcam_heatmaps/IMG_01.201905100001.01.0001.1557444843.jpg_malignant_gcam_densenet169_finetune.png_raw_image.png]

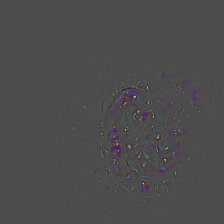

Supplement: Supplementary file 6 — Supplementary Data 5 [file 41467_2020_16777_MOESM6_ESM.gz › SupplementaryData5.255gradcam_heatmaps/IMG_01.201905100027.01.0019.1557453531.jpg_malignant_ggcam_densenet169_finetune.png]

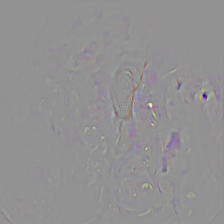

Supplement: Supplementary file 6 — Supplementary Data 5 [file 41467_2020_16777_MOESM6_ESM.gz › SupplementaryData5.255gradcam_heatmaps/IMG_01.201905140036.01.0006.1557800457.jpg_malignant_ggcam_densenet169_finetune.png]

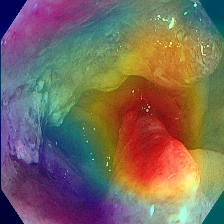

Supplement: Supplementary file 6 — Supplementary Data 5 [file 41467_2020_16777_MOESM6_ESM.gz › SupplementaryData5.255gradcam_heatmaps/IMG_01.201904150049.01.0010.1555299093.jpg_malignant_gcam_densenet169_finetune.png]

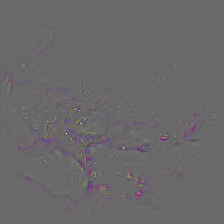

Supplement: Supplementary file 6 — Supplementary Data 5 [file 41467_2020_16777_MOESM6_ESM.gz › SupplementaryData5.255gradcam_heatmaps/IMG_01.201904280003.01.0023.1556409677.jpg_malignant_ggcam_densenet169_finetune.png]

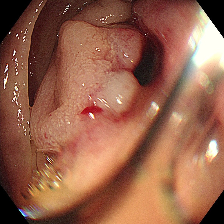

Supplement: Supplementary file 6 — Supplementary Data 5 [file 41467_2020_16777_MOESM6_ESM.gz › SupplementaryData5.255gradcam_heatmaps/IMG_01.201904230018.01.0005.1555982160.jpg_malignant_gcam_densenet169_finetune.png_raw_image.png]

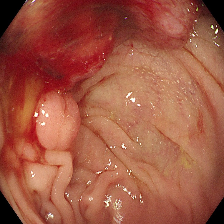

Supplement: Supplementary file 6 — Supplementary Data 5 [file 41467_2020_16777_MOESM6_ESM.gz › SupplementaryData5.255gradcam_heatmaps/IMG_01.201904300043.02.0074.1557122240.jpg_malignant_gcam_densenet169_finetune.png_raw_image.png]

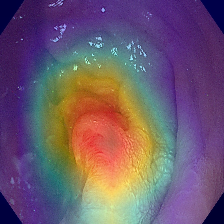

Supplement: Supplementary file 6 — Supplementary Data 5 [file 41467_2020_16777_MOESM6_ESM.gz › SupplementaryData5.255gradcam_heatmaps/IMG_01.201904150033.01.0001.1555294393.jpg_malignant_gcam_densenet169_finetune.png]

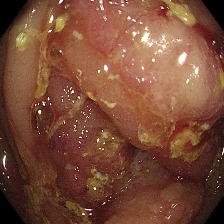

Supplement: Supplementary file 6 — Supplementary Data 5 [file 41467_2020_16777_MOESM6_ESM.gz › SupplementaryData5.255gradcam_heatmaps/IMG_01.201905090036.01.0001.1557368261.jpg_malignant_gcam_densenet169_finetune.png_raw_image.png]

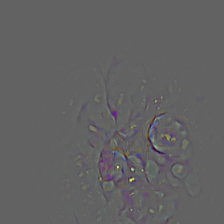

Supplement: Supplementary file 6 — Supplementary Data 5 [file 41467_2020_16777_MOESM6_ESM.gz › SupplementaryData5.255gradcam_heatmaps/IMG_01.201904150012.01.0033.1555292256.jpg_malignant_ggcam_densenet169_finetune.png]

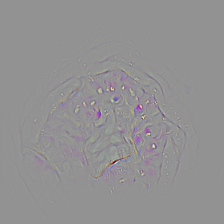

Supplement: Supplementary file 6 — Supplementary Data 5 [file 41467_2020_16777_MOESM6_ESM.gz › SupplementaryData5.255gradcam_heatmaps/IMG_01.201903210020.03.0002.1554875270.jpg_malignant_ggcam_densenet169_finetune.png]
