# Supplementary figures and images for: Diagnostic evaluation of a deep learning model for optical diagnosis of colorectal cancer (part 5 of 5)
Source: Nat Commun. 2020 Jun 11;11:2961. doi: 10.1038/s41467-020-16777-6 (PMC7289893; doi:10.1038/s41467-020-16777-6)

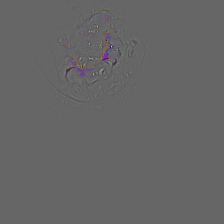

Supplement: Supplementary file 6 — Supplementary Data 5 [file 41467_2020_16777_MOESM6_ESM.gz › SupplementaryData5.255gradcam_heatmaps/IMG_01.201904150033.01.0039.1555294534.jpg_malignant_ggcam_densenet169_finetune.png]

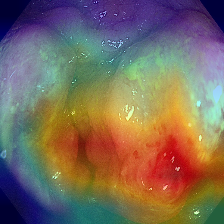

Supplement: Supplementary file 6 — Supplementary Data 5 [file 41467_2020_16777_MOESM6_ESM.gz › SupplementaryData5.255gradcam_heatmaps/IMG_01.201904170017.01.0038.1555465003.jpg_malignant_gcam_densenet169_finetune.png]
