# Supplementary figures and images for: Diagnostic evaluation of a deep learning model for optical diagnosis of colorectal cancer (part 4 of 5)
Source: Nat Commun. 2020 Jun 11;11:2961. doi: 10.1038/s41467-020-16777-6 (PMC7289893; doi:10.1038/s41467-020-16777-6)

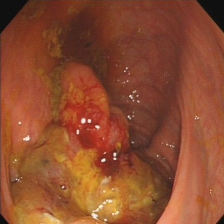

Supplement: Supplementary file 6 — Supplementary Data 5 [file 41467_2020_16777_MOESM6_ESM.gz › SupplementaryData5.255gradcam_heatmaps/IMG_01.201904030029.01.0024.1554255594.jpg_malignant_gcam_densenet169_finetune.png_raw_image.png]

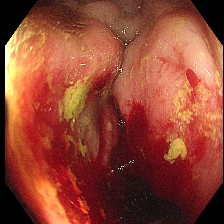

Supplement: Supplementary file 6 — Supplementary Data 5 [file 41467_2020_16777_MOESM6_ESM.gz › SupplementaryData5.255gradcam_heatmaps/IMG_01.201904170017.01.0045.1555465076.jpg_malignant_gcam_densenet169_finetune.png_raw_image.png]

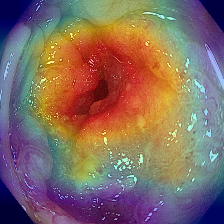

Supplement: Supplementary file 6 — Supplementary Data 5 [file 41467_2020_16777_MOESM6_ESM.gz › SupplementaryData5.255gradcam_heatmaps/IMG_01.201904230076.01.0018.1556001785.jpg_malignant_gcam_densenet169_finetune.png]

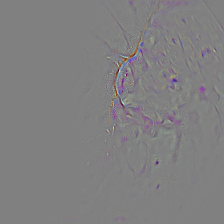

Supplement: Supplementary file 6 — Supplementary Data 5 [file 41467_2020_16777_MOESM6_ESM.gz › SupplementaryData5.255gradcam_heatmaps/IMG_01.201905090079.01.0131.1557387129.jpg_malignant_ggcam_densenet169_finetune.png]

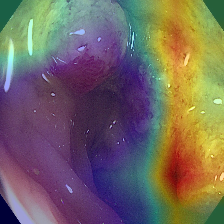

Supplement: Supplementary file 6 — Supplementary Data 5 [file 41467_2020_16777_MOESM6_ESM.gz › SupplementaryData5.255gradcam_heatmaps/IMG_01.201904080006.01.0006.1554682628.jpg_malignant_gcam_densenet169_finetune.png]

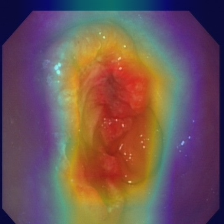

Supplement: Supplementary file 6 — Supplementary Data 5 [file 41467_2020_16777_MOESM6_ESM.gz › SupplementaryData5.255gradcam_heatmaps/IMG_01.201905100001.01.0001.1557444843.jpg_malignant_gcam_densenet169_finetune.png]

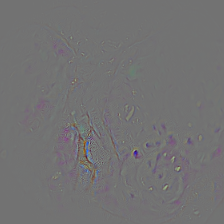

Supplement: Supplementary file 6 — Supplementary Data 5 [file 41467_2020_16777_MOESM6_ESM.gz › SupplementaryData5.255gradcam_heatmaps/IMG_01.201904170017.01.0038.1555465003.jpg_malignant_ggcam_densenet169_finetune.png]

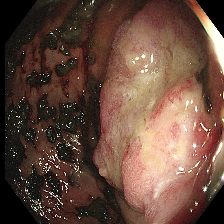

Supplement: Supplementary file 6 — Supplementary Data 5 [file 41467_2020_16777_MOESM6_ESM.gz › SupplementaryData5.255gradcam_heatmaps/IMG_01.201905160024.01.0009.1557970354.jpg_malignant_gcam_densenet169_finetune.png_raw_image.png]

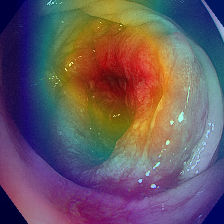

Supplement: Supplementary file 6 — Supplementary Data 5 [file 41467_2020_16777_MOESM6_ESM.gz › SupplementaryData5.255gradcam_heatmaps/IMG_01.201904220033.01.0037.1555898661.jpg_malignant_gcam_densenet169_finetune.png]

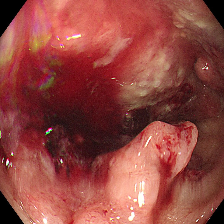

Supplement: Supplementary file 6 — Supplementary Data 5 [file 41467_2020_16777_MOESM6_ESM.gz › SupplementaryData5.255gradcam_heatmaps/IMG_01.201905160002.01.0027.1557964758.jpg_malignant_gcam_densenet169_finetune.png_raw_image.png]

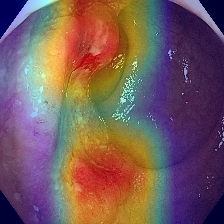

Supplement: Supplementary file 6 — Supplementary Data 5 [file 41467_2020_16777_MOESM6_ESM.gz › SupplementaryData5.255gradcam_heatmaps/IMG_01.201903220015.02.0010.1554078594.jpg_malignant_gcam_densenet169_finetune.png]

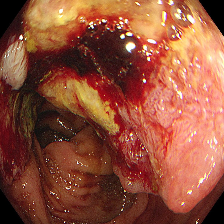

Supplement: Supplementary file 6 — Supplementary Data 5 [file 41467_2020_16777_MOESM6_ESM.gz › SupplementaryData5.255gradcam_heatmaps/IMG_01.201905200019.01.0020.1558315574.jpg_malignant_gcam_densenet169_finetune.png_raw_image.png]

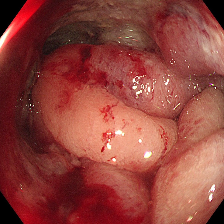

Supplement: Supplementary file 6 — Supplementary Data 5 [file 41467_2020_16777_MOESM6_ESM.gz › SupplementaryData5.255gradcam_heatmaps/IMG_01.201905150021.01.0020.1557885289.jpg_malignant_gcam_densenet169_finetune.png_raw_image.png]

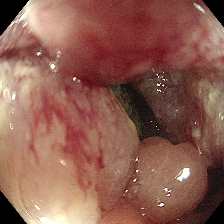

Supplement: Supplementary file 6 — Supplementary Data 5 [file 41467_2020_16777_MOESM6_ESM.gz › SupplementaryData5.255gradcam_heatmaps/IMG_01.201904250027.01.0014.1556158143.jpg_malignant_gcam_densenet169_finetune.png_raw_image.png]

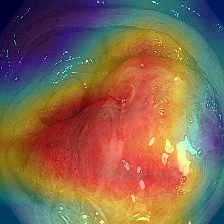

Supplement: Supplementary file 6 — Supplementary Data 5 [file 41467_2020_16777_MOESM6_ESM.gz › SupplementaryData5.255gradcam_heatmaps/IMG_01.201904240016.01.0011.1556067781.jpg_malignant_gcam_densenet169_finetune.png]

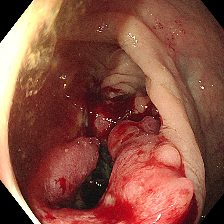

Supplement: Supplementary file 6 — Supplementary Data 5 [file 41467_2020_16777_MOESM6_ESM.gz › SupplementaryData5.255gradcam_heatmaps/IMG_01.201904220033.01.0040.1555898830.jpg_malignant_gcam_densenet169_finetune.png_raw_image.png]

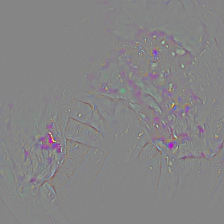

Supplement: Supplementary file 6 — Supplementary Data 5 [file 41467_2020_16777_MOESM6_ESM.gz › SupplementaryData5.255gradcam_heatmaps/IMG_01.201905200019.01.0016.1558315513.jpg_malignant_ggcam_densenet169_finetune.png]

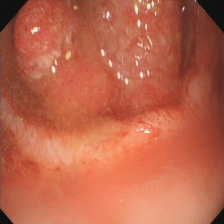

Supplement: Supplementary file 6 — Supplementary Data 5 [file 41467_2020_16777_MOESM6_ESM.gz › SupplementaryData5.255gradcam_heatmaps/IMG_01.201905200027.01.0003.1558315651.jpg_malignant_gcam_densenet169_finetune.png_raw_image.png]

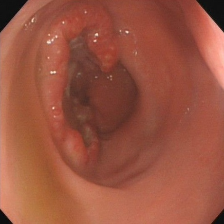

Supplement: Supplementary file 6 — Supplementary Data 5 [file 41467_2020_16777_MOESM6_ESM.gz › SupplementaryData5.255gradcam_heatmaps/IMG_01.201905100001.01.0003.1557444865.jpg_malignant_gcam_densenet169_finetune.png_raw_image.png]

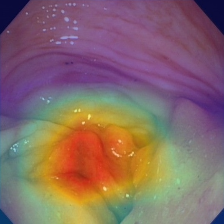

Supplement: Supplementary file 6 — Supplementary Data 5 [file 41467_2020_16777_MOESM6_ESM.gz › SupplementaryData5.255gradcam_heatmaps/IMG_01.201905210026.01.0020.1558401934.jpg_malignant_gcam_densenet169_finetune.png]

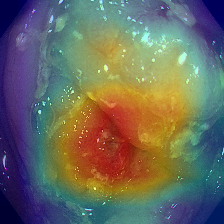

Supplement: Supplementary file 6 — Supplementary Data 5 [file 41467_2020_16777_MOESM6_ESM.gz › SupplementaryData5.255gradcam_heatmaps/IMG_01.201905090036.01.0001.1557368261.jpg_malignant_gcam_densenet169_finetune.png]

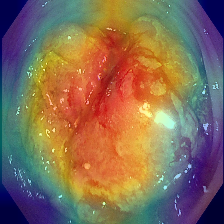

Supplement: Supplementary file 6 — Supplementary Data 5 [file 41467_2020_16777_MOESM6_ESM.gz › SupplementaryData5.255gradcam_heatmaps/IMG_01.201905100027.01.0010.1557453154.jpg_malignant_gcam_densenet169_finetune.png]

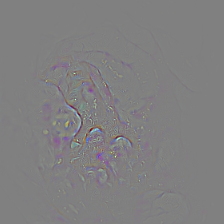

Supplement: Supplementary file 6 — Supplementary Data 5 [file 41467_2020_16777_MOESM6_ESM.gz › SupplementaryData5.255gradcam_heatmaps/IMG_01.201905230001.01.0012.1558570313.jpg_malignant_ggcam_densenet169_finetune.png]

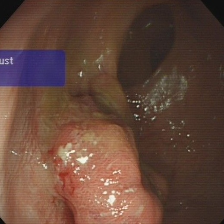

Supplement: Supplementary file 6 — Supplementary Data 5 [file 41467_2020_16777_MOESM6_ESM.gz › SupplementaryData5.255gradcam_heatmaps/IMG_01.201904010015.01.0006.1554079164.jpg_malignant_gcam_densenet169_finetune.png_raw_image.png]

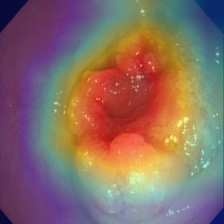

Supplement: Supplementary file 6 — Supplementary Data 5 [file 41467_2020_16777_MOESM6_ESM.gz › SupplementaryData5.255gradcam_heatmaps/IMG_01.201904160035.01.0025.1555381243.jpg_malignant_gcam_densenet169_finetune.png]

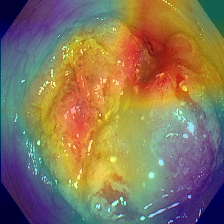

Supplement: Supplementary file 6 — Supplementary Data 5 [file 41467_2020_16777_MOESM6_ESM.gz › SupplementaryData5.255gradcam_heatmaps/IMG_01.201905100027.01.0011.1557453166.jpg_malignant_gcam_densenet169_finetune.png]

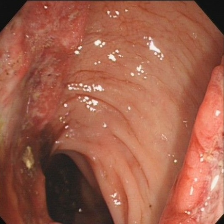

Supplement: Supplementary file 6 — Supplementary Data 5 [file 41467_2020_16777_MOESM6_ESM.gz › SupplementaryData5.255gradcam_heatmaps/IMG_01.201904020016.01.0031.1554166420.jpg_malignant_gcam_densenet169_finetune.png_raw_image.png]

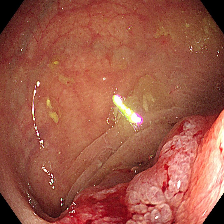

Supplement: Supplementary file 6 — Supplementary Data 5 [file 41467_2020_16777_MOESM6_ESM.gz › SupplementaryData5.255gradcam_heatmaps/IMG_01.201904010053.01.0010.1554088610.jpg_malignant_gcam_densenet169_finetune.png_raw_image.png]

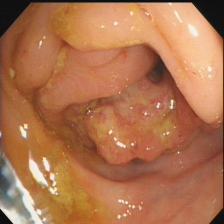

Supplement: Supplementary file 6 — Supplementary Data 5 [file 41467_2020_16777_MOESM6_ESM.gz › SupplementaryData5.255gradcam_heatmaps/IMG_01.201904240015.01.0006.1556066383.jpg_malignant_gcam_densenet169_finetune.png_raw_image.png]

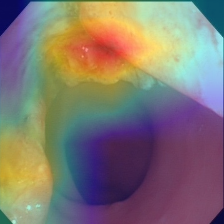

Supplement: Supplementary file 6 — Supplementary Data 5 [file 41467_2020_16777_MOESM6_ESM.gz › SupplementaryData5.255gradcam_heatmaps/IMG_01.201905100001.01.0040.1557445581.jpg_malignant_gcam_densenet169_finetune.png]

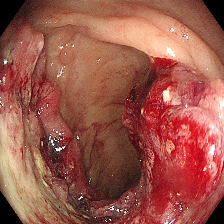

Supplement: Supplementary file 6 — Supplementary Data 5 [file 41467_2020_16777_MOESM6_ESM.gz › SupplementaryData5.255gradcam_heatmaps/IMG_01.201904290044.01.0081.1556507843.jpg_malignant_gcam_densenet169_finetune.png_raw_image.png]

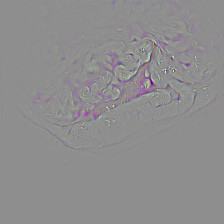

Supplement: Supplementary file 6 — Supplementary Data 5 [file 41467_2020_16777_MOESM6_ESM.gz › SupplementaryData5.255gradcam_heatmaps/IMG_01.201905230059.01.0008.1558581822.jpg_malignant_ggcam_densenet169_finetune.png]

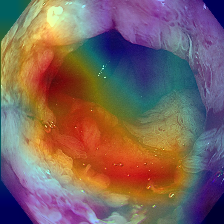

Supplement: Supplementary file 6 — Supplementary Data 5 [file 41467_2020_16777_MOESM6_ESM.gz › SupplementaryData5.255gradcam_heatmaps/IMG_01.201905080001.01.0027.1557272444.jpg_malignant_gcam_densenet169_finetune.png]

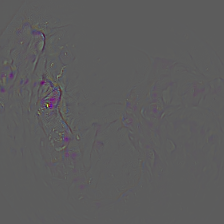

Supplement: Supplementary file 6 — Supplementary Data 5 [file 41467_2020_16777_MOESM6_ESM.gz › SupplementaryData5.255gradcam_heatmaps/IMG_01.201904290044.01.0081.1556507843.jpg_malignant_ggcam_densenet169_finetune.png]

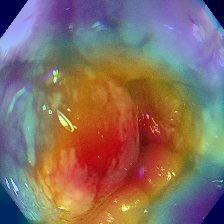

Supplement: Supplementary file 6 — Supplementary Data 5 [file 41467_2020_16777_MOESM6_ESM.gz › SupplementaryData5.255gradcam_heatmaps/IMG_01.201904250027.01.0016.1556158163.jpg_malignant_gcam_densenet169_finetune.png]

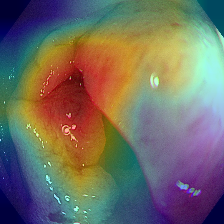

Supplement: Supplementary file 6 — Supplementary Data 5 [file 41467_2020_16777_MOESM6_ESM.gz › SupplementaryData5.255gradcam_heatmaps/IMG_01.201904230018.01.0004.1555982159.jpg_malignant_gcam_densenet169_finetune.png]

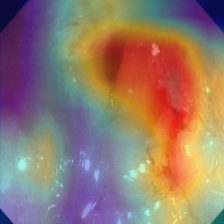

Supplement: Supplementary file 6 — Supplementary Data 5 [file 41467_2020_16777_MOESM6_ESM.gz › SupplementaryData5.255gradcam_heatmaps/IMG_01.201905140036.01.0011.1557800496.jpg_malignant_gcam_densenet169_finetune.png]

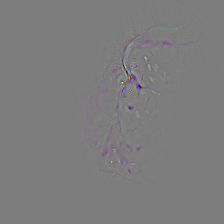

Supplement: Supplementary file 6 — Supplementary Data 5 [file 41467_2020_16777_MOESM6_ESM.gz › SupplementaryData5.255gradcam_heatmaps/IMG_01.201905090005.01.0004.1557361469.jpg_malignant_ggcam_densenet169_finetune.png]

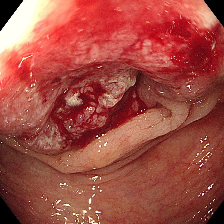

Supplement: Supplementary file 6 — Supplementary Data 5 [file 41467_2020_16777_MOESM6_ESM.gz › SupplementaryData5.255gradcam_heatmaps/IMG_01.201905230059.01.0009.1558581824.jpg_malignant_gcam_densenet169_finetune.png_raw_image.png]

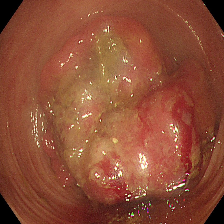

Supplement: Supplementary file 6 — Supplementary Data 5 [file 41467_2020_16777_MOESM6_ESM.gz › SupplementaryData5.255gradcam_heatmaps/IMG_01.201905100027.01.0009.1557453148.jpg_malignant_gcam_densenet169_finetune.png_raw_image.png]

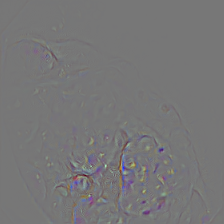

Supplement: Supplementary file 6 — Supplementary Data 5 [file 41467_2020_16777_MOESM6_ESM.gz › SupplementaryData5.255gradcam_heatmaps/IMG_01.201905160036.02.0006.1558314999.jpg_malignant_ggcam_densenet169_finetune.png]

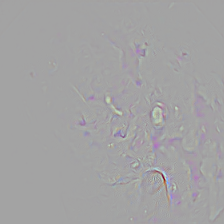

Supplement: Supplementary file 6 — Supplementary Data 5 [file 41467_2020_16777_MOESM6_ESM.gz › SupplementaryData5.255gradcam_heatmaps/IMG_01.201905080063.01.0003.1557285834.jpg_malignant_ggcam_densenet169_finetune.png]

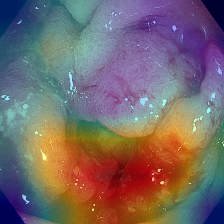

Supplement: Supplementary file 6 — Supplementary Data 5 [file 41467_2020_16777_MOESM6_ESM.gz › SupplementaryData5.255gradcam_heatmaps/IMG_01.201905210001.01.0013.1558396159.jpg_malignant_gcam_densenet169_finetune.png]

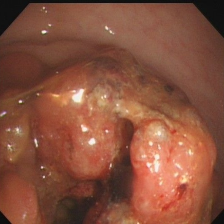

Supplement: Supplementary file 6 — Supplementary Data 5 [file 41467_2020_16777_MOESM6_ESM.gz › SupplementaryData5.255gradcam_heatmaps/IMG_01.201905160036.02.0007.1558315005.jpg_malignant_gcam_densenet169_finetune.png_raw_image.png]

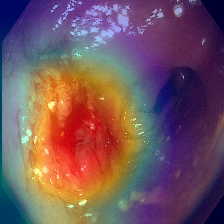

Supplement: Supplementary file 6 — Supplementary Data 5 [file 41467_2020_16777_MOESM6_ESM.gz › SupplementaryData5.255gradcam_heatmaps/IMG_01.201807160022.05.0056.1557360767.jpg_malignant_gcam_densenet169_finetune.png]

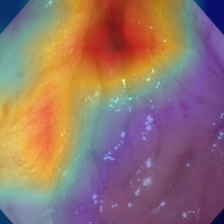

Supplement: Supplementary file 6 — Supplementary Data 5 [file 41467_2020_16777_MOESM6_ESM.gz › SupplementaryData5.255gradcam_heatmaps/IMG_01.201905140036.01.0003.1557800441.jpg_malignant_gcam_densenet169_finetune.png]

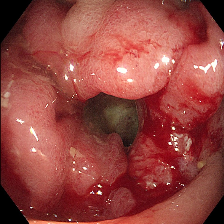

Supplement: Supplementary file 6 — Supplementary Data 5 [file 41467_2020_16777_MOESM6_ESM.gz › SupplementaryData5.255gradcam_heatmaps/IMG_01.201905210001.01.0011.1558396142.jpg_malignant_gcam_densenet169_finetune.png_raw_image.png]

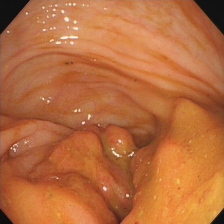

Supplement: Supplementary file 6 — Supplementary Data 5 [file 41467_2020_16777_MOESM6_ESM.gz › SupplementaryData5.255gradcam_heatmaps/IMG_01.201905210026.01.0020.1558401934.jpg_malignant_gcam_densenet169_finetune.png_raw_image.png]

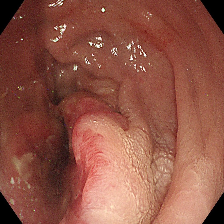

Supplement: Supplementary file 6 — Supplementary Data 5 [file 41467_2020_16777_MOESM6_ESM.gz › SupplementaryData5.255gradcam_heatmaps/IMG_01.201904150033.01.0001.1555294393.jpg_malignant_gcam_densenet169_finetune.png_raw_image.png]

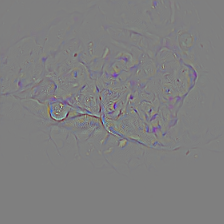

Supplement: Supplementary file 6 — Supplementary Data 5 [file 41467_2020_16777_MOESM6_ESM.gz › SupplementaryData5.255gradcam_heatmaps/00000004.jpg_malignant_ggcam_densenet169_finetune.png]

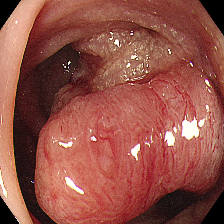

Supplement: Supplementary file 6 — Supplementary Data 5 [file 41467_2020_16777_MOESM6_ESM.gz › SupplementaryData5.255gradcam_heatmaps/IMG_01.201904010001.01.0049.1554076524.jpg_malignant_gcam_densenet169_finetune.png_raw_image.png]

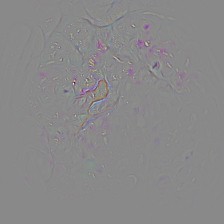

Supplement: Supplementary file 6 — Supplementary Data 5 [file 41467_2020_16777_MOESM6_ESM.gz › SupplementaryData5.255gradcam_heatmaps/IMG_01.201904230076.01.0018.1556001785.jpg_malignant_ggcam_densenet169_finetune.png]

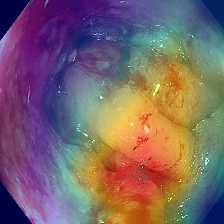

Supplement: Supplementary file 6 — Supplementary Data 5 [file 41467_2020_16777_MOESM6_ESM.gz › SupplementaryData5.255gradcam_heatmaps/IMG_01.201905150021.01.0016.1557885206.jpg_malignant_gcam_densenet169_finetune.png]

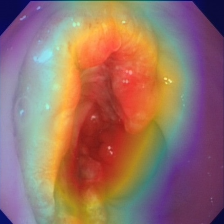

Supplement: Supplementary file 6 — Supplementary Data 5 [file 41467_2020_16777_MOESM6_ESM.gz › SupplementaryData5.255gradcam_heatmaps/IMG_01.201905100001.01.0004.1557444867.jpg_malignant_gcam_densenet169_finetune.png]

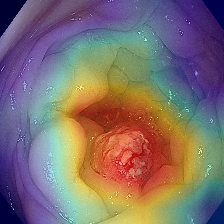

Supplement: Supplementary file 6 — Supplementary Data 5 [file 41467_2020_16777_MOESM6_ESM.gz › SupplementaryData5.255gradcam_heatmaps/IMG_01.201904250027.01.0001.1556158085.jpg_malignant_gcam_densenet169_finetune.png]

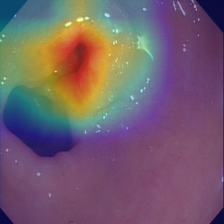

Supplement: Supplementary file 6 — Supplementary Data 5 [file 41467_2020_16777_MOESM6_ESM.gz › SupplementaryData5.255gradcam_heatmaps/IMG_01.201904020042.01.0008.1554170054.jpg_malignant_gcam_densenet169_finetune.png]

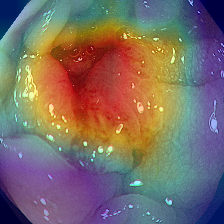

Supplement: Supplementary file 6 — Supplementary Data 5 [file 41467_2020_16777_MOESM6_ESM.gz › SupplementaryData5.255gradcam_heatmaps/IMG_01.201904230076.01.0010.1556001713.jpg_malignant_gcam_densenet169_finetune.png]

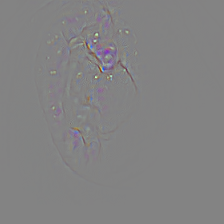

Supplement: Supplementary file 6 — Supplementary Data 5 [file 41467_2020_16777_MOESM6_ESM.gz › SupplementaryData5.255gradcam_heatmaps/IMG_01.201905100001.01.0003.1557444865.jpg_malignant_ggcam_densenet169_finetune.png]

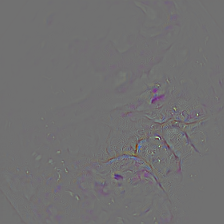

Supplement: Supplementary file 6 — Supplementary Data 5 [file 41467_2020_16777_MOESM6_ESM.gz › SupplementaryData5.255gradcam_heatmaps/IMG_01.201905160002.01.0029.1557964764.jpg_malignant_ggcam_densenet169_finetune.png]

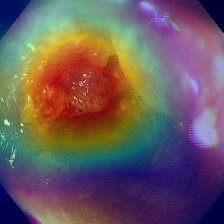

Supplement: Supplementary file 6 — Supplementary Data 5 [file 41467_2020_16777_MOESM6_ESM.gz › SupplementaryData5.255gradcam_heatmaps/IMG_01.201904240001.01.0008.1556064328.jpg_malignant_gcam_densenet169_finetune.png]

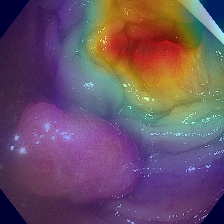

Supplement: Supplementary file 6 — Supplementary Data 5 [file 41467_2020_16777_MOESM6_ESM.gz › SupplementaryData5.255gradcam_heatmaps/IMG_01.201905200051.01.0005.1558330880.jpg_malignant_gcam_densenet169_finetune.png]

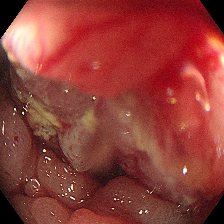

Supplement: Supplementary file 6 — Supplementary Data 5 [file 41467_2020_16777_MOESM6_ESM.gz › SupplementaryData5.255gradcam_heatmaps/IMG_01.201904250027.01.0018.1556158198.jpg_malignant_gcam_densenet169_finetune.png_raw_image.png]

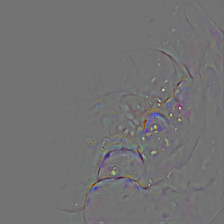

Supplement: Supplementary file 6 — Supplementary Data 5 [file 41467_2020_16777_MOESM6_ESM.gz › SupplementaryData5.255gradcam_heatmaps/IMG_01.201905130039.01.0091.1557714850.jpg_malignant_ggcam_densenet169_finetune.png]

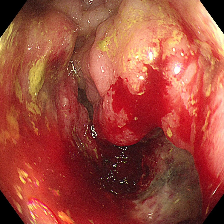

Supplement: Supplementary file 6 — Supplementary Data 5 [file 41467_2020_16777_MOESM6_ESM.gz › SupplementaryData5.255gradcam_heatmaps/IMG_01.201904170017.01.0039.1555465007.jpg_malignant_gcam_densenet169_finetune.png_raw_image.png]

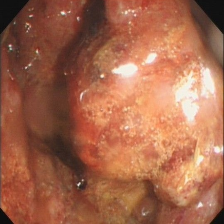

Supplement: Supplementary file 6 — Supplementary Data 5 [file 41467_2020_16777_MOESM6_ESM.gz › SupplementaryData5.255gradcam_heatmaps/IMG_01.201905160036.02.0012.1558315015.jpg_malignant_gcam_densenet169_finetune.png_raw_image.png]

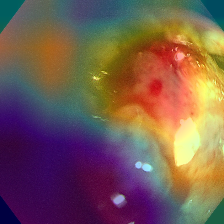

Supplement: Supplementary file 6 — Supplementary Data 5 [file 41467_2020_16777_MOESM6_ESM.gz › SupplementaryData5.255gradcam_heatmaps/IMG_01.201904220001.01.0015.1555890607.jpg_malignant_gcam_densenet169_finetune.png]

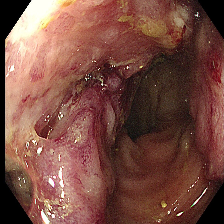

Supplement: Supplementary file 6 — Supplementary Data 5 [file 41467_2020_16777_MOESM6_ESM.gz › SupplementaryData5.255gradcam_heatmaps/IMG_01.201904090018.01.0083.1554772865.jpg_malignant_gcam_densenet169_finetune.png_raw_image.png]

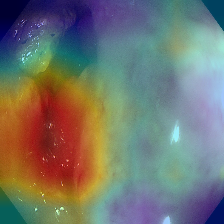

Supplement: Supplementary file 6 — Supplementary Data 5 [file 41467_2020_16777_MOESM6_ESM.gz › SupplementaryData5.255gradcam_heatmaps/IMG_01.201905200051.01.0007.1558330897.jpg_malignant_gcam_densenet169_finetune.png]

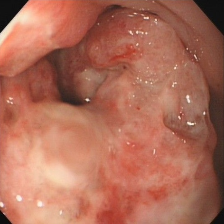

Supplement: Supplementary file 6 — Supplementary Data 5 [file 41467_2020_16777_MOESM6_ESM.gz › SupplementaryData5.255gradcam_heatmaps/IMG_01.201905150002.01.0012.1557878732.jpg_malignant_gcam_densenet169_finetune.png_raw_image.png]

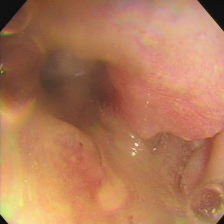

Supplement: Supplementary file 6 — Supplementary Data 5 [file 41467_2020_16777_MOESM6_ESM.gz › SupplementaryData5.255gradcam_heatmaps/IMG_01.201904150008.01.0004.1555287676.jpg_malignant_gcam_densenet169_finetune.png_raw_image.png]

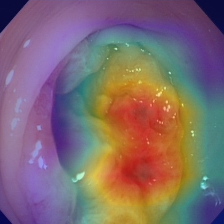

Supplement: Supplementary file 6 — Supplementary Data 5 [file 41467_2020_16777_MOESM6_ESM.gz › SupplementaryData5.255gradcam_heatmaps/IMG_01.201905150002.01.0017.1557878757.jpg_malignant_gcam_densenet169_finetune.png]

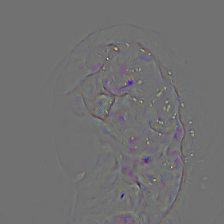

Supplement: Supplementary file 6 — Supplementary Data 5 [file 41467_2020_16777_MOESM6_ESM.gz › SupplementaryData5.255gradcam_heatmaps/IMG_01.201905150002.01.0017.1557878757.jpg_malignant_ggcam_densenet169_finetune.png]

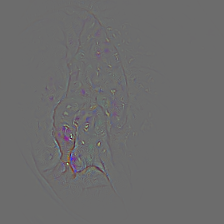

Supplement: Supplementary file 6 — Supplementary Data 5 [file 41467_2020_16777_MOESM6_ESM.gz › SupplementaryData5.255gradcam_heatmaps/IMG_01.201905090069.01.0061.1557383119.jpg_malignant_ggcam_densenet169_finetune.png]

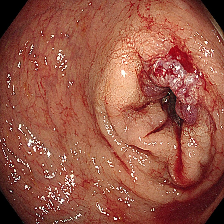

Supplement: Supplementary file 6 — Supplementary Data 5 [file 41467_2020_16777_MOESM6_ESM.gz › SupplementaryData5.255gradcam_heatmaps/IMG_01.201904120017.01.0117.1555034818.jpg_malignant_gcam_densenet169_finetune.png_raw_image.png]

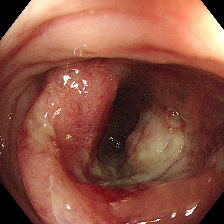

Supplement: Supplementary file 6 — Supplementary Data 5 [file 41467_2020_16777_MOESM6_ESM.gz › SupplementaryData5.255gradcam_heatmaps/IMG_01.201905090079.01.0133.1557387231.jpg_malignant_gcam_densenet169_finetune.png_raw_image.png]

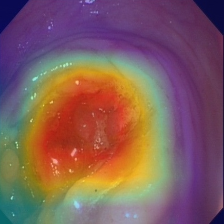

Supplement: Supplementary file 6 — Supplementary Data 5 [file 41467_2020_16777_MOESM6_ESM.gz › SupplementaryData5.255gradcam_heatmaps/IMG_01.201905210026.01.0021.1558402124.jpg_malignant_gcam_densenet169_finetune.png]

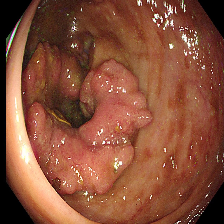

Supplement: Supplementary file 6 — Supplementary Data 5 [file 41467_2020_16777_MOESM6_ESM.gz › SupplementaryData5.255gradcam_heatmaps/IMG_01.201905080051.01.0001.1557284288.jpg_malignant_gcam_densenet169_finetune.png_raw_image.png]

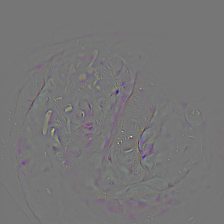

Supplement: Supplementary file 6 — Supplementary Data 5 [file 41467_2020_16777_MOESM6_ESM.gz › SupplementaryData5.255gradcam_heatmaps/IMG_01.201905090079.01.0132.1557387230.jpg_malignant_ggcam_densenet169_finetune.png]

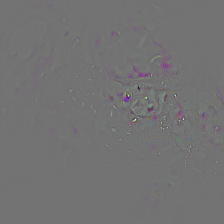

Supplement: Supplementary file 6 — Supplementary Data 5 [file 41467_2020_16777_MOESM6_ESM.gz › SupplementaryData5.255gradcam_heatmaps/IMG_01.201904220033.01.0005.1555898400.jpg_malignant_ggcam_densenet169_finetune.png]

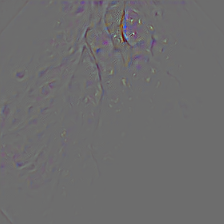

Supplement: Supplementary file 6 — Supplementary Data 5 [file 41467_2020_16777_MOESM6_ESM.gz › SupplementaryData5.255gradcam_heatmaps/IMG_01.201905140036.01.0003.1557800441.jpg_malignant_ggcam_densenet169_finetune.png]

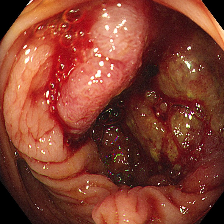

Supplement: Supplementary file 6 — Supplementary Data 5 [file 41467_2020_16777_MOESM6_ESM.gz › SupplementaryData5.255gradcam_heatmaps/IMG_01.201905200019.01.0021.1558315588.jpg_malignant_gcam_densenet169_finetune.png_raw_image.png]

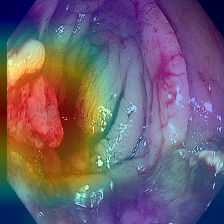

Supplement: Supplementary file 6 — Supplementary Data 5 [file 41467_2020_16777_MOESM6_ESM.gz › SupplementaryData5.255gradcam_heatmaps/IMG_01.201904290044.01.0087.1556508109.jpg_malignant_gcam_densenet169_finetune.png]

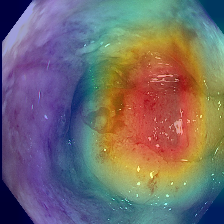

Supplement: Supplementary file 6 — Supplementary Data 5 [file 41467_2020_16777_MOESM6_ESM.gz › SupplementaryData5.255gradcam_heatmaps/IMG_01.201905150021.01.0007.1557885139.jpg_malignant_gcam_densenet169_finetune.png]

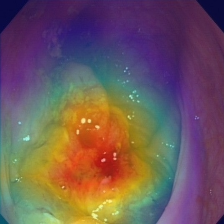

Supplement: Supplementary file 6 — Supplementary Data 5 [file 41467_2020_16777_MOESM6_ESM.gz › SupplementaryData5.255gradcam_heatmaps/IMG_01.201904030029.01.0024.1554255594.jpg_malignant_gcam_densenet169_finetune.png]

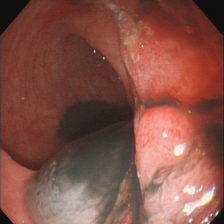

Supplement: Supplementary file 6 — Supplementary Data 5 [file 41467_2020_16777_MOESM6_ESM.gz › SupplementaryData5.255gradcam_heatmaps/IMG_01.201904220002.01.0062.1555890169.jpg_malignant_gcam_densenet169_finetune.png_raw_image.png]

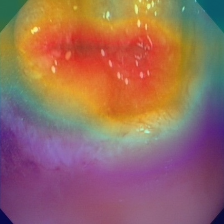

Supplement: Supplementary file 6 — Supplementary Data 5 [file 41467_2020_16777_MOESM6_ESM.gz › SupplementaryData5.255gradcam_heatmaps/IMG_01.201905200027.01.0003.1558315651.jpg_malignant_gcam_densenet169_finetune.png]

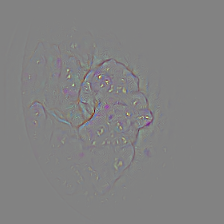

Supplement: Supplementary file 6 — Supplementary Data 5 [file 41467_2020_16777_MOESM6_ESM.gz › SupplementaryData5.255gradcam_heatmaps/IMG_01.201905080051.01.0001.1557284288.jpg_malignant_ggcam_densenet169_finetune.png]

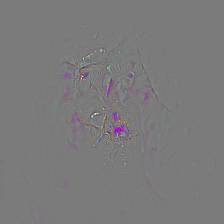

Supplement: Supplementary file 6 — Supplementary Data 5 [file 41467_2020_16777_MOESM6_ESM.gz › SupplementaryData5.255gradcam_heatmaps/IMG_01.201904170017.01.0043.1555465040.jpg_malignant_ggcam_densenet169_finetune.png]

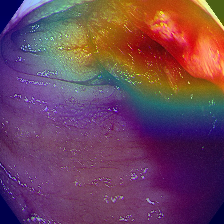

Supplement: Supplementary file 6 — Supplementary Data 5 [file 41467_2020_16777_MOESM6_ESM.gz › SupplementaryData5.255gradcam_heatmaps/IMG_01.201904190024.01.0010.1555638199.jpg_malignant_gcam_densenet169_finetune.png]

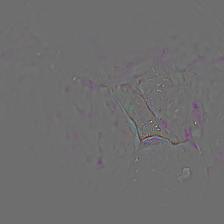

Supplement: Supplementary file 6 — Supplementary Data 5 [file 41467_2020_16777_MOESM6_ESM.gz › SupplementaryData5.255gradcam_heatmaps/IMG_01.201904250027.01.0014.1556158143.jpg_malignant_ggcam_densenet169_finetune.png]

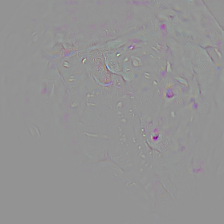

Supplement: Supplementary file 6 — Supplementary Data 5 [file 41467_2020_16777_MOESM6_ESM.gz › SupplementaryData5.255gradcam_heatmaps/IMG_01.201904250027.01.0010.1556158134.jpg_malignant_ggcam_densenet169_finetune.png]

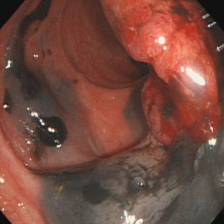

Supplement: Supplementary file 6 — Supplementary Data 5 [file 41467_2020_16777_MOESM6_ESM.gz › SupplementaryData5.255gradcam_heatmaps/IMG_01.201904220002.01.0067.1555890223.jpg_malignant_gcam_densenet169_finetune.png_raw_image.png]

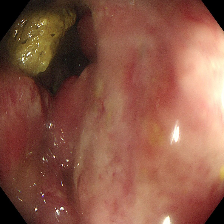

Supplement: Supplementary file 6 — Supplementary Data 5 [file 41467_2020_16777_MOESM6_ESM.gz › SupplementaryData5.255gradcam_heatmaps/IMG_01.201905200051.01.0007.1558330897.jpg_malignant_gcam_densenet169_finetune.png_raw_image.png]

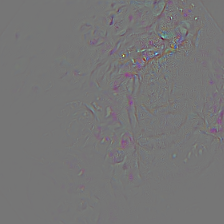

Supplement: Supplementary file 6 — Supplementary Data 5 [file 41467_2020_16777_MOESM6_ESM.gz › SupplementaryData5.255gradcam_heatmaps/IMG_01.201904090018.01.0084.1554772867.jpg_malignant_ggcam_densenet169_finetune.png]

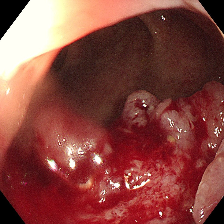

Supplement: Supplementary file 6 — Supplementary Data 5 [file 41467_2020_16777_MOESM6_ESM.gz › SupplementaryData5.255gradcam_heatmaps/IMG_01.201904260014.01.0006.1556241124.jpg_malignant_gcam_densenet169_finetune.png_raw_image.png]

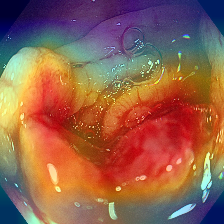

Supplement: Supplementary file 6 — Supplementary Data 5 [file 41467_2020_16777_MOESM6_ESM.gz › SupplementaryData5.255gradcam_heatmaps/IMG_01.201904220036.01.0039.1555899772.jpg_malignant_gcam_densenet169_finetune.png]

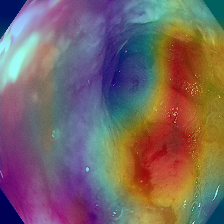

Supplement: Supplementary file 6 — Supplementary Data 5 [file 41467_2020_16777_MOESM6_ESM.gz › SupplementaryData5.255gradcam_heatmaps/IMG_01.201905150021.01.0005.1557885117.jpg_malignant_gcam_densenet169_finetune.png]

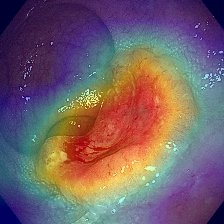

Supplement: Supplementary file 6 — Supplementary Data 5 [file 41467_2020_16777_MOESM6_ESM.gz › SupplementaryData5.255gradcam_heatmaps/IMG_01.201903220015.02.0014.1554078617.jpg_malignant_gcam_densenet169_finetune.png]

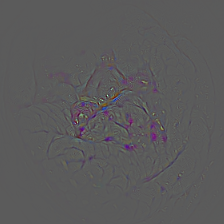

Supplement: Supplementary file 6 — Supplementary Data 5 [file 41467_2020_16777_MOESM6_ESM.gz › SupplementaryData5.255gradcam_heatmaps/IMG_01.201904120017.01.0101.1555034483.jpg_malignant_ggcam_densenet169_finetune.png]

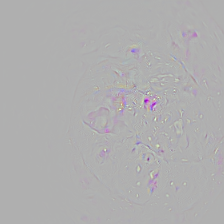

Supplement: Supplementary file 6 — Supplementary Data 5 [file 41467_2020_16777_MOESM6_ESM.gz › SupplementaryData5.255gradcam_heatmaps/IMG_01.201905150021.01.0007.1557885139.jpg_malignant_ggcam_densenet169_finetune.png]
