# Supplementary material for: Diagnostic evaluation of a deep learning model for optical diagnosis of colorectal cancer
Source: Nat Commun. 2020 Jun 11;11:2961. doi: 10.1038/s41467-020-16777-6 (PMC7289893; doi:10.1038/s41467-020-16777-6)
Supplement: Supplementary file 8 — Description of Additional Supplementary Files [file 41467_2020_16777_MOESM8_ESM.pdf]

Title: Supplementary Data 1.

Description: Grad-CAM heatmaps of 36 false negatives.

Title: Supplementary Data 2.

The Grad-CAM heatmaps of representative colonoscopic images paired with haematoxylin-eosin staining images. Tumor location, TNM stage and scale bar were added.

Title: Supplementary Data 3.

Description: The Grad-CAM heatmaps of representative colonoscopic images for flat polyps.

Title: Supplementary Data 4.

Description: The Grad-CAM heatmaps of representative colonoscopic images for sessile serrated polyps.

Title: Supplementary Data 5.

Description: Grad-CAM heatmaps of 255 images.

Title: Supplementary Data 6.

Description: The architecture of convolutional neural network used in this study.
