# Supplementary material for: Diagnostic evaluation of a deep learning model for optical diagnosis of colorectal cancer
Source: Nat Commun. 2020 Jun 11;11:2961. doi: 10.1038/s41467-020-16777-6 (PMC7289893; doi:10.1038/s41467-020-16777-6)
Supplement: Supplementary file 9 — Reporting Summary [file 41467_2020_16777_MOESM9_ESM.pdf]

nature research

Corresponding author(s): Xiangchun Li

Last updated by author(s): May 13, 2020

Reporting Summary

Nature Research wishes to improve the reproducibility of the work that we publish. This form provides structure for consistency and transparency in reporting. For further information on Nature Research policies, see Authors & Referees and the Editorial Policy Checklist .

Statistics

For all statistical analyses, confirm that the following items are present in the figure legend, table legend, main text, or Methods section.

n/a	Confirmed	
☐	☒	The exact sample size (*n*) for each experimental group/condition, given as a discrete number and unit of measurement
☐	☒	A statement on whether measurements were taken from distinct samples or whether the same sample was measured repeatedly
☐	☒	The statistical test(s) used AND whether they are one- or two-sided<br>*Only common tests should be described solely by name; describe more complex techniques in the Methods section.*
☒	☐	A description of all covariates tested
☒	☐	A description of any assumptions or corrections, such as tests of normality and adjustment for multiple comparisons
☐	☒	A full description of the statistical parameters including central tendency (e.g. means) or other basic estimates (e.g. regression coefficient) AND variation (e.g. standard deviation) or associated estimates of uncertainty (e.g. confidence intervals)
☐	☒	For null hypothesis testing, the test statistic (e.g. *F*, *t*, *r*) with confidence intervals, effect sizes, degrees of freedom and *P* value noted<br>*Give P values as exact values whenever suitable.*
☒	☐	For Bayesian analysis, information on the choice of priors and Markov chain Monte Carlo settings
☒	☐	For hierarchical and complex designs, identification of the appropriate level for tests and full reporting of outcomes
☒	☐	Estimates of effect sizes (e.g. Cohen's *d*, Pearson's *r*), indicating how they were calculated

Our web collection on statistics for biologists contains articles on many of the points above.

Software and code

Policy information about availability of computer code

Data collection	The code used to train and evaluate the model is available on github (https://github.com/lixiangchun/AIplus/tree/master/CRCNet).
Data analysis	We developed a deep learning model called CRCNet by finetuning a densely connected convolutional network of 169 layers with colonoscopic images to differentiate colorectal cancers from non-cancerous controls. All image labels were derived from pathological examination reports. For each individual, we calculated a malignancy score by using weighted mean of log10 transformation of all images from this individual. The classification performance of our model was validated in one internal and two external data sets, and compared with skilled endoscopists. The deep learning pipeline was developed with Python (v3.7.1), PyTorch (v1.3.0) and torchvision (v0.5.0).

For manuscripts utilizing custom algorithms or software that are central to the research but not yet described in published literature, software must be made available to editors/reviewers. We strongly encourage code deposition in a community repository (e.g. GitHub). See the Nature Research guidelines for submitting code & software for further information.

Data

Policy information about availability of data

All manuscripts must include a data availability statement. This statement should provide the following information, where applicable:

- Accession codes, unique identifiers, or web links for publicly available datasets
- A list of figures that have associated raw data
- A description of any restrictions on data availability

The authors declare that the data supporting the findings of this study are available within the paper and its supplementary information files. Restrictions apply to the availability of the training and test sets, which were used with permission for the current study, and so are not publicly available. Databases used include Colonoscopic Imaging Databases of Tianjin Medical University Cancer Institute and Hospital and ImageNet (http://www.image-net.org/).

Field-specific reporting

Please select the one below that is the best fit for your research. If you are not sure, read the appropriate sections before making your selection.

☒ Life sciences ☐ Behavioural & social sciences ☐ Ecological, evolutionary & environmental sciences

For a reference copy of the document with all sections, see nature.com/documents/nr-reporting-summary-flat.pdf

Life sciences study design

All studies must disclose on these points even when the disclosure is negative.

Sample size	No sample-size calculation was performed. We used all the images retrieved from Colonoscopic Imaging Database. The sample size of the training set is 464,105, which is sufficient to finetune a deep learning model. The number of images in three test sets are 20,783, 15,441 and 48,391, respectively.
Data exclusions	We excluded 13,522 low quality images such as motion-blurring, blank, out-of-focus or poor bowel preparation.
Replication	The findings were successfully validated in three independent test sets.
Randomization	This is not relevant to our study as it is not clinical trial.
Blinding	Group allocation of training set is not blind to investigators, while it was blind to investigators for the test set during data collection. In preparation of training set, endoscopists were asked to read pathological reports and imaging data of colorectal cancer patients to group colonoscopic images into malignancy or benignity; thus it is not blind to investigators in group allocation of training set. During data analysis, investigators were blinded to group allocation.

Reporting for specific materials, systems and methods

We require information from authors about some types of materials, experimental systems and methods used in many studies. Here, indicate whether each material, system or method listed is relevant to your study. If you are not sure if a list item applies to your research, read the appropriate section before selecting a response.

Materials & experimental systems

n/a	Involved in the study	
☒	☐	Antibodies
☒	☐	Eukaryotic cell lines
☒	☐	Palaeontology
☒	☐	Animals and other organisms
☐	☒	Human research participants
☒	☐	Clinical data

Methods

n/a	Involved in the study	
☒	☐	ChIP-seq
☒	☐	Flow cytometry
☒	☐	MRI-based neuroimaging

Human research participants

Policy information about studies involving human research participants

Population characteristics	Male and female patients were included. Ages are 60 (53 - 67) for colorectal cancer patients and 57 (49 - 64) for non-cancerous controls in the training set. For colorectal cancer patients and non-cancerous controls in the three test sets, ages are 61 (53 - 66) and 59 (52 - 66), 63 (53.3 - 72) and 58.5 (50 - 65), and 66 (59 - 74) and 58 (47.5 - 65), respectively.
Recruitment	This is a retrospective study so all participants extracted from the imaging database were included.
Ethics oversight	This study was approved by the Institutional Review Board of Tianjin Medical University Cancer Institute and Hospital.

Note that full information on the approval of the study protocol must also be provided in the manuscript.
