# Supplementary material for: Diagnostic evaluation of a deep learning model for optical diagnosis of colorectal cancer
Source: Nat Commun. 2020 Jun 11;11:2961. doi: 10.1038/s41467-020-16777-6 (PMC7289893; doi:10.1038/s41467-020-16777-6)
Supplement: Supplementary file 7 — Supplementary Data 6 [file 41467_2020_16777_MOESM7_ESM.pdf]

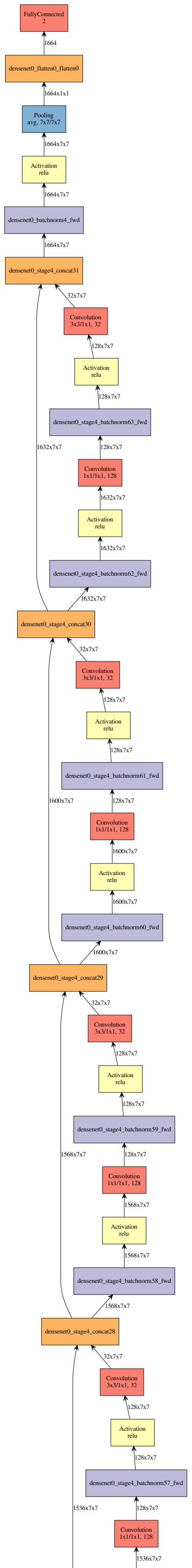
FullyConnected
2
1664
densenet0_flatten0_flatten0
1664x1x1
Pooling
avg, 7x7/7x7
1664x7x7
Activation
relu
1664x7x7
densenet0_batchnorm4_fwd
1664x7x7
densenet0_stage4_concat31
32x7x7
Convolution
3x3/1x1, 32
128x7x7
Activation
relu
128x7x7
densenet0_stage4_batchnorm63_fwd
128x7x7
1632x7x7
Convolution
1x1/1x1, 128
1632x7x7
Activation
relu
1632x7x7
densenet0_stage4_batchnorm62_fwd
1632x7x7
densenet0_stage4_concat30
32x7x7
Convolution
3x3/1x1, 32
128x7x7
Activation
relu
128x7x7
densenet0_stage4_batchnorm61_fwd
128x7x7
1600x7x7
Convolution
1x1/1x1, 128
1600x7x7
Activation
relu
1600x7x7
densenet0_stage4_batchnorm60_fwd
1600x7x7
densenet0_stage4_concat29
32x7x7
Convolution
3x3/1x1, 32
128x7x7
Activation
relu
128x7x7
densenet0_stage4_batchnorm59_fwd
128x7x7
1568x7x7
Convolution
1x1/1x1, 128
1568x7x7
Activation
relu
1568x7x7
densenet0_stage4_batchnorm58_fwd
1568x7x7
densenet0_stage4_concat28
32x7x7
Convolution
3x3/1x1, 32
128x7x7
Activation
relu
128x7x7
densenet0_stage4_batchnorm57_fwd
128x7x7
1536x7x7
Convolution
1x1/1x1, 128
1536x7x7

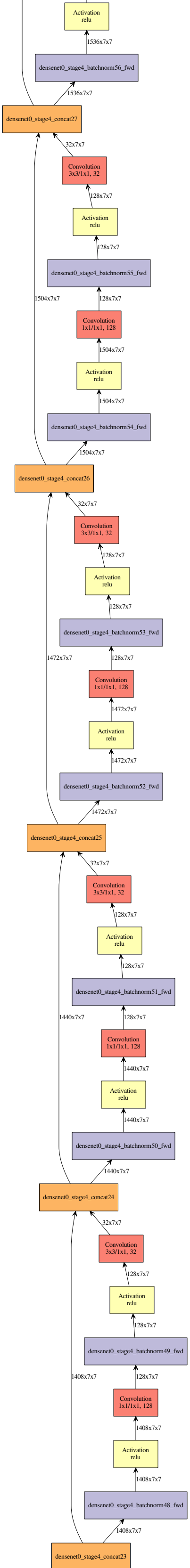
Activation
relu
1536x7x7
densenet0_stage4_batchnorm56_fwd
1536x7x7
densenet0_stage4_concat27
32x7x7
Convolution
3x3/1x1, 32
128x7x7
Activation
relu
128x7x7
densenet0_stage4_batchnorm55_fwd
128x7x7
1504x7x7
Convolution
1x1/1x1, 128
1504x7x7
Activation
relu
1504x7x7
densenet0_stage4_batchnorm54_fwd
1504x7x7
densenet0_stage4_concat26
32x7x7
Convolution
3x3/1x1, 32
128x7x7
Activation
relu
128x7x7
densenet0_stage4_batchnorm53_fwd
128x7x7
1472x7x7
Convolution
1x1/1x1, 128
1472x7x7
Activation
relu
1472x7x7
densenet0_stage4_batchnorm52_fwd
1472x7x7
densenet0_stage4_concat25
32x7x7
Convolution
3x3/1x1, 32
128x7x7
Activation
relu
128x7x7
densenet0_stage4_batchnorm51_fwd
128x7x7
1440x7x7
Convolution
1x1/1x1, 128
1440x7x7
Activation
relu
1440x7x7
densenet0_stage4_batchnorm50_fwd
1440x7x7
densenet0_stage4_concat24
32x7x7
Convolution
3x3/1x1, 32
128x7x7
Activation
relu
128x7x7
densenet0_stage4_batchnorm49_fwd
128x7x7
1408x7x7
Convolution
1x1/1x1, 128
1408x7x7
Activation
relu
1408x7x7
densenet0_stage4_batchnorm48_fwd
1408x7x7
densenet0_stage4_concat23

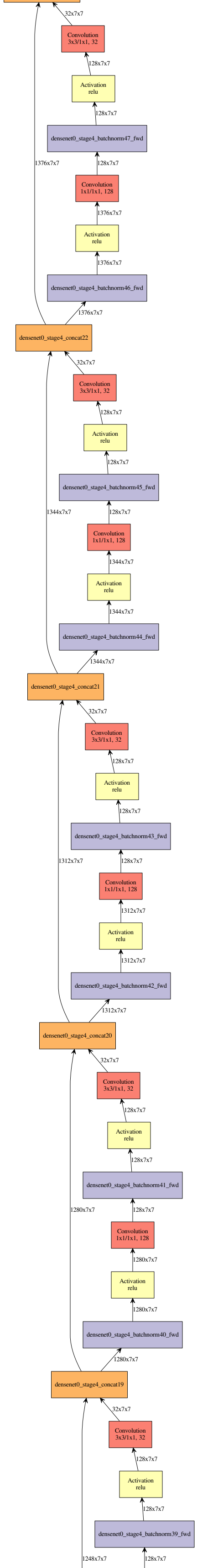
32x7x7
Convolution
3x3/1x1, 32
128x7x7
Activation
relu
128x7x7
densenet0_stage4_batchnorm47_fwd
1376x7x7
128x7x7
Convolution
1x1/1x1, 128
1376x7x7
Activation
relu
1376x7x7
densenet0_stage4_batchnorm46_fwd
1376x7x7
densenet0_stage4_concat22
32x7x7
Convolution
3x3/1x1, 32
128x7x7
Activation
relu
128x7x7
densenet0_stage4_batchnorm45_fwd
1344x7x7
128x7x7
Convolution
1x1/1x1, 128
1344x7x7
Activation
relu
1344x7x7
densenet0_stage4_batchnorm44_fwd
1344x7x7
densenet0_stage4_concat21
32x7x7
Convolution
3x3/1x1, 32
128x7x7
Activation
relu
128x7x7
densenet0_stage4_batchnorm43_fwd
1312x7x7
128x7x7
Convolution
1x1/1x1, 128
1312x7x7
Activation
relu
1312x7x7
densenet0_stage4_batchnorm42_fwd
1312x7x7
densenet0_stage4_concat20
32x7x7
Convolution
3x3/1x1, 32
128x7x7
Activation
relu
128x7x7
densenet0_stage4_batchnorm41_fwd
1280x7x7
128x7x7
Convolution
1x1/1x1, 128
1280x7x7
Activation
relu
1280x7x7
densenet0_stage4_batchnorm40_fwd
1280x7x7
densenet0_stage4_concat19
32x7x7
Convolution
3x3/1x1, 32
128x7x7
Activation
relu
128x7x7
densenet0_stage4_batchnorm39_fwd
1248x7x7
128x7x7

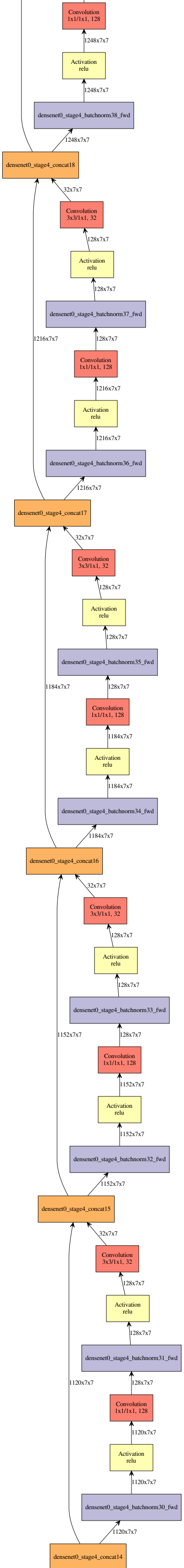
Convolution
1x1/1x1, 128
1248x7x7
Activation
relu
1248x7x7
densenet0_stage4_batchnorm38_fwd
1248x7x7
densenet0_stage4_concat18
32x7x7
Convolution
3x3/1x1, 32
128x7x7
Activation
relu
128x7x7
densenet0_stage4_batchnorm37_fwd
1216x7x7
128x7x7
Convolution
1x1/1x1, 128
1216x7x7
Activation
relu
1216x7x7
densenet0_stage4_batchnorm36_fwd
1216x7x7
densenet0_stage4_concat17
32x7x7
Convolution
3x3/1x1, 32
128x7x7
Activation
relu
128x7x7
densenet0_stage4_batchnorm35_fwd
1184x7x7
128x7x7
Convolution
1x1/1x1, 128
1184x7x7
Activation
relu
1184x7x7
densenet0_stage4_batchnorm34_fwd
1184x7x7
densenet0_stage4_concat16
32x7x7
Convolution
3x3/1x1, 32
128x7x7
Activation
relu
128x7x7
densenet0_stage4_batchnorm33_fwd
1152x7x7
128x7x7
Convolution
1x1/1x1, 128
1152x7x7
Activation
relu
1152x7x7
densenet0_stage4_batchnorm32_fwd
1152x7x7
densenet0_stage4_concat15
32x7x7
Convolution
3x3/1x1, 32
128x7x7
Activation
relu
128x7x7
densenet0_stage4_batchnorm31_fwd
1120x7x7
128x7x7
Convolution
1x1/1x1, 128
1120x7x7
Activation
relu
1120x7x7
densenet0_stage4_batchnorm30_fwd
1120x7x7
densenet0_stage4_concat14

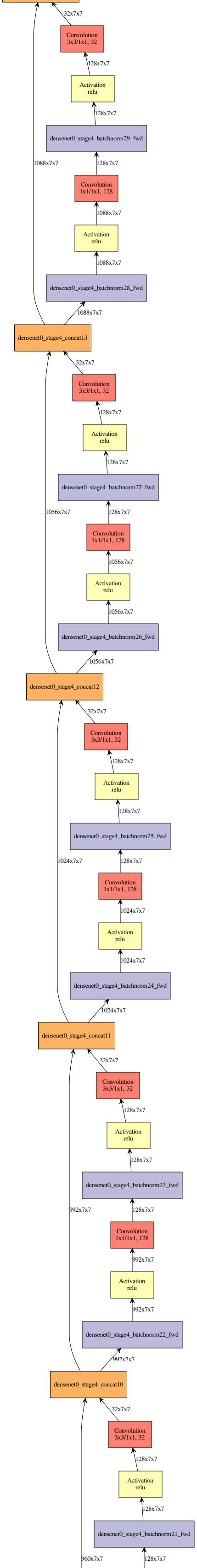
32x7x7
Convolution
3x3/1x1, 32
128x7x7
Activation
relu
128x7x7
densenet0_stage4_batchnorm29_fwd
1088x7x7
128x7x7
Convolution
1x1/1x1, 128
1088x7x7
Activation
relu
1088x7x7
densenet0_stage4_batchnorm28_fwd
1088x7x7
densenet0_stage4_concat13
32x7x7
Convolution
3x3/1x1, 32
128x7x7
Activation
relu
128x7x7
densenet0_stage4_batchnorm27_fwd
1056x7x7
128x7x7
Convolution
1x1/1x1, 128
1056x7x7
Activation
relu
1056x7x7
densenet0_stage4_batchnorm26_fwd
1056x7x7
densenet0_stage4_concat12
32x7x7
Convolution
3x3/1x1, 32
128x7x7
Activation
relu
128x7x7
densenet0_stage4_batchnorm25_fwd
1024x7x7
128x7x7
Convolution
1x1/1x1, 128
1024x7x7
Activation
relu
1024x7x7
densenet0_stage4_batchnorm24_fwd
1024x7x7
densenet0_stage4_concat11
32x7x7
Convolution
3x3/1x1, 32
128x7x7
Activation
relu
128x7x7
densenet0_stage4_batchnorm23_fwd
992x7x7
128x7x7
Convolution
1x1/1x1, 128
992x7x7
Activation
relu
992x7x7
densenet0_stage4_batchnorm22_fwd
992x7x7
densenet0_stage4_concat10
32x7x7
Convolution
3x3/1x1, 32
128x7x7
Activation
relu
128x7x7
densenet0_stage4_batchnorm21_fwd
960x7x7
128x7x7

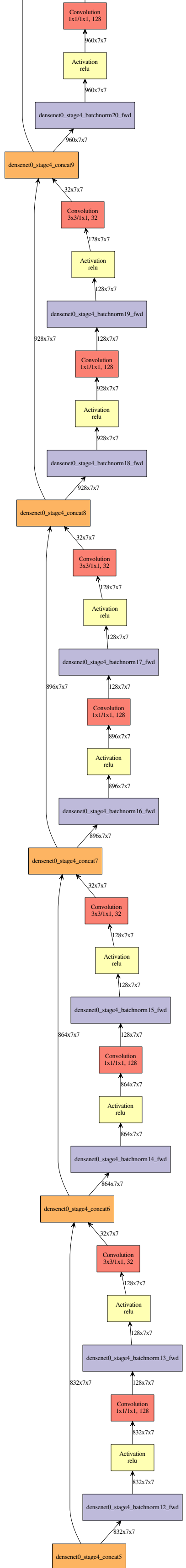
Convolution
1x1/1x1, 128
960x7x7
Activation
relu
960x7x7
densenet0_stage4_batchnorm20_fwd
960x7x7
densenet0_stage4_concat9
32x7x7
Convolution
3x3/1x1, 32
128x7x7
Activation
relu
128x7x7
densenet0_stage4_batchnorm19_fwd
928x7x7
128x7x7
Convolution
1x1/1x1, 128
928x7x7
Activation
relu
928x7x7
densenet0_stage4_batchnorm18_fwd
928x7x7
densenet0_stage4_concat8
32x7x7
Convolution
3x3/1x1, 32
128x7x7
Activation
relu
128x7x7
densenet0_stage4_batchnorm17_fwd
896x7x7
128x7x7
Convolution
1x1/1x1, 128
896x7x7
Activation
relu
896x7x7
densenet0_stage4_batchnorm16_fwd
896x7x7
densenet0_stage4_concat7
32x7x7
Convolution
3x3/1x1, 32
128x7x7
Activation
relu
128x7x7
densenet0_stage4_batchnorm15_fwd
864x7x7
128x7x7
Convolution
1x1/1x1, 128
864x7x7
Activation
relu
864x7x7
densenet0_stage4_batchnorm14_fwd
864x7x7
densenet0_stage4_concat6
32x7x7
Convolution
3x3/1x1, 32
128x7x7
Activation
relu
128x7x7
densenet0_stage4_batchnorm13_fwd
832x7x7
128x7x7
Convolution
1x1/1x1, 128
832x7x7
Activation
relu
832x7x7
densenet0_stage4_batchnorm12_fwd
832x7x7
densenet0_stage4_concat5

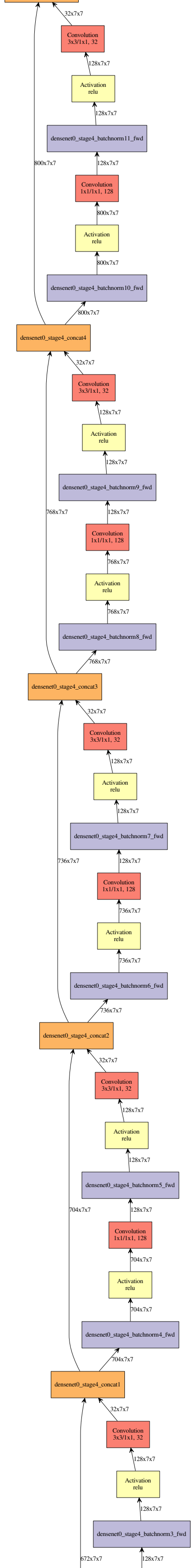

32x7x7
Convolution 3x3/1x1, 32
128x7x7
Activation relu
128x7x7
densenet0_stage4_batchnorm11_fwd
800x7x7
128x7x7
Convolution 1x1/1x1, 128
800x7x7
Activation relu
800x7x7
densenet0_stage4_batchnorm10_fwd
800x7x7
densenet0_stage4_concat4
32x7x7
Convolution 3x3/1x1, 32
128x7x7
Activation relu
128x7x7
densenet0_stage4_batchnorm9_fwd
768x7x7
128x7x7
Convolution 1x1/1x1, 128
768x7x7
Activation relu
768x7x7
densenet0_stage4_batchnorm8_fwd
768x7x7
densenet0_stage4_concat3
32x7x7
Convolution 3x3/1x1, 32
128x7x7
Activation relu
128x7x7
densenet0_stage4_batchnorm7_fwd
736x7x7
128x7x7
Convolution 1x1/1x1, 128
736x7x7
Activation relu
736x7x7
densenet0_stage4_batchnorm6_fwd
736x7x7
densenet0_stage4_concat2
32x7x7
Convolution 3x3/1x1, 32
128x7x7
Activation relu
128x7x7
densenet0_stage4_batchnorm5_fwd
704x7x7
128x7x7
Convolution 1x1/1x1, 128
704x7x7
Activation relu
704x7x7
densenet0_stage4_batchnorm4_fwd
704x7x7
densenet0_stage4_concat1
32x7x7
Convolution 3x3/1x1, 32
128x7x7
Activation relu
128x7x7
densenet0_stage4_batchnorm3_fwd
672x7x7
128x7x7

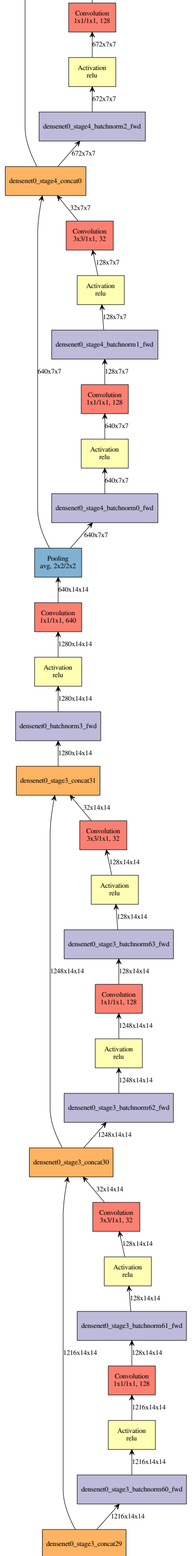

Convolution
1x1/1x1, 128
672x7x7
Activation
relu
672x7x7
densenet0_stage4_batchnorm2_fwd
672x7x7
densenet0_stage4_concat0
32x7x7
Convolution
3x3/1x1, 32
128x7x7
Activation
relu
128x7x7
densenet0_stage4_batchnorm1_fwd
128x7x7
640x7x7
Convolution
1x1/1x1, 128
640x7x7
Activation
relu
640x7x7
densenet0_stage4_batchnorm0_fwd
640x7x7
Pooling
avg, 2x2/2x2
640x14x14
Convolution
1x1/1x1, 640
1280x14x14
Activation
relu
1280x14x14
densenet0_batchnorm3_fwd
1280x14x14
densenet0_stage3_concat31
32x14x14
Convolution
3x3/1x1, 32
128x14x14
Activation
relu
128x14x14
densenet0_stage3_batchnorm63_fwd
128x14x14
1248x14x14
Convolution
1x1/1x1, 128
1248x14x14
Activation
relu
1248x14x14
densenet0_stage3_batchnorm62_fwd
1248x14x14
densenet0_stage3_concat30
32x14x14
Convolution
3x3/1x1, 32
128x14x14
Activation
relu
128x14x14
densenet0_stage3_batchnorm61_fwd
128x14x14
1216x14x14
Convolution
1x1/1x1, 128
1216x14x14
Activation
relu
1216x14x14
densenet0_stage3_batchnorm60_fwd
1216x14x14
densenet0_stage3_concat29

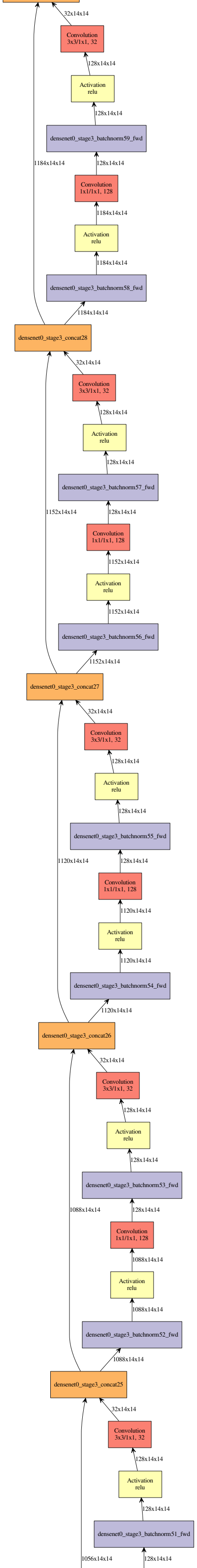

32x14x14
Convolution
3x3/1x1, 32
128x14x14
Activation
relu
128x14x14
densenet0_stage3_batchnorm59_fwd
1184x14x14
128x14x14
Convolution
1x1/1x1, 128
1184x14x14
Activation
relu
1184x14x14
densenet0_stage3_batchnorm58_fwd
1184x14x14
densenet0_stage3_concat28
32x14x14
Convolution
3x3/1x1, 32
128x14x14
Activation
relu
128x14x14
densenet0_stage3_batchnorm57_fwd
1152x14x14
128x14x14
Convolution
1x1/1x1, 128
1152x14x14
Activation
relu
1152x14x14
densenet0_stage3_batchnorm56_fwd
1152x14x14
densenet0_stage3_concat27
32x14x14
Convolution
3x3/1x1, 32
128x14x14
Activation
relu
128x14x14
densenet0_stage3_batchnorm55_fwd
1120x14x14
128x14x14
Convolution
1x1/1x1, 128
1120x14x14
Activation
relu
1120x14x14
densenet0_stage3_batchnorm54_fwd
1120x14x14
densenet0_stage3_concat26
32x14x14
Convolution
3x3/1x1, 32
128x14x14
Activation
relu
128x14x14
densenet0_stage3_batchnorm53_fwd
1088x14x14
128x14x14
Convolution
1x1/1x1, 128
1088x14x14
Activation
relu
1088x14x14
densenet0_stage3_batchnorm52_fwd
1088x14x14
densenet0_stage3_concat25
32x14x14
Convolution
3x3/1x1, 32
128x14x14
Activation
relu
128x14x14
densenet0_stage3_batchnorm51_fwd
1056x14x14
128x14x14

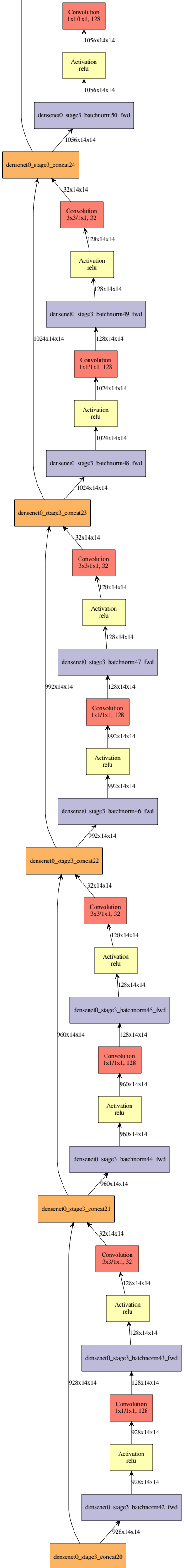
Convolution
1x1/1x1, 128
1056x14x14
Activation
relu
1056x14x14
densenet0_stage3_batchnorm50_fwd
1056x14x14
densenet0_stage3_concat24
32x14x14
Convolution
3x3/1x1, 32
128x14x14
Activation
relu
128x14x14
densenet0_stage3_batchnorm49_fwd
128x14x14
1024x14x14
Convolution
1x1/1x1, 128
1024x14x14
Activation
relu
1024x14x14
densenet0_stage3_batchnorm48_fwd
1024x14x14
densenet0_stage3_concat23
32x14x14
Convolution
3x3/1x1, 32
128x14x14
Activation
relu
128x14x14
densenet0_stage3_batchnorm47_fwd
128x14x14
992x14x14
Convolution
1x1/1x1, 128
992x14x14
Activation
relu
992x14x14
densenet0_stage3_batchnorm46_fwd
992x14x14
densenet0_stage3_concat22
32x14x14
Convolution
3x3/1x1, 32
128x14x14
Activation
relu
128x14x14
densenet0_stage3_batchnorm45_fwd
128x14x14
960x14x14
Convolution
1x1/1x1, 128
960x14x14
Activation
relu
960x14x14
densenet0_stage3_batchnorm44_fwd
960x14x14
densenet0_stage3_concat21
32x14x14
Convolution
3x3/1x1, 32
128x14x14
Activation
relu
128x14x14
densenet0_stage3_batchnorm43_fwd
128x14x14
928x14x14
Convolution
1x1/1x1, 128
928x14x14
Activation
relu
928x14x14
densenet0_stage3_batchnorm42_fwd
928x14x14
densenet0_stage3_concat20

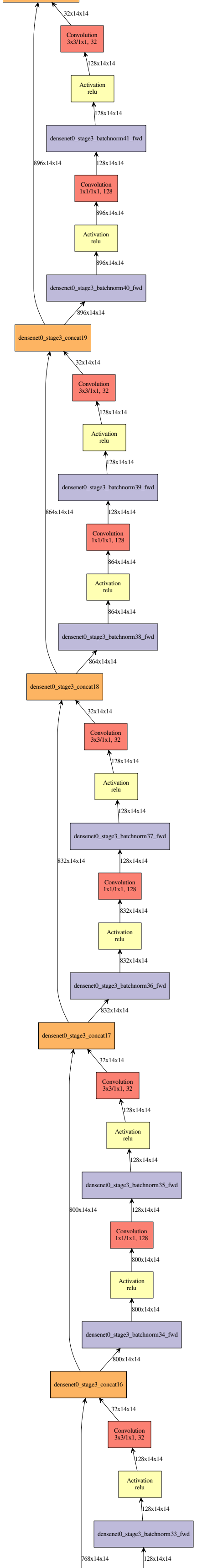

32x14x14
Convolution
3x3/1x1, 32
128x14x14
Activation
relu
128x14x14
densenet0_stage3_batchnorm41_fwd
896x14x14
128x14x14
Convolution
1x1/1x1, 128
896x14x14
Activation
relu
896x14x14
densenet0_stage3_batchnorm40_fwd
896x14x14
densenet0_stage3_concat19
32x14x14
Convolution
3x3/1x1, 32
128x14x14
Activation
relu
128x14x14
densenet0_stage3_batchnorm39_fwd
864x14x14
128x14x14
Convolution
1x1/1x1, 128
864x14x14
Activation
relu
864x14x14
densenet0_stage3_batchnorm38_fwd
864x14x14
densenet0_stage3_concat18
32x14x14
Convolution
3x3/1x1, 32
128x14x14
Activation
relu
128x14x14
densenet0_stage3_batchnorm37_fwd
832x14x14
128x14x14
Convolution
1x1/1x1, 128
832x14x14
Activation
relu
832x14x14
densenet0_stage3_batchnorm36_fwd
832x14x14
densenet0_stage3_concat17
32x14x14
Convolution
3x3/1x1, 32
128x14x14
Activation
relu
128x14x14
densenet0_stage3_batchnorm35_fwd
800x14x14
128x14x14
Convolution
1x1/1x1, 128
800x14x14
Activation
relu
800x14x14
densenet0_stage3_batchnorm34_fwd
800x14x14
densenet0_stage3_concat16
32x14x14
Convolution
3x3/1x1, 32
128x14x14
Activation
relu
128x14x14
densenet0_stage3_batchnorm33_fwd
768x14x14
128x14x14

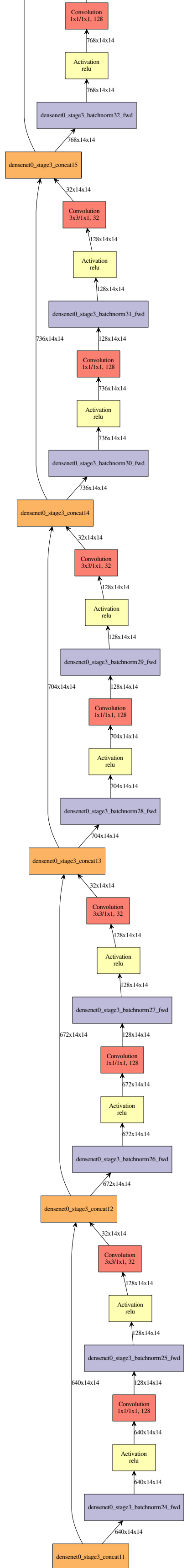
Convolution
1x1/1x1, 128
768x14x14
Activation
relu
768x14x14
densenet0_stage3_batchnorm32_fwd
768x14x14
densenet0_stage3_concat15
32x14x14
Convolution
3x3/1x1, 32
128x14x14
Activation
relu
128x14x14
densenet0_stage3_batchnorm31_fwd
736x14x14
128x14x14
Convolution
1x1/1x1, 128
736x14x14
Activation
relu
736x14x14
densenet0_stage3_batchnorm30_fwd
736x14x14
densenet0_stage3_concat14
32x14x14
Convolution
3x3/1x1, 32
128x14x14
Activation
relu
128x14x14
densenet0_stage3_batchnorm29_fwd
704x14x14
128x14x14
Convolution
1x1/1x1, 128
704x14x14
Activation
relu
704x14x14
densenet0_stage3_batchnorm28_fwd
704x14x14
densenet0_stage3_concat13
32x14x14
Convolution
3x3/1x1, 32
128x14x14
Activation
relu
128x14x14
densenet0_stage3_batchnorm27_fwd
672x14x14
128x14x14
Convolution
1x1/1x1, 128
672x14x14
Activation
relu
672x14x14
densenet0_stage3_batchnorm26_fwd
672x14x14
densenet0_stage3_concat12
32x14x14
Convolution
3x3/1x1, 32
128x14x14
Activation
relu
128x14x14
densenet0_stage3_batchnorm25_fwd
640x14x14
128x14x14
Convolution
1x1/1x1, 128
640x14x14
Activation
relu
640x14x14
densenet0_stage3_batchnorm24_fwd
640x14x14
densenet0_stage3_concat11

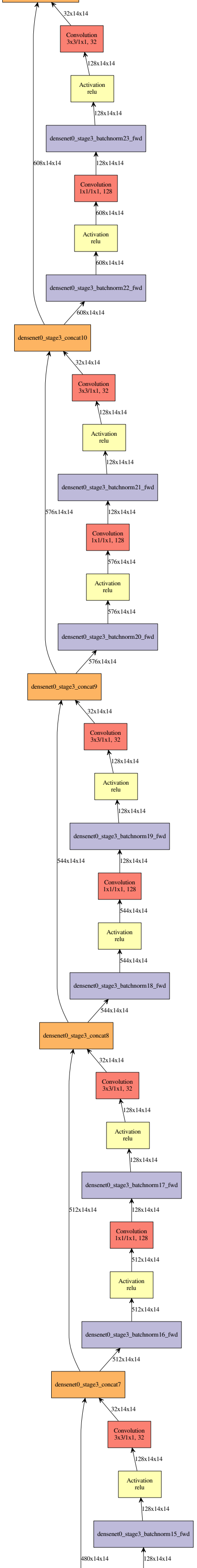
32x14x14
Convolution
3x3/1x1, 32
128x14x14
Activation
relu
128x14x14
densenet0_stage3_batchnorm23_fwd
608x14x14
128x14x14
Convolution
1x1/1x1, 128
608x14x14
Activation
relu
608x14x14
densenet0_stage3_batchnorm22_fwd
608x14x14
densenet0_stage3_concat10
32x14x14
Convolution
3x3/1x1, 32
128x14x14
Activation
relu
128x14x14
densenet0_stage3_batchnorm21_fwd
576x14x14
128x14x14
Convolution
1x1/1x1, 128
576x14x14
Activation
relu
576x14x14
densenet0_stage3_batchnorm20_fwd
576x14x14
densenet0_stage3_concat9
32x14x14
Convolution
3x3/1x1, 32
128x14x14
Activation
relu
128x14x14
densenet0_stage3_batchnorm19_fwd
544x14x14
128x14x14
Convolution
1x1/1x1, 128
544x14x14
Activation
relu
544x14x14
densenet0_stage3_batchnorm18_fwd
544x14x14
densenet0_stage3_concat8
32x14x14
Convolution
3x3/1x1, 32
128x14x14
Activation
relu
128x14x14
densenet0_stage3_batchnorm17_fwd
512x14x14
128x14x14
Convolution
1x1/1x1, 128
512x14x14
Activation
relu
512x14x14
densenet0_stage3_batchnorm16_fwd
512x14x14
densenet0_stage3_concat7
32x14x14
Convolution
3x3/1x1, 32
128x14x14
Activation
relu
128x14x14
densenet0_stage3_batchnorm15_fwd
480x14x14
128x14x14

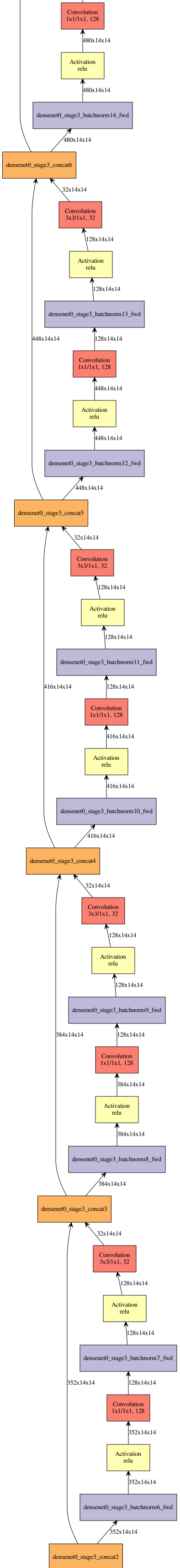

Convolution
1x1/1x1, 128
480x14x14
Activation
relu
480x14x14
densenet0_stage3_batchnorm14_fwd
480x14x14
densenet0_stage3_concat6
32x14x14
Convolution
3x3/1x1, 32
128x14x14
Activation
relu
128x14x14
densenet0_stage3_batchnorm13_fwd
448x14x14
128x14x14
Convolution
1x1/1x1, 128
448x14x14
Activation
relu
448x14x14
densenet0_stage3_batchnorm12_fwd
448x14x14
densenet0_stage3_concat5
32x14x14
Convolution
3x3/1x1, 32
128x14x14
Activation
relu
128x14x14
densenet0_stage3_batchnorm11_fwd
416x14x14
128x14x14
Convolution
1x1/1x1, 128
416x14x14
Activation
relu
416x14x14
densenet0_stage3_batchnorm10_fwd
416x14x14
densenet0_stage3_concat4
32x14x14
Convolution
3x3/1x1, 32
128x14x14
Activation
relu
128x14x14
densenet0_stage3_batchnorm9_fwd
384x14x14
128x14x14
Convolution
1x1/1x1, 128
384x14x14
Activation
relu
384x14x14
densenet0_stage3_batchnorm8_fwd
384x14x14
densenet0_stage3_concat3
32x14x14
Convolution
3x3/1x1, 32
128x14x14
Activation
relu
128x14x14
densenet0_stage3_batchnorm7_fwd
352x14x14
128x14x14
Convolution
1x1/1x1, 128
352x14x14
Activation
relu
352x14x14
densenet0_stage3_batchnorm6_fwd
352x14x14
densenet0_stage3_concat2

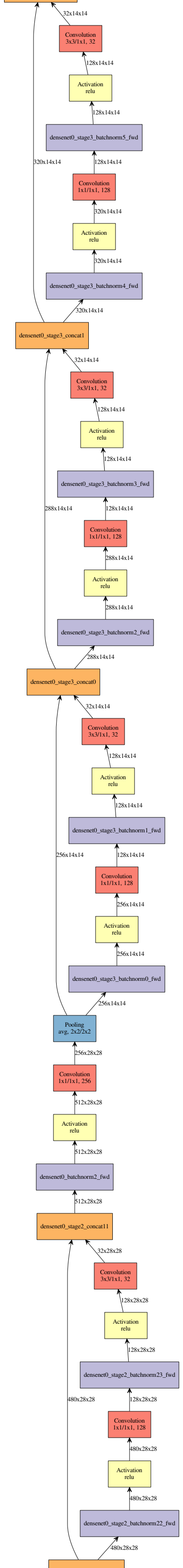
32x14x14
Convolution
3x3/1x1, 32
128x14x14
Activation
relu
128x14x14
densenet0_stage3_batchnorm5_fwd
320x14x14
128x14x14
Convolution
1x1/1x1, 128
320x14x14
Activation
relu
320x14x14
densenet0_stage3_batchnorm4_fwd
320x14x14
densenet0_stage3_concat1
32x14x14
Convolution
3x3/1x1, 32
128x14x14
Activation
relu
128x14x14
densenet0_stage3_batchnorm3_fwd
288x14x14
128x14x14
Convolution
1x1/1x1, 128
288x14x14
Activation
relu
288x14x14
densenet0_stage3_batchnorm2_fwd
288x14x14
densenet0_stage3_concat0
32x14x14
Convolution
3x3/1x1, 32
128x14x14
Activation
relu
128x14x14
densenet0_stage3_batchnorm1_fwd
256x14x14
128x14x14
Convolution
1x1/1x1, 128
256x14x14
Activation
relu
256x14x14
densenet0_stage3_batchnorm0_fwd
256x14x14
Pooling
avg, 2x2/2x2
256x28x28
Convolution
1x1/1x1, 256
512x28x28
Activation
relu
512x28x28
densenet0_batchnorm2_fwd
512x28x28
densenet0_stage2_concat11
32x28x28
Convolution
3x3/1x1, 32
128x28x28
Activation
relu
128x28x28
densenet0_stage2_batchnorm23_fwd
480x28x28
128x28x28
Convolution
1x1/1x1, 128
480x28x28
Activation
relu
480x28x28
densenet0_stage2_batchnorm22_fwd
480x28x28

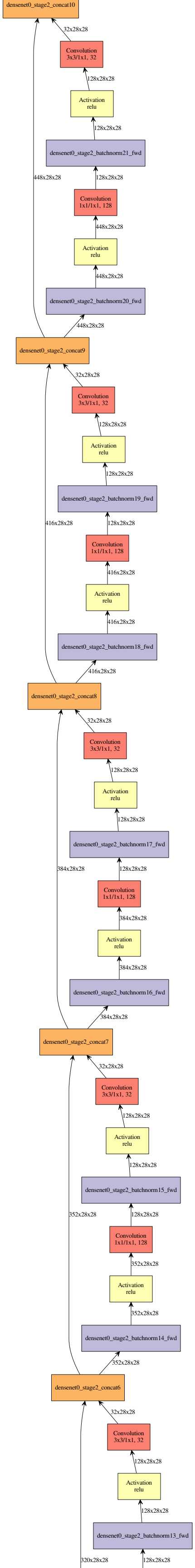
densenet0_stage2_concat10
32x28x28
Convolution
3x3/1x1, 32
128x28x28
Activation
relu
128x28x28
densenet0_stage2_batchnorm21_fwd
128x28x28
Convolution
1x1/1x1, 128
448x28x28
Activation
relu
448x28x28
densenet0_stage2_batchnorm20_fwd
448x28x28
448x28x28
densenet0_stage2_concat9
32x28x28
Convolution
3x3/1x1, 32
128x28x28
Activation
relu
128x28x28
densenet0_stage2_batchnorm19_fwd
128x28x28
Convolution
1x1/1x1, 128
416x28x28
Activation
relu
416x28x28
densenet0_stage2_batchnorm18_fwd
416x28x28
416x28x28
densenet0_stage2_concat8
32x28x28
Convolution
3x3/1x1, 32
128x28x28
Activation
relu
128x28x28
densenet0_stage2_batchnorm17_fwd
128x28x28
Convolution
1x1/1x1, 128
384x28x28
Activation
relu
384x28x28
densenet0_stage2_batchnorm16_fwd
384x28x28
384x28x28
densenet0_stage2_concat7
32x28x28
Convolution
3x3/1x1, 32
128x28x28
Activation
relu
128x28x28
densenet0_stage2_batchnorm15_fwd
128x28x28
Convolution
1x1/1x1, 128
352x28x28
Activation
relu
352x28x28
densenet0_stage2_batchnorm14_fwd
352x28x28
352x28x28
densenet0_stage2_concat6
32x28x28
Convolution
3x3/1x1, 32
128x28x28
Activation
relu
128x28x28
densenet0_stage2_batchnorm13_fwd
320x28x28
128x28x28

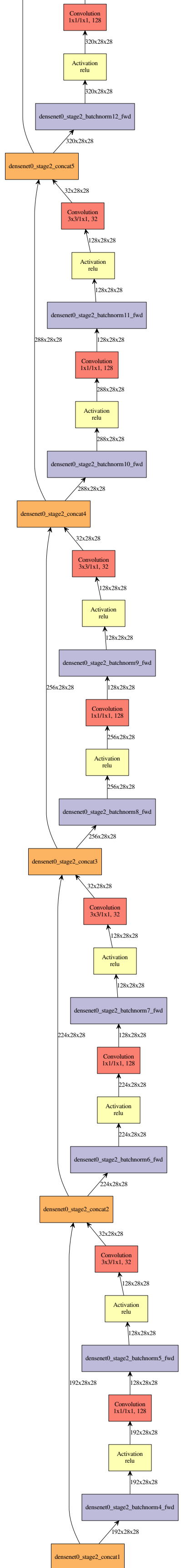

Convolution
1x1/1x1, 128
320x28x28
Activation
relu
320x28x28
densenet0_stage2_batchnorm12_fwd
320x28x28
densenet0_stage2_concat5
32x28x28
Convolution
3x3/1x1, 32
128x28x28
Activation
relu
128x28x28
densenet0_stage2_batchnorm11_fwd
288x28x28
128x28x28
Convolution
1x1/1x1, 128
288x28x28
Activation
relu
288x28x28
densenet0_stage2_batchnorm10_fwd
288x28x28
densenet0_stage2_concat4
32x28x28
Convolution
3x3/1x1, 32
128x28x28
Activation
relu
128x28x28
densenet0_stage2_batchnorm9_fwd
256x28x28
128x28x28
Convolution
1x1/1x1, 128
256x28x28
Activation
relu
256x28x28
densenet0_stage2_batchnorm8_fwd
256x28x28
densenet0_stage2_concat3
32x28x28
Convolution
3x3/1x1, 32
128x28x28
Activation
relu
128x28x28
densenet0_stage2_batchnorm7_fwd
224x28x28
128x28x28
Convolution
1x1/1x1, 128
224x28x28
Activation
relu
224x28x28
densenet0_stage2_batchnorm6_fwd
224x28x28
densenet0_stage2_concat2
32x28x28
Convolution
3x3/1x1, 32
128x28x28
Activation
relu
128x28x28
densenet0_stage2_batchnorm5_fwd
192x28x28
128x28x28
Convolution
1x1/1x1, 128
192x28x28
Activation
relu
192x28x28
densenet0_stage2_batchnorm4_fwd
192x28x28
densenet0_stage2_concat1

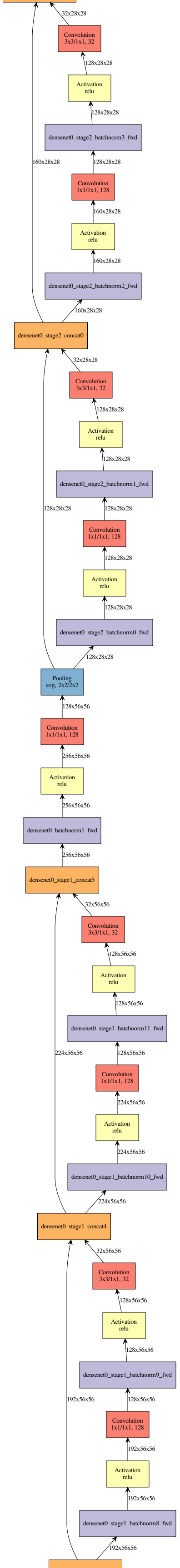
32x28x28
Convolution 3x3/1x1, 32
128x28x28
Activation relu
128x28x28
densenet0_stage2_batchnorm3_fwd
128x28x28
160x28x28
Convolution 1x1/1x1, 128
160x28x28
Activation relu
160x28x28
densenet0_stage2_batchnorm2_fwd
160x28x28
densenet0_stage2_concat0
32x28x28
Convolution 3x3/1x1, 32
128x28x28
Activation relu
128x28x28
densenet0_stage2_batchnorm1_fwd
128x28x28
128x28x28
Convolution 1x1/1x1, 128
128x28x28
Activation relu
128x28x28
densenet0_stage2_batchnorm0_fwd
128x28x28
Pooling avg, 2x2/2x2
128x56x56
Convolution 1x1/1x1, 128
256x56x56
Activation relu
256x56x56
densenet0_batchnorm1_fwd
256x56x56
densenet0_stage1_concat5
32x56x56
Convolution 3x3/1x1, 32
128x56x56
Activation relu
128x56x56
densenet0_stage1_batchnorm11_fwd
128x56x56
224x56x56
Convolution 1x1/1x1, 128
224x56x56
Activation relu
224x56x56
densenet0_stage1_batchnorm10_fwd
224x56x56
densenet0_stage1_concat4
32x56x56
Convolution 3x3/1x1, 32
128x56x56
Activation relu
128x56x56
densenet0_stage1_batchnorm9_fwd
128x56x56
192x56x56
Convolution 1x1/1x1, 128
192x56x56
Activation relu
192x56x56
densenet0_stage1_batchnorm8_fwd
192x56x56

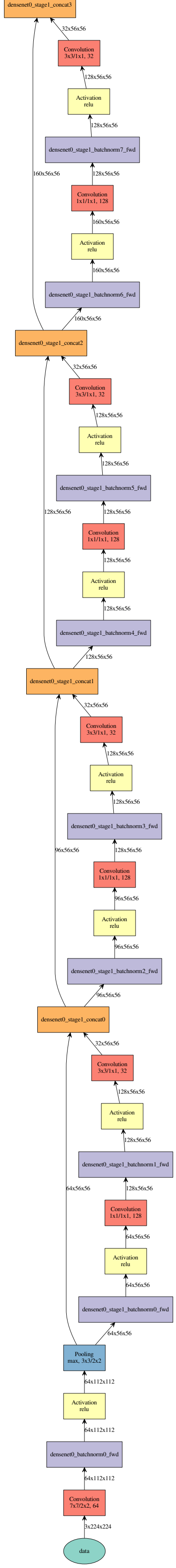
densenet0_stage1_concat3
32x56x56
Convolution
3x3/1x1, 32
128x56x56
Activation
relu
128x56x56
densenet0_stage1_batchnorm7_fwd
160x56x56
128x56x56
Convolution
1x1/1x1, 128
160x56x56
Activation
relu
160x56x56
densenet0_stage1_batchnorm6_fwd
160x56x56
densenet0_stage1_concat2
32x56x56
Convolution
3x3/1x1, 32
128x56x56
Activation
relu
128x56x56
densenet0_stage1_batchnorm5_fwd
128x56x56
128x56x56
Convolution
1x1/1x1, 128
128x56x56
Activation
relu
128x56x56
densenet0_stage1_batchnorm4_fwd
128x56x56
densenet0_stage1_concat1
32x56x56
Convolution
3x3/1x1, 32
128x56x56
Activation
relu
128x56x56
densenet0_stage1_batchnorm3_fwd
96x56x56
128x56x56
Convolution
1x1/1x1, 128
96x56x56
Activation
relu
96x56x56
densenet0_stage1_batchnorm2_fwd
96x56x56
densenet0_stage1_concat0
32x56x56
Convolution
3x3/1x1, 32
128x56x56
Activation
relu
128x56x56
densenet0_stage1_batchnorm1_fwd
64x56x56
128x56x56
Convolution
1x1/1x1, 128
64x56x56
Activation
relu
64x56x56
densenet0_stage1_batchnorm0_fwd
64x56x56
Pooling
max, 3x3/2x2
64x112x112
Activation
relu
64x112x112
densenet0_batchnorm0_fwd
64x112x112
Convolution
7x7/2x2, 64
3x224x224
data
